# Risk-based lung cancer screening in heavy smokers: a benefit–harm and cost-effectiveness modeling study

**DOI:** 10.1186/s12916-024-03292-4

**Published:** 2024-02-19

**Authors:** Yin Liu, Huifang Xu, Lihong Lv, Xiaoyang Wang, Ruihua Kang, Xiaoli Guo, Hong Wang, Liyang Zheng, Hongwei Liu, Lanwei Guo, Qiong Chen, Shuzheng Liu, Youlin Qiao, Shaokai Zhang

**Affiliations:** 1 Department of Cancer Epidemiology, The Affiliated Cancer Hospital of Zhengzhou University & Henan Cancer Hospital, Zhengzhou, 450008 China; 2 Center for Global Health, School of Population Medicine and Public Health, Chinese Academy of Medical Sciences and Peking Union Medical College, Beijing, 100005 China

**Keywords:** Lung cancer, Risk-based screening, Benefit, Harm, Cost-effective

## Abstract

**Background:**

Annual screening through low-dose computed tomography (LDCT) is recommended for heavy smokers. However, it is questionable whether all individuals require annual screening given the potential harms of LDCT screening. This study examines the benefit–harm and cost-effectiveness of risk-based screening in heavy smokers and determines the optimal risk threshold for screening and risk-stratified screening intervals.

**Methods:**

We conducted a comparative cost-effectiveness analysis in China, using a cohort-based Markov model which simulated a lung cancer screening cohort of 19,146 heavy smokers aged 50 ~ 74 years old, who had a smoking history of at least 30 pack-years and were either current smokers or had quit for < 15 years. A total of 34 risk-based screening strategies, varying by different risk groups for screening eligibility and screening intervals (1-year, 2-year, 3-year, one-off, non-screening), were evaluated and were compared with annual screening for all heavy smokers (the status quo strategy). The analysis was undertaken from the health service perspective with a 30-year time horizon. The willingness-to-pay (WTP) threshold was adopted as three times the gross domestic product (GDP) of China in 2021 (CNY 242,928) per quality-adjusted life year (QALY) gained.

**Results:**

Compared with the status quo strategy, nine risk-based screening strategies were found to be cost-effective, with two of them even resulting in cost-saving. The most cost-effective strategy was the risk-based approach of annual screening for individuals with a 5-year risk threshold of ≥ 1.70%, biennial screening for individuals with a 5-year risk threshold of 1.03 ~ 1.69%, and triennial screening for individuals with a 5-year risk threshold of < 1.03%. This strategy had the highest incremental net monetary benefit (iNMB) of CNY 1032. All risk-based screening strategies were more efficient than the status quo strategy, requiring 129 ~ 656 fewer screenings per lung cancer death avoided, and 0.5 ~ 28 fewer screenings per life-year gained. The cost-effectiveness of risk-based screening was further improved when individual adherence to screening improved and individuals quit smoking after being screened.

**Conclusions:**

Risk-based screening strategies are more efficient in reducing lung cancer deaths and gaining life years compared to the status quo strategy. Risk-stratified screening intervals can potentially balance long-term benefit–harm trade-offs and improve the cost-effectiveness of lung cancer screenings.

**Supplementary Information:**

The online version contains supplementary material available at 10.1186/s12916-024-03292-4.

## Background

Lung cancer is the leading cause of cancer deaths in China and worldwide. According to the National Central Cancer Registry of China, in 2022, approximately 733,300 people died from lung cancer, accounting for 28.5% of all cancer deaths [1]. Screening through low-dose computed tomography (LDCT) is recommended to reduce lung cancer mortality [2]. However, despite the potential benefits of LDCT screening in lung cancer mortality reduction, many screening trials have also demonstrated the risks of potential harms [3], such as false positives, overdiagnosis, and elevated cancer incidence from radiation exposure. Consequently, questions remain on how to implement lung cancer screening to improve the benefit–harm trade-offs and cost-effectiveness.

Annual screening for LDCT is recommended to heavy smokers in most guidelines and screening trials because of their high risk of lung cancer, based on age, cumulative pack years, and years since quitting smoking [4–7]. For example, the latest Chinese guideline in 2021 (the status quo strategy, hereafter called “China’s 2021 guideline recommendation”) recommends annual LDCT screening for individuals aged 50 ~ 74 years old, who smoke at least 30 pack-years and currently smoke or quit less than 15 years ago [7]. The strategy is simple but focuses on the lung cancer risk of screening eligible smokers as a whole. However, most individuals will never develop lung cancer but may experience harm [2, 8]. Furthermore, it is questionable whether all individuals require annual screening given the potential harms thereof. A retrospective cohort analysis of data from the National Lung Screening Trial (NLST) found that individuals with a negative LDCT prevalence screen had a lower lung cancer incidence and mortality than did all participants who underwent a prevalence screen, and suggested risk-stratified screening intervals were required to balance the benefits and harms of LDCT screening [9].

Recently, evidence for a more personalized screening strategy based on established lung cancer risk-prediction models has become available “Risk-based screening” section. Risk-based screening can be used to further stratify and precisely select individuals according to the estimated risk and has suggested superiority over the status quo strategy in identifying eligible individuals who are most likely to benefit from LDCT screening [10–12]. However, there are some questions raised. First, little is known about the long-term benefits, harms, and cost-effectiveness of a risk-based screening strategy. Individuals with higher risk are older and thus have shorter life expectancy, potentially affecting the long-term benefits, harms, and cost-effectiveness of LDCT screening [11, 13]. Second, the optimal risk threshold for LDCT screening from a cost-effectiveness perspective is rarely determined. The threshold should provide a good balance between health outcomes and costs to tolerate a degree of imprecision in the estimation of individual risk [14]. However, most studies have only identified the risk threshold that would either match the sensitivity or select a similar number or proportion of eligible individuals to the current guideline recommendations [15–17]. Third, risk-stratified screening intervals should be further refined. Despite many studies comparing different screening intervals, only the population as a whole was analyzed, and the focus was on stratification to either annual, biennial, or triennial screening [18–20].

Therefore, based on a LDCT screening cohort of heavy smokers in China, we aimed to conduct this modeling study to evaluate the long-term benefits, harms, and cost-effectiveness of risk-based screening. Additionally, we aimed to determine the optimal risk threshold for LDCT screening and the risk-stratified screening intervals for individuals at different risk levels.

## Methods

### LDCT screening cohort

For the current analysis, we aimed to model a LDCT screening cohort of heavy smokers in China aged 50 ~ 74 years old. Heavy smokers were defined as those who had a smoking history of at least 30 pack-years and were either current smokers or had quit for < 15 years.

The cohort was generated from an ongoing cancer screening program started in urban China (CanSPUC) in October 2012, targeting five kinds of cancers (lung cancer, female breast cancer, liver cancer, colorectal cancer, and upper gastrointestinal cancer). The detailed methodology of CanSPUC has been previously described [21]. Briefly, residents living in the selected cities were enrolled by phone and personal contact. After signing written informed consent, all eligible participants were required to complete a questionnaire about their exposure to risk factors. Only those participants with a high risk confirmed by a defined clinical cancer risk system were recommended to undergo screening intervention. For lung cancer screening, a one-round LDCT examination, free of charge at a tertiary-level hospital designated by the program, was recommended to those participants at high risk of lung cancer. Individuals were followed up annually until the occurrence of the first diagnosis of cancer or loss to follow-up, death, or administrative censoring, whichever transpired first. Incidental lung cancer cases were collected by follow-up as well as linking to the provincial cancer registry database for all individuals.

This study recruited 19,146 asymptomatic heavy smokers aged 50 ~ 74 years who participated in the CanSPUC program and underwent LDCT lung cancer screening in Henan Province, China, between October 2013 and March 2020. The total cohort included 18,560 men and 586 women, with a median of 40 pack-years and an average age of 59.7 ± 6.01 years. Of these, 112 cases occurred during the following-up period, yielding an incidence density of 178.43/100,000 person-years.

### Estimation of individual absolute 5-year risk of developing lung cancer

We used a previously published relative risk model for lung cancer based on the LDCT screening cohort to measure individual absolute risk [21]. The model included age, sex, smoking intensity (pack-years), self-reported history of tuberculosis, and history of emphysema. The selected risk factors and their corresponding hazard ratios are listed in Additional file 1: Table S1. The C-index of the model was 0.750, 0.721, and 0.751 for 1-, 3-, and 5-year lung cancer risk in ever-smokers, respectively. Thus, in this study, the individual 5-year absolute risk of lung cancer was projected using a method similar to that described by Gail et al. [22]. Briefly, the absolute 5-year risk that an individual aged *a* will develop lung cancer in 5 years is $P\left(a,r,j\right)=\left[{h}_{1j}r/({h}_{1j}r+{h}_{2j})\right][1-exp\left\{-5\left({h}_{1j}r+{h}_{2j}\right)\right\}]$, where *h*_1*j*_ is the baseline hazard of developing lung cancer for men, *j* = 1, and women, *j* = 2; *r* is the relative risk of lung cancer compared to an individual with no risk factors; and *h*_2*j*_ is the age- and sex-specific mortality rate of non-lung cancer. To have a robust and generalizable model, the baseline hazards, *h*_1*j*_, were calculated by multiplying the age- and sex-specific incidence rates in 2017 from the National Central Cancer Registry of China (NCCR) [23] by 1 minus the population-attributable risk [12]. The PAR was estimated using the formula described by Bruzzi et al. [24] and could be interpreted as the fraction in the incidence of lung cancer that would be reduced during follow-up if the risk factors in the relative risk model took the lowest risk category. *h*_2*j*_ was calculated as age- and sex-specific all-cause mortality rates in 2020 from the China Population Census Yearbook [25] minus age- and sex-specific lung cancer mortality rates in 2017 from NCCR [23]. Because only heavy smokers were eligible for this current study, all-cause mortality rates were adjusted using a relative risk (1.33 for males, 1.44 for females) [26]. The estimated incidence of lung cancer and mortality rates of non-lung cancer by sex and age are presented in Additional file 1: Table S2.

In this study, the estimated absolute 5-year risk of lung cancer for individuals varied from 0.115 to 11.395%. Quartiles were used to stratify individuals into four equal groups based on their absolute 5-year risk: high (“H”), medium–high (“MH”), low-medium (“LM”), and low (“L”). The corresponding risk thresholds for the four risk groups were ≥ 1.70%, 1.03 ~ 1.69%, 0.49 ~ 1.02%, and < 0.49%, respectively.

### Lung cancer cohort-based Markov model overview

**Fig. 1:**
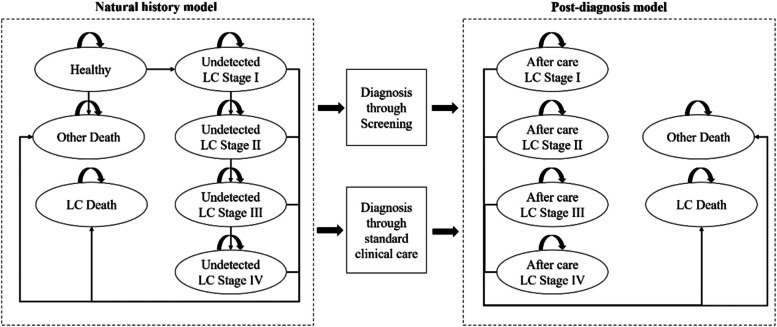
Structure of Markov process model. LC, lung cancer

A cohort-based Markov model was modified based on the validated model developed by Hofer et al. [27]. The model consisted of two separate parts to distinguish between the (i) natural history model and (ii) post-diagnosis model. We assumed that patients were immediately treated after being diagnosed. The structure of this model is shown in Fig. 1.

The natural history model included seven states: one healthy state in terms of lung cancer (Healthy), four undetected lung cancer stages (LC Stage I ~ LC Stage IV), death caused by lung cancer (LC Death), death by other causes except lung cancer (Other Death). Individuals in a healthy state may develop undetected lung cancer stages, remain in the current stage, or die from other causes. Individuals who developed lung cancer may progress to any higher stage, remain in the current stage, get diagnosed through screening or standard clinical care (i.e., when they were symptomatic), or die.

The post-diagnosis model included six states: four after-care lung cancer stages (LC Stage I ~ LC Stage IV), death caused by lung cancer (LC Death), and death by other causes except lung cancer (Other Death). We did not implicitly include recurrence after treatment, and patients in after-care lung cancer stages are expected to remain in the current stage, or die.

In this study, lung cancer stages IA, IB, IIIA, and IIIB were not considered due to the data being unavailable for clinical practice in population-based cancer registries in China. In any of the states, patients with lung cancer were at risk of all-cause mortality.

### Screening strategies

In total, 36 screening strategies were evaluated, including no screening, the status quo strategy, and 34 risk-based screening strategies. Detailed information is shown in Additional file 1: Table S3.

#### No screening

The individuals would not be screened, but lung cancer could be detected through standard clinical care.

#### China’s 2021 guideline recommendation (status quo strategy)

All the individuals would be screened annually, with a starting age of 50 years and stopping age of 74 years, as recommended in the current guideline (“H1-MH1-LM1-L1”).

#### Risk-based screening

At baseline, individual absolute 5-year risk of developing lung cancer was calculated, and then, LDCT screening decisions, including whether to screen and screening intervals, were made based on individual risk. We assumed that the high-risk group was screened at 1-year intervals because of their high risk of lung cancer; the lower-risk group was not screened more frequently than the higher-risk group. Considering different combinations of risk thresholds for screening eligibility and intervals (1-year, 2-year, 3-year, one-off, non-screening), a total of 34 risk-based screening strategies were evaluated.

### Input parameters and assumption

Input parameters were extracted from the LDCT screening cohort results, sample survey, and other published evidence, where available. A probability distribution around the expected value was set for input parameters where there was uncertainty. The key input parameters are summarized in Table 1.

**Table 1:** Input parameters of Markov model for lung cancer screening

Parameters	Base case (range)	Distribution	Reference
**Lung cancer incidence**	Changing with age and smoking intensity	**-**	LDCT screening cohort
**Transition probabilities**			
Lung cancer stage I to stage II	0.3682 (± 50%)	Beta (9.33, 16.01)	[28]
Lung cancer stage I to stage III	0.0328 (± 50%)	Beta (14.83, 437.29)	
Lung cancer stage I to stage IV	0.0745 (± 50%)	Beta (14.15, 175.75)	
Lung cancer stage II to stage III	0.2260 (± 50%)	Beta (11.67, 39.96)	
Lung cancer stage II to stage IV	0.1510 (± 50%)	Beta (12.89, 72.50)	
Lung cancer stage III to stage IV	0.1455 (± 50%)	Beta (12.98, 76.26)	
**Mortality**			
Mortality of non-lung cancer death	See Table S2		
Lung cancer stage I to LC death	0.1739 (± 50%)	Beta (12.52, 59.48)	[29, 30]
Lung cancer stage II to LC death	0.2942 (± 50%)	Beta (10.55, 25.31)	
Lung cancer stage III to LC death	0.4626 (± 50%)	Beta (7.79, 9.06)	
Lung cancer stage IV to LC death	0.5880 (± 50%)	Beta (5.74, 4.02)	
Aftercare I to death	0.089 (± 50%)	Beta (13.91, 142.23)	[31]
Aftercare II to death	0.153 (± 50%)	Beta (12.86, 71.32)	
Aftercare III to death	0.288 (± 50%)	Beta (10.65, 26.34)	
Aftercare IV to death	0.353 (± 50%)	Beta (9.59, 17.60)	
**Performance of LDCT**			
Sensitivity of LDCT	0.937 (0.890–1.000)	Beta (649, 44)	[32]
Specificity of LDCT	0.765 (0.700–0.930)	Beta (56,936, 17,497)	
Overdiagnosis rate when screening	0.031 (± 50%)	Beta (14.86, 464.46)	
Excess relative risk of LC per screening	0.001 (0.0003–0.0019)	Beta (6, 5995)	[33, 34]
**Adherence to LDCT screening**	100% (50–100%)	Triangle (0.5, 1, 1)	Assumption
**Diagnose rate through standard clinical care**			
Lung cancer stage I	2.46% (± 50%)	Beta (14.96, 593.32)	[27]
Lung cancer stage II	2.7% (± 50%)	Beta (14.91, 537.48)	
Lung cancer stage III	51.8% (± 50%)	Beta (6.89, 6.41)	
Lung cancer stage IV	65.8% (± 50%)	Beta (4.60, 2.39)	
**Cost (CNY)**			
Pre-diagnosis cost (cost of scoring questionnaire and risk stratification)	613.3 (± 50%)	Gamma (15.37, 0.02)	[28]
LDCT test cost	239.97 (± 50%)	Gamma (15.37, 0.06)	
Biopsy diagnosis cost	1202.9 (± 50%)	Gamma (15.37, 0.01)	
Lung cancer stage I first year	83984.91 (± 50%)	Gamma (15.37, 1.83)	Sample survey
Lung cancer stage I second year and beyond	19967.12 (± 50%)	Gamma (15.37, 7.70)	Sample survey
Lung cancer stage II first year	97435.6 (± 50%)	Gamma (15.37, 1.58)	Sample survey
Lung cancer stage II second year and beyond	15888.82 (± 50%)	Gamma (15.37, 9.67)	Sample survey
Lung cancer stage III first year	90813.2 (± 50%)	Gamma (15.37, 1.69)	Sample survey
Lung cancer stage III second year and beyond	32472.69 (± 50%)	Gamma (15.37, 4.73)	Sample survey
Lung cancer stage IV first year	86484.70 (± 50%)	Gamma (15.37, 1.78)	Sample survey
Lung cancer stage IV second year and beyond	54962.59 (± 50%)	Gamma (15.37, 2.80)	Sample survey
Background medical treatment costs	5348.1 (± 50%)	Gamma (15.37, 0.003)	[35]
**Utility**			
Utility for healthy smokers without disease (tuberculosis, emphysema, lung cancer), by sex and age			
Male, aged 40–50	0.99 (0.987–0.994)	Beta (4355.01, 43.99)	[36]
Male, aged 51–60	0.984 (0.980–0.988)	Beta (3872.04, 62.96)	
Male, aged 61–70	0.976 (0.971–0.980)	Beta (3514.87, 86.43)	
Male, aged $\ge$ 71	0.947 (0.936–0.958)	Beta (1514.71, 84.77)	
Female, aged 40–50	0.988 (0.986–0.991)	Beta (11,257.88, 136.74)	
Female, aged 51–60	0.982 (0.979–0.986)	Beta (7713.61, 141.39)	
Female, aged 61–70	0.964 (0.958–0.971)	Beta (3480.26, 129.97)	
Female, aged $\ge$ 71	0.936 (0.926–0.946)	Beta (2153.09, 147.22)	
Disutility associated with tuberculosis	0.01 (± 50%)	Beta (15.20, 1505.07)	[37, 38]
Disutility associated with emphysema	0.052 (± 50%)	Beta (14.51, 264.63)	
Utility of lung cancer by stage			
Lung cancer stage I	0.85 (0.78–0.89)	Beta (136.78, 24.14)	[39–41]
Lung cancer stage II	0.75 (0.68–0.80)	Beta (149.31, 49.77)	
Lung cancer stage III	0.69 (0.56–0.79)	Beta (42.18, 18.95)	
Lung cancer stage IV	0.69 (0.38–0.70)	Beta (21.46, 9.64)	
Disutility associated with a false-positive result	0.063 (± 50%) for 3 months	Beta (14.33, 213.21)	[42]
**Discount rate**	5% (0–8%)	Triangle (0, 0.05, 0.08)	[43]

#### Incidence

According to the relative risk model for lung cancer described above, the incidence of lung cancer changes with age and smoking intensity. Therefore, the incidence in the cycle *i* (*I*_i_) was modeled using a multiplicative function as ${I}_{i}={I}_{b}\times {HR}_{i}$, where *I*_b_ is the baseline incidence adopted from the LDCT screening cohort results; *HR*_*i*_ is the hazard ratio for the incidence in the cycle, *i*, compared with the baseline incidence and it is extracted from the relative risk model described above. The cycle refers to the sequence of events or transitions that occur within the Markov model. It represents the period of time it takes for the model to go through all possible states and return to its initial state. In the base-case analysis, we assumed that individual smoking behaviors would not be changed. A sensitivity analysis was also conducted with an assumption that individuals voluntarily quit smoking with the implementation of LDCT screening, and lung cancer incidence only increases with age.

#### Transition probabilities between lung cancer stages

Individuals with lung cancer may progress to any higher stage. The 1-year transition probabilities between lung cancer stages in the natural model were obtained from published evidence.

#### Mortality

We assumed that mortality rates of non-lung cancer varied by age and sex, but would not differ among the states. Age- and sex-specific mortality rates of non-lung cancer were set as *h*_2*j*_, as described above. Lung cancer stage-specific mortality rates in the natural history model were derived from a study by Zhang et al. [29] and were adjusted for smoking status [30]. Lung cancer stage-specific mortality rates in the post-diagnosis model were converted from 5-year survival rates based on a large-sample survival study of lung cancer patients in China [31]. The study determined the survival status of patients with lung cancer during 2001 to 2018 based on the registration and survival follow-up report data of 16,188 cases collected from the Chongqing cancer registration system.

#### Performance of LDCT

The sensitivity and specificity of LDCT and overdiagnosis rate were obtained through the NLST results [32]. The excess relative risk of lung cancer per screening caused by ionizing radiation from LDCT was calculated by multiplying the radiation exposure dose of a single examination [33] by the increased risk induced by per unit exposure dose [34].

#### Adherence to LDCT screening and diagnosis rate through standard clinical care

We assumed full adherence to LDCT screening in order to accurately estimate the screening outcomes for individuals who are willing to undergo the screening. Sensitivity analyses were also conducted to explore the effect of imperfect adherence rates on the outcomes. The model assumed that in the absence of active screening, individuals with stage I ~ IV lung cancer would receive a diagnosis through standard clinical care, based on stage-specific probabilities [27].

#### Cost

A health system perspective was used to collect direct medical costs, including pre-diagnosis and diagnosis-related, lung cancer care-related, and background medical treatment costs. Pre-diagnosis and diagnosis-related costs were taken from the CanSPUC program [28]. Lung cancer care costs were collected through a sample survey, with the detailed design of this survey outlined in Additional file 2. In brief, lung cancer care costs were calculated by stage, including outpatient visits; hospitalization and all types of therapy (i.e., surgery, chemotherapy, radiotherapy, and supportive care) were estimated by reviewing the medical records of a sample of 350 patients admitted to the Henan cancer hospital. This is a public, specialized, educational hospital for care of patients with all types of cancers. Lung cancer care costs were differentiated for a period of 1 year from the beginning of each stage and for the second and following years and were estimated using hospital bills. We assumed that the undetected lung cancer cases in the natural history model were expected to receive background medical treatment because of signs or symptoms of lung disease (i.e., cough, fatigue, hemoptysis, etc.). The background medical treatment costs were based on national per capita health expenditure in 2022 from the China Health Statistics Yearbook [35]. All costs in this study were converted to 2022 Chinese yuan (CNY) using the medical component of the consumer price index.

#### Utility

Utility scores were estimated by age, sex, and disease (tuberculosis, emphysema, lung cancer). We assumed that utility scores for healthy smokers without disease were similar to the general population, obtained from a study using EQ-5D-5L in a total of 10,056 general adults in China [36]. Disutility was applied for smokers with tuberculosis or emphysema but without lung cancer, and the scores were estimated from a published large-sample study or a meta-analysis [37, 38]. The utility of lung cancer by stage was obtained from a published meta-analysis [39] and epidemiological survey among Chinese lung cancer patients [40, 41]. A significant disutility of 0.063 lasting for 3 months was applied for a false-positive result [42].

### Model outcomes

The primary outcome measures included the following: (1) the incremental net monetary benefit (iNMB) compared to the status quo strategy; (2) the cost-effectiveness efficiency frontier, in which line segments connect strategies that yield the highest health benefit at a given level of cost; and (3) the incremental cost-effectiveness ratio (ICER) of the different screening strategies compared with status quo strategy, and the strategy preceding it on the deficiency frontier. The iNMB is calculated by multiplying the mean incremental quality-adjusted life years (QALYs) by a given willingness-to-pay (WTP) threshold and subtracting the total incremental cost [44]. This measure allows us to summarize both the health and economic gains of an intervention on a common scale. A positive iNMB indicates that the intervention strategy is cost-effective. The ICER is the difference in cost between two strategies divided by the difference in QALYs. According to the World Health Organization recommendations, three times the gross domestic product (GDP) of China in 2021 (CNY 80,976) per QALY gained was applied as the WTP threshold to define a cost-effective strategy. The screening strategy with the highest iNMB for a given WTP threshold was deemed the most cost-effective approach.

Secondary outcomes included the number of LDCT screenings, the number of lung cancer deaths averted, lung cancer mortality reduction, life years gained from screening, and the number of false-positive findings, over-diagnosed lung cancer cases (defined as the detection of lung cancer through LDCT screening that would not have become clinical apparent), and radiation-related lung cancer. Outcomes were provided per 100,000 individuals. Average number screens per lung cancer death averted and average number screens per life-year gained were also calculated.

### Statistical analysis

The Markov model and statistical analyses were performed using TreeAge Pro 2022 and R software V 4.3, and graph plotting was done with Excel software.

Descriptive statistical techniques were employed to analyze the baseline characteristics of the four risk groups. Categorical variables were described using frequency and percentage. Quantitative variables that exhibited a normal distribution were described using the mean and standard deviation (SD). For quantitative variables that did not follow a normal distribution, the 50th percentile (P_50_), 25th percentile (P_25_), and 75th percentile (P_75_) were used for description.

All future costs and utility scores were discounted at a 5% (0–8%) annual rate [43]. We chose a cycle length of 1 year and ran the Markov model for 30 cycles; half-cycle correction was applied. Additional one-way and probabilistic sensitivity analyses were conducted to explore the effect of input parameter uncertainty on cost-effectiveness, wherein we varied the value of key input parameters including sensitivity of LDCT, specificity of LDCT, overdiagnosis rate when screening, excess relative risk of lung cancer per screening, LDCT test cost, background medical treatment costs, disutility associated with a false-positive screen, imperfect adherence rate to LDCT screening, and discount rate. We also evaluated the cost-effectiveness of screening strategies with the assumption that individuals quit smoking after being screened. In the probabilistic sensitivity analyses, we performed a second-order Monte Carlo simulation with 100,000 iterations to address the joint uncertainties in the values of the input parameters [45], and the cost-effectiveness acceptability curves were plotted to show the proportion of simulations for which screening scenario was cost-effective at different WTPs.

## Results

### Study population

After stratifying the 19,146 asymptomatic heavy smokers by their baseline absolute 5-year risk of developing lung cancer, the high-risk versus low-risk quartile participants were on average 15 years older, were more likely to be a smoker with a 50 pack-year or greater exposure, have a history of tuberculosis, and have a history of emphysema. Baseline lung cancer incident density of the high-, medium–high-, low-medium-, and low-risk groups were 262, 220, 154, and 81 per 100,000 person-years, respectively (Table 2).

**Table 2:** Baseline characteristics and incident density by the four risk groups

Characteristics	High-risk, *n* (%)	Medium–high-risk, *n* (%)	Low-medium-risk, *n* (%)	Low-risk, *n* (%)
**Age (years)**				
50–54	0 (0)	0 (0)	84 (1.8)	4658 (97.5)
55–59	46 (0.9)	272 (5.7)	4337 (94.7)	118 (2.5)
60–64	449 (8.9)	4467 (93.6)	90 (2.0)	0 (0)
65–69	3451 (68.8)	6 (0.1)	69 (1.5)	0 (0)
70–74	1072 (21.4)	27 (0.6)	0 (0)	0 (0)
Mean $\pm$ SD	67.2 $\pm$ 3.0	61.7 $\pm$ 2.0	57.0 $\pm$ 2.1	52.3 $\pm$ 1.5
**Sex**				
Female	30 (0.6)	61 (1.3)	210 (4.6)	285 (6.0)
Male	4988 (99.4)	4711 (98.7)	4370 (95.4)	4491 (94.0)
**Smoking intensity (pack years)**				
30–49	3090 (61.6)	3168 (66.4)	3218 (70.3)	3402 (71.2)
$\ge$ 50	1928 (38.4)	1604 (33.6)	1362 (29.7)	1374 (28.8)
P_50_ (P_25_,P_75_)	40.0 (35.0, 60.0)	40.0 (32.0, 60.0)	40.0 (30.0, 53.0)	36.0 (30.0, 53.0)
**Self-reported history of tuberculosis**				
No	4597 (91.6)	4700 (98.5)	4421 (96.5)	4625 (96.8)
Yes	421 (8.4)	72 (1.5)	159 (3.5)	151 (3.2)
**Self-reported history of emphysema**				
No	4336 (86.4)	4538 (95.1)	4474 (97.7)	4577 (95.8)
Yes	682 (13.6)	234 (4.9)	106 (2.3)	199 (4.2)
**Incident density (/100,000 person-years)**	262	220	154	81

The corresponding risk thresholds for the high-, medium–high-, low-medium-, and low-risk groups were ≥ 1.70%, 1.03 ~ 1.69%, 0.49 ~ 1.02%, and < 0.49%, respectively

### Cost-effectiveness of screening

**Fig. 2:**
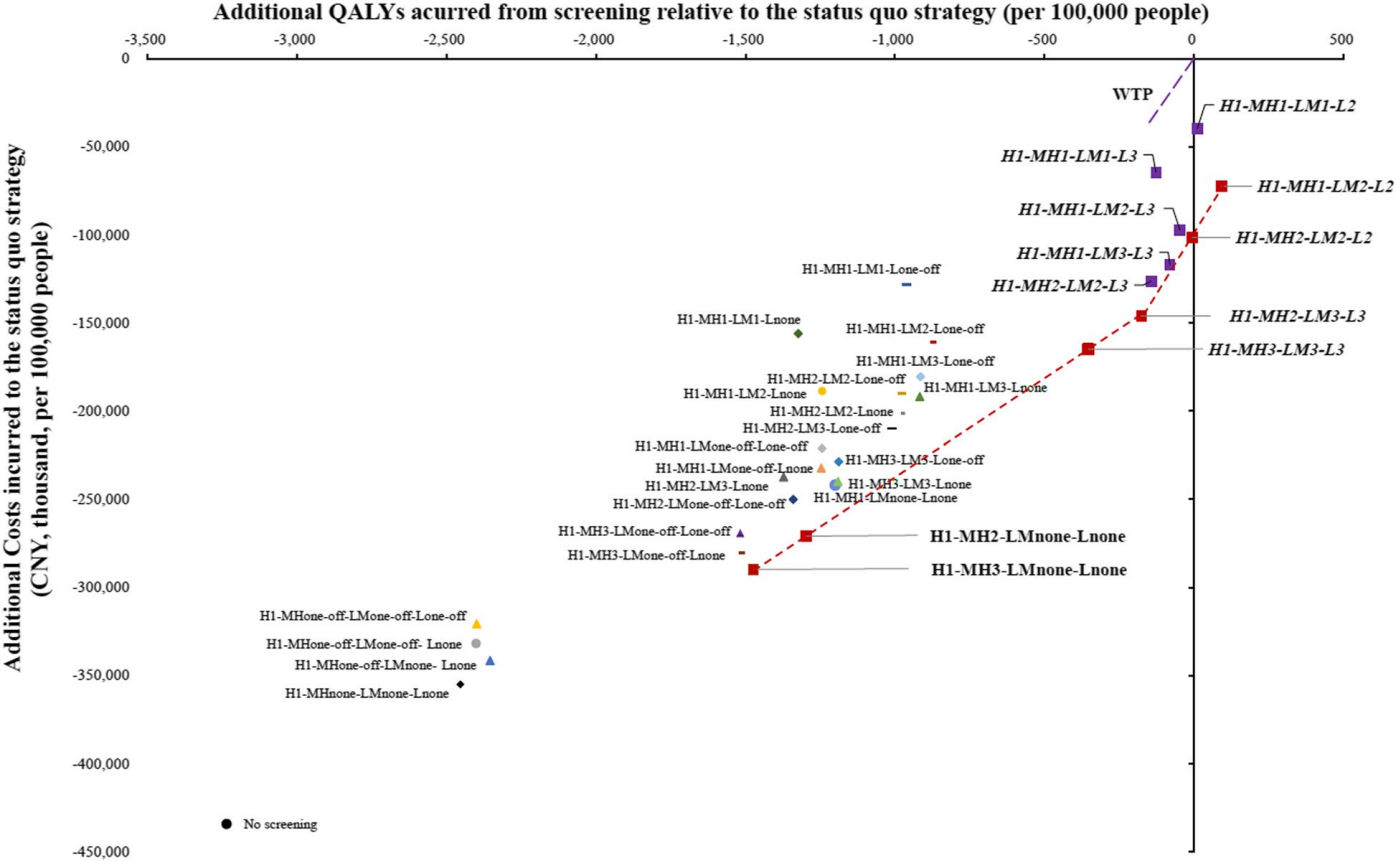
Cost-effectiveness efficiency frontier for lung cancer screening strategies. The red box represents the strategies on the cost-effectiveness efficiency frontier. Strategies in the italic text are the strategies dominant to the status quo strategy (H1-MH1-LM1-L1). QALY, quality-adjusted life year; WTP, willingness-to-pay

At the WTP threshold of CNY 242,928 per QALY gained, overall screening strategies gained more QALYs and more iNMB than no screening. The strategy of H1-MH1-LM2-L2 generated the maximum QALYs of 1,153,692 per 100,000 people over 30 years. Compared with the status quo strategy, the risk-based screening strategies cost CNY 397.87 ~ 3549.23 less per person, and nine strategies were dominant; of these, two strategies (H1-MH1-LM1-L2, H1-MH1-LM2-L2) were cost-saving (that is, were less costly yet yielded more QALYs). The cost-effectiveness efficiency frontier derived from the analysis included six risk-based strategies, and H1-MH2-LM3-L3 was the most cost-effective with the largest iNMB of CNY 1032, followed by H1-MH2-LM2-L2 (CNY 1002) (Table 3 and Fig. 2).

**Table 3:** Cost-effectiveness estimates for lung cancer screening scenarios ordered by QALYs

Strategy	Costs per 100,000 people (CNY, thousand)	Incremental cost per 100,000 people (CNY, thousand)		QALYs per 100,000 people	Incremental QALYs per 100,000 people		ICER (CNY/QALY)		iNMB
		**Vs the status quo strategy**	**Vs the strategy preceding it on the efficiency frontier**		**Vs the status quo strategy**	**Vs the strategy preceding it on the efficiency frontier**	**Vs the status quo strategy**	**Vs the strategy preceding it on the efficiency frontier**	
No screening	845,168	− 434,090	NA	1,150,366	− 3235	NA	Dominated	NA	− 3518
H1-MHnone-LMnone-Lnone	924,335	− 354,923	79,167	1,151,147	− 2453	781	Dominated	101,309	− 2411
H1-MHone-off-LMone-off-Lnone	947,457	− 331,801	102,289	1,151,200	− 2400	834	Dominated	122,594	− 2513
H1-MHone-off-LMone-off-Lone-off	958,602	− 320,655	113,434	1,151,202	− 2398	837	Dominated	135,567	− 2619
H1-MHone-off-LMnone-Lnone	937,823	− 341,434	92,656	1,151,247	− 2354	881	Dominated	105,176	− 2304
H1-MH3-LMone-off-Lnone	999,095	− 280,162	153,928	1,152,081	− 1520	1715	Dominated	89,762	− 891
H1-MH3-LMone-off-Lone-off	1,010,241	− 269,017	165,073	1,152,083	− 1518	1717	Dominated	96,129	− 997
H1-MH3-LMnone-Lnone^a^	989,462	− 289,795	144,295	1,152,127	− 1473	1761	Dominated	81,919	− 681
H1-MH2-LM3-Lnone	1,041,867	− 237,391	52,405	1,152,229	− 1372	101	Dominated	517,113	− 959
H1-MH2-LMone-off-Lnone	1,018,091	− 261,166	28,629	1,152,258	− 1343	131	Dominated	218,734	− 650
H1-MH2-LMone-off-Lone-off	1,029,237	− 250,021	39,774	1,152,260	− 1340	133	Dominated	298,492	− 755
H1-MH1-LM1-Lnone	1,123,224	− 156,033	133,762	1,152,277	− 1323	150	Dominated	891,217	− 1654
H1-MH2-LMnone-Lnone^a^	1,008,458	− 270,799	18,996	1,152,305	− 1296	177	Dominated	107,036	− 440
H1-MH1-LMone-off-Lnone	1,047,048	− 232,210	38,590	1,152,354	− 1247	49	Dominated	781,869	− 706
H1-MH1-LMone-off-Lone-off	1,058,193	− 221,064	49,735	1,152,356	− 1244	52	Dominated	961,581	− 812
H1-MH1-LM2-Lnone	1,090,559	− 188,698	82,101	1,152,357	− 1243	53	Dominated	1,553,900	− 1133
H1-MH1-LMnone-Lnone	1,037,415	− 241,843	28,957	1,152,401	− 1200	96	Dominated	301,814	− 497
H1-MH3-LM3-Lnone	1,039,447	− 239,810	30,989	1,152,411	− 1190	106	Dominated	291,936	− 492
H1-MH3-LM3-Lone-off	1,050,593	− 228,665	42,135	1,152,413	− 1187	109	Dominated	388,277	− 598
H1-MH2-LM3-Lone-off	1,069,589	− 209,669	61,131	1,152,591	− 1010	286	Dominated	213,752	− 357
H1-MH2-LM2-Lnone	1,078,179	− 201,079	69,721	1,152,621	− 979	317	Dominated	220,186	− 368
H1-MH2-LM2-Lone-off	1,089,324	− 189,933	80,866	1,152,624	− 977	319	Dominated	253,490	− 474
H1-MH1-LM1-Lone-off	1,150,946	− 128,312	142,488	1,152,639	− 961	335	Dominated	425,672	− 1052
H1-MH1-LM3-Lnone	1,087,400	− 191,858	78,942	1,152,684	− 916	380	Dominated	207,980	− 308
H1-MH1-LM3-Lone-off	1,098,545	− 180,712	90,087	1,152,687	− 914	382	Dominated	235,874	− 413
H1-MH1-LM2-Lone-off	1,118,281	− 160,977	109,823	1,152,720	− 881	415	Dominated	264,663	− 530
H1-MH3-LM3-L3^a^	1,114,292	− 164,966	105,834	1,153,247	− 354	942	Dominant	112,300	791
H1-MH2-LM3-L3^a^	1,133,288	− 145,970	18,996	1,153,425	− 176	177	Dominant	107,036	1032
H1-MH2-LM2-L3	1,153,023	− 126,234	38,731	1,153,458	− 143	33	Dominant	1,172,851	915
H1-MH1-LM1-L3	1,214,645	− 64,613	100,353	1,153,473	− 127	49	Dominant	2,058,604	337
H1-MH1-LM3-L3	1,162,244	− 117,013	47,952	1,153,520	− 80	96	Dominant	499,807	975
H1-MH1-LM2-L3	1,181,980	− 97,277	67,688	1,153,554	− 47	129	Dominant	524,855	858
H1-MH2-LM2-L2^a^	1,177,849	− 101,409	63,557	1,153,596	− 5	171	Dominant	371,625	1002
H1-MH1-LM1-L1 (status quo strategy)	1,279,258	NA	101,409	1,153,601	NA	5	NA	20,062,309	NA
H1-MH1-LM1-L2	1,239,470	− 39,787	61,622	1,153,611	11	16	Cost-saving	3,918,770	424
H1-MH1-LM2-L2^a^	1,206,805	− 72,452	28,957	1,153,692	91	96	Cost-saving	301,814	945

*QALY* quality-adjusted life year, *ICER* the incremental cost-effectiveness ratio, *iNMB* incremental net monetary benefit

^a^These strategies comprised the cost-effectiveness efficiency frontier

### Benefits and harms of screening

Compared with the status quo strategy, risk-based screening averted 37 ~ 597 fewer lung cancer deaths per 100,000 person and yielded 839 ~ 11,648 less life years per 100,000 people. However, risk-based screening strategies were more efficient than the status quo strategy, requiring 129 ~ 656 fewer screenings per lung cancer death avoided, and 0.5 ~ 28 fewer screenings per life-year gained. In addition, risk-based screening yields 5 ~ 31 fewer over-diagnosed cases per 100,000 people, 0.15 ~ 0.90 fewer radiation-related lung cancer cases per 100,000 people, and 1138 ~ 7218 fewer false-positive lung cancer cases per 100,000 people than the status quo strategy (Table 4).

**Table 4:** Benefits and harms of the screening scenarios

**Strategy**	**Number of LDCT screens (per 100,000 people)**	**Lung cancer deaths averted (per 100,000 people)**^†^	**Lung cancer mortality reduction (%)**	**Average number of screens per lung cancer death averted**	**Incremental life years gained (per 100,000 people)**^‡^	**Average number of screens per life years gained**	**Number of over-diagnosed lung cancers (per 100,000 people)**	**Number of radiation-related lung cancer (per 100,000 people)**	**Number of false-positive lung cancers (per 100,000 people)**
H1-MH1-LM1-L1 (status quo strategy)	1,071,810	770	35.6	1392	13,949	77	38	1.09	8373
H1-MH1-LM1-L2	926,440	733	33.9	1263	13,110	71	33	0.94	7235
H1-MH1-LM1-L3	848,420	698	32.3	1216	12,291	69	30	0.87	6624
H1-MH1-LM1-Lone-off	702,440	559	25.9	1257	9130	77	25	0.71	5488
H1-MH1-LM1-Lnone	677,630	552	25.5	1228	8960	76	24	0.70	5293
H1-MH1-LM2-L2^‡^	811,880	707	32.7	1148	12,599	64	30	0.86	6337
H1-MH1-LM2-L3	733,860	672	31.1	1093	11,779	62	28	0.79	5727
H1-MH1-LM2-Lone-off	587,880	533	24.6	1103	8608	68	22	0.63	4591
H1-MH1-LM2-Lnone	563,070	526	24.3	1071	8439	67	22	0.63	4396
H1-MH1-LM3-L3	673,650	648	30.0	1039	11,320	60	26	0.75	5256
H1-MH1-LM3-Lone-off	527,670	510	23.6	1035	8158	65	21	0.59	4119
H1-MH1-LM3-Lnone	502,860	502	23.2	1001	7989	63	20	0.59	3925
H1-MH1-LMone-off-Lone-off	426,400	434	20.1	981	6714	64	18	0.52	3329
H1-MH1-LMone-off-Lnone	401,590	427	19.8	940	6544	61	18	0.52	3135
H1-MH1-LMnone-Lnone	377,890	424	19.6	892	6474	58	18	0.51	2949
H1-MH2-LM2-L2^‡^	729,660	665	30.8	1097	11,893	61	26	0.75	5694
H1-MH2-LM2-L3	651,640	629	29.1	1035	11,074	59	24	0.68	5083
H1-MH2-LM2-Lone-off	505,670	491	22.7	1030	7903	64	18	0.52	3947
H1-MH2-LM2-Lnone	480,860	483	22.4	995	7733	62	18	0.51	3753
H1-MH2-LM3-L3^‡^	591,440	606	28.0	976	10,614	56	22	0.64	4612
H1-MH2-LM3-Lone-off	445,460	467	21.6	953	7453	60	17	0.48	3476
H1-MH2-LM3-Lnone	420,650	460	21.3	914	7284	58	16	0.47	3282
H1-MH2-LMone-off-Lone-off	344,180	392	18.1	877	6008	57	14	0.41	2685
H1-MH2-LMone-off-Lnone	319,380	385	17.8	830	5839	55	14	0.40	2491
H1-MH2-LMnone-Lnone^‡^	295,680	382	17.7	775	5769	51	14	0.40	2305
H1-MH3-LM3-L3^‡^	549,100	569	26.3	966	9999	55	20	0.58	4281
H1-MH3-LM3-Lone-off	403,120	430	19.9	938	6838	59	15	0.42	3145
H1-MH3-LM3-Lnone	378,320	423	19.5	895	6669	57	14	0.41	2951
H1-MH3-LMone-off-Lone-off	301,850	355	16.4	851	5393	56	12	0.35	2354
H1-MH3-LMone-off-Lnone	277,050	347	16.1	797	5224	53	12	0.34	2160
H1-MH3-LMnone-Lnone^‡^	253,350	344	15.9	736	5154	49	12	0.34	1975
H1-MHone-off-LMone-off-Lone-off	221,070	203	9.4	1089	2962	75	8	0.23	1727
H1-MHone-off-LMone-off-Lnone	196,260	196	9.1	1003	2693	73	8	0.22	1533
H1-MHone-off-LMnone-Lnone	172,560	192	8.9	897	2623	66	8	0.21	1347
H1-MHnone-LMnone-Lnone	148,000	173	8.0	854	2301	64	7	0.19	1155

^‡^These strategies comprised the cost-effectiveness efficiency frontier

^†^Number of lung cancer deaths per 100,000 people without screening: 2162

^‡^Life years per person without screening: 18.305

### One-way sensitivity analyses

**Fig. 3:**
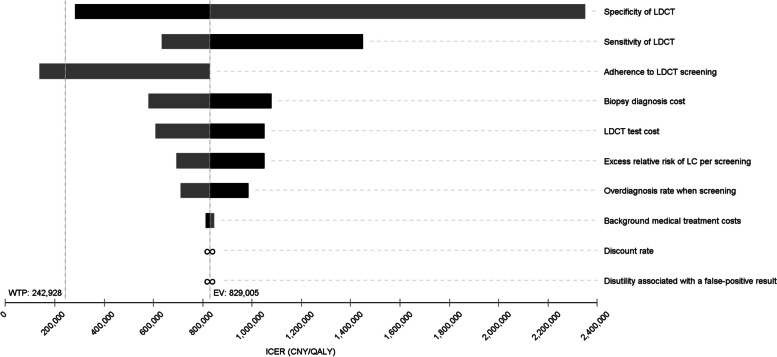
One-way sensitivity analyses of the ICER of H1-MH2-LM3-L3 vs status quo strategy (H1-MH1-LM1-L1) for lung cancer. The gray column shows the impact of decreasing the input parameters on the results. Similarly, the dark column shows the impact of increasing the input parameters on the results

**Fig. 4:**
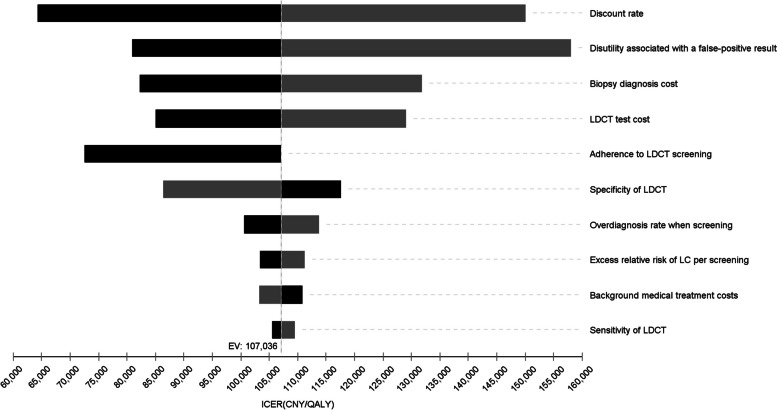
One-way sensitivity analyses of the ICER of H1-MH2-LM3-L3 vs the strategy preceding it on the efficiency frontier (H1-MH3-LM3-L3) for lung cancer. The gray column shows the impact of decreasing the input parameters on the results. Similarly, the dark column shows the impact of increasing the input parameters on the results

The one-way sensitivity analyses revealed that the primary outcomes were robust to changes in the value of most input parameters (Figs. 3 and 4, Additional file 1: Table S4 ~ Table S22). Compared with the status quo strategy, risk-based screening tended to be more cost-effective if the sensitivity of LDCT, adherence to LDCT screening, biopsy diagnosis cost, LDCT test cost, excess relative risk of lung cancer per screening, and overdiagnosis rate were increased, while this tended to be less cost-effective if the specificity of LDCT was increased. Particularly, H1-MH2-LM3-L3 would be dominated by the status quo strategy if adherence to LDCT screening was decreased to 0.72 (Fig. 3, Additional file 1: Table S20). In addition, when the upper bound for the specificity of LDCT (0.930) was set, H1-MH2-LM2-L2 was the most cost-effective with the largest iNMB (Additional file 1: Table S7). When biopsy diagnosis cost or disutility associated with a false-positive screen was decreased by 50%, or no discount was assumed, H1-MH1-LM2-L2 was most cost-effective with the largest iNMB (Additional file 1: Table S12, Table S18, Table S21).

If individuals quit smoking after being screened, more QALYs and fewer costs would be gained, and risk-based strategies tended to be more cost-effective compared with the status quo strategy (Additional file 1: Table S23). H1-MH2-LM3-L3 was still the most cost-effective strategy with the largest iNMB of CNY 1256 at the WTP threshold of CNY 242,928 per QALY gained.

### Probabilistic sensitivity analyses

The results of the probability sensitivity analyses are shown in Additional file 1: Fig. S1 ~ Fig. S13. At the WTP threshold of CNY 242,928 per QALY gained, H1-MH2-LM3-L3 had a high probability (approximately 95%) of being cost-effective compared with the strategy preceding it on the efficiency frontier and had a probability of above 50% of being cost-effective compared with the strategy following it on the efficiency frontier, regardless of whether individuals quit smoking or not. Therefore, H1-MH2-LM3-L3 was the optimal strategy at the WTP threshold of three times the 2021 GDP of China.

## Discussion

We found that risk-based screening strategies consistently reduced lung cancer deaths and gained life years more efficiently than the China 2021 guideline recommendation for heavy smokers, with fewer over-diagnosed, radiation-related, and false-positive lung cancer cases. Under the WTP threshold of three times the 2021 GDP of China, the guideline recommendation was dominant by nine risk-based screening strategies. The strategy of H1-MH2-LM3-L3, namely annual screening for individuals with a 5-year risk threshold of 1.70% or greater, biennial screening for individuals with a 5-year risk threshold of 1.03 ~ 1.69%, and triennial screening for individuals with a 5-year risk threshold of less than 1.03%, was the optimal strategy. These findings demonstrate that risk-stratified screening intervals are important to balance long-term benefit–harm trade-offs and improve the cost-effectiveness of lung cancer screening. To our knowledge, this is the first study to evaluate the long-term benefit–harm and cost-effectiveness of risk-based lung cancer screening strategies with different risk thresholds for screening eligibility and screening intervals using real-world data in China.

Notably, most risk-based strategies were not cost-effective compared with the current guideline recommendation, despite screening efficiency. For example, the strategy of H1-MH3-LMnone-Lnone (that is, annual screening for individuals with a 5-year risk threshold of 1.70% or greater; triennial screening for individuals with a 5-year risk threshold of 1.03 ~ 1.69%; and no screening for individuals with a 5-year risk threshold of less than 1.03%) was most efficient in preventing lung cancer death and increasing life years gained, but still dominant by the guideline recommendation under the WTP of three times the 2021 GDP of China. Thus, implementing the screening strategy without careful consideration of health outcomes and costs may not be recommended. In addition, the selection of the optimal screening strategy depends on the WTP. After evaluating the results in detail, we found that, if the WTP was lower, such as 1 times the 2021 GDP of China, H1-MH3-LMnone-Lnone would be the optimal strategy among those on the efficiency frontier. Therefore, in resource-limited areas of China, H1-MH3-LMnone-Lnone may be a better option.

Furthermore, when we assumed the maximum levels of LDCT specificity or assumed the minimum levels of biopsy diagnosis cost or disutility associated with a false-positive screen, lower screening intervals for individuals with a 5-year risk threshold of less than 1.70% would be more cost-effective. This finding suggested that the harm of false positives had a significant impact on the cost-effectiveness of risk-based screening. Thus, reducing the harm of false positives caused by LDCT screening is the key to improving the benefits and cost-effectiveness of lung cancer screening. Nevertheless, some risk-based screening strategies were still cost-effective compared with the status quo strategy.

Adherence rate has been demonstrated to be a key factor affecting the benefits of lung cancer screening [46]. Despite some studies suggesting that adherence to risk-based screening tends to be higher than that of annual screening for all individuals [47], we presumed the same adherence for the two strategies, to conservatively estimate the benefits of risk-based screening strategy. In the real world, the adherence rate to LDCT screening is generally low [48]. However, in this study, our objective was to estimate the screening outcomes for individuals who were willing to undergo screening. Therefore, we assumed a full adherence rate for the purpose of our analysis. We also performed sensitivity analyses to investigate the impact of imperfect adherence rates on the results. Our findings revealed that if the adherence rate dropped to 0.72, risk-based screening would no longer be considered cost-effective when compared to the guideline recommendation. There is one main reason accounting for this: we assumed that individuals had a certain probability of being diagnosed through standard clinical care when they did not receive LDCT screening. Under this assumption, with the reduced adherence rate, the gap in screening outcomes between risk-based screening and the guideline recommendation gradually decreased. Consistent with previous studies [49, 50], the combination of lung cancer screening and smoking cessation was associated with improved cost-effectiveness of the overall screening program more than LDCT screening alone. We further found that risk-based strategies would be also more cost-effective compared with the status quo strategy if individuals quit smoking after being screened. Therefore, the integration of adherence incentive and smoking cessation practices into lung cancer screening is recommended to maximize the cost-effectiveness of risk-based screening.

There have been no comparable studies estimating the benefit–harm and cost-effectiveness of risk-based lung cancer screening in China. The analytical approach was somewhat like that in a modeling study from the US [51], which compared cost-effectiveness among risk-based strategies and USPSTF recommendations using 1960 US birth cohort data. However, the risk-based strategies we made were enriched by adding screening intervals and thus arrived at different conclusions.

The findings from this study carry significant public health meaning. The risk-based screening strategy we developed has the potential to optimize the allocation of limited healthcare resources, by focusing screening efforts on individuals with a higher risk of developing lung cancer. This targeted approach ensures that screening is provided to those who are most likely to benefit, reducing unnecessary costs and potential harms associated with LDCT screening low-risk individuals. Risk-based screening also allows for a more equitable distribution of lung cancer screening services. By identifying individuals at higher risk, including those from disadvantaged populations, it ensures that those who are most in need of screening and early detection are not left behind. Moreover, risk-based screening provides an opportunity to raise public awareness about their lung cancer risk and the importance of early detection. It can help individuals to make informed decisions about their health and take proactive steps to reduce risk.

This study has several limitations. First, the relative risk model we used for lung cancer included a history of tuberculosis and emphysema, which require more complex medical examinations to be determined. Thus, acquiring additional information of such risk factors might be a barrier for the implementation of risk-based screening. Second, we did not consider the increase in risk of developing other cancers associated with exposure to screening radiation or the harms of incidental findings [52]. Third, we used a health system perspective and did not include productivity loss, the effect on the quality of life of caregivers, and physician and facility costs. Fourth, the choice of risk thresholds may have an impact on the results of benefit–harm and cost-effectiveness analyses. In the future, it is important to explore the possibility of implementing more personalized lung cancer screening approaches. This would involve identifying individualized, real-time eligibility criteria for lung cancer screening that take into account an individual’s specific risk factors and life expectancy. the data of the LDCT screening cohort were from the urban areas of Henan province, and the recommended strategy may not be optimal for other provinces and rural China, given potential differences in the sociodemographic risk factors, prevalence of lung cancer, and economical level.

## Conclusions

Risk-based screening strategies could reduce lung cancer deaths and increase life years gained more efficiently than China’s 2021 guideline recommendation for heavy smokers. Furthermore, risk-stratified screening intervals are important to balance long-term benefit–harm trade-offs and improve the cost-effectiveness of lung cancer screening. Integration of adherence incentive and smoking cessation practices into lung cancer screening is recommended to maximize the cost-effectiveness of risk-based screening.

### Supplementary Information

**Additional file 1: Table S1.** Risk-factors considered in the relative risk model for lung cancer. **Table S2.** Estimated incidence of lung cancer and mortality rates of non-lung cancer by sex and age (1/10^5). **Table S3.** Details of the evaluated LDCT screening strategies. **Table S4.** Cost-effectiveness estimates for lung cancer screening scenarios ordered by QALYs (Sensitivity of LDCT: 0.890). **Table S5.** Cost-effectiveness estimates for lung cancer screening scenarios ordered by QALYs (Sensitivity of LDCT: 1.000). **Table S6.** Cost-effectiveness estimates for lung cancer screening scenarios ordered by QALYs (Specificity of LDCT: 0.700). **Table S7.** Cost-effectiveness estimates for lung cancer screening scenarios ordered by QALYs (Specificity of LDCT: 0.930). **Table S8.** Cost-effectiveness estimates for lung cancer screening scenarios ordered by QALYs (overdiagnosis rate when screening: 0.0155). **Table S9.** Cost-effectiveness estimates for lung cancer screening scenarios ordered by QALYs (overdiagnosis rate when screening: 0.0465). **Table S10.** Cost-effectiveness estimates for lung cancer screening scenarios ordered by QALYs (Excess relative risk of LC per screening: 0.0003). **Table S11.** Cost-effectiveness estimates for lung cancer screening scenarios ordered by QALYs (Excess relative risk of LC per screening: 0.0019). **Table S12.** Cost-effectiveness estimates for lung cancer screening scenarios ordered by QALYs (Biopsy diagnosis cost: decreased by 50%). **Table S13.** Cost-effectiveness estimates for lung cancer screening scenarios ordered by QALYs (Biopsy diagnosis cost: increased by 50%). **Table S14.** Cost-effectiveness estimates for lung cancer screening scenarios ordered by QALYs (LDCT test cost: decreased by 50%). **Table S15.** Cost-effectiveness estimates for lung cancer screening scenarios ordered by QALYs (LDCT test cost: increased by 50%). **Table S16.** Cost-effectiveness estimates for lung cancer screening scenarios ordered by QALYs (Background medical treatment costs: decreased by 50%). **Table S17.** Cost-effectiveness estimates for lung cancer screening scenarios ordered by QALYs (Background medical treatment costs: increased by 50%). **Table S18.** Cost-effectiveness estimates for lung cancer screening scenarios ordered by QALYs (Disutility associated with a false positive screen: decreased by 50%). **Table S19.** Cost-effectiveness estimates for lung cancer screening scenarios ordered by QALYs (Disutility associated with a false positive screen: increased by 50%). **Table S20.** Cost-effectiveness estimates for lung cancer screening scenarios ordered by QALYs (Adherence: decreased by 50%). **Table S21.** Cost-effectiveness estimates for lung cancer screening scenarios ordered by QALYs (No discount). **Table S22.** Cost-effectiveness estimates for lung cancer screening scenarios ordered by QALYs (Discount: 8%). **Table S23.** Cost-effectiveness estimates for lung cancer screening scenarios ordered by QALYs (Quit smoking with being screened). **Fig. S1.** Cost-effectiveness acceptability curves of H1-MH3-LMnone-Lnone vs No screening. QALY, quality-adjusted life year; GDP, gross domestic product. **Fig. S2.** Cost-effectiveness acceptability curves of H1-MH2-LMnone-Lnone vs H1-MH3-LMnone-Lnone. QALY, quality-adjusted life year; GDP, gross domestic product. **Fig. S3.** Cost-effectiveness acceptability curves of H1-MH3-LM3-L3 vs H1-MH2-LMnone-Lnone. QALY, quality-adjusted life year; GDP, gross domestic product. **Fig. S4.** Cost-effectiveness acceptability curves of H1-MH2-LM3-L3 vs H1-MH3-LM3-L3. QALY, quality-adjusted life year; GDP, gross domestic product. **Fig. S5.** Cost-effectiveness acceptability curves of H1-MH2-LM2-L2 vs H1-MH2-LM3-L3. QALY, quality-adjusted life year; GDP, gross domestic product. **Fig. S6.** Cost-effectiveness acceptability curves of H1-MH1-LM2-L2 vs H1-MH2-LM2-L2. QALY, quality-adjusted life year; GDP, gross domestic product. **Fig. S7.** Cost-effectiveness acceptability curves of H1-MHone-off-LMone-off-Lone-off vs No screening if individuals quit smoking with being screened. QALY, quality-adjusted life year; GDP, gross domestic product. **Fig. S8.** Cost-effectiveness acceptability curves of H1-MH3-LMone-off-Lone-off vs H1-MHone-off-LMone-off-Lone-off if individuals quit smoking with being screened. QALY, quality-adjusted life year; GDP, gross domestic product. **Fig. S9.** Cost-effectiveness acceptability curves of H1-MH3-LM3-L3 vs H1-MH3-LMone-off-Lone-off if individuals quit smoking with being screened. QALY, quality-adjusted life year; GDP, gross domestic product. **Fig. S10.** Cost-effectiveness acceptability curves of H1-MH2-LM3-L3 vs H1-MH3-LM3-L3 if individuals quit smoking with being screened. QALY, quality-adjusted life year; GDP, gross domestic product. **Fig. S11.** Cost-effectiveness acceptability curves of H1-MH1-LM3-L3 vs H1-MH2-LM3-L3 if individuals quit smoking with being screened. QALY, quality-adjusted life year; GDP, gross domestic product. **Fig. S12.** Cost-effectiveness acceptability curves of H1-MH2-LM2-L2 vs H1-MH1-LM3-L3 if individuals quit smoking with being screened. QALY, quality-adjusted life year; GDP, gross domestic product. **Fig. S13.** Cost-effectiveness acceptability curves of H1-MH1-LM2-L2 vs H1-MH2-LM2-L2 if individuals quit smoking with being screened. QALY, quality-adjusted life year; GDP, gross domestic product.

**Additional file 2.** Overview of the sample survey on lung cancer care costs.

## Data Availability

The datasets used and/or analyzed during the current study are available from the corresponding author on reasonable request.

## Abbreviations

CanSPUC: Cancer screening program started in urban China
CNY: Chinese yuan
GDP: Gross domestic product
ICER: Incremental cost-effectiveness ratio
iNMB: Incremental net monetary benefit
LDCT: Low-dose computed tomography
NCCR: National Central Cancer Registry
NLST: National Lung Screening Trial
PAR: Population-attributable risk
QALY: Quality-adjusted life year
WTP: Willingness-to-pay
