# Supplementary material for: Risk-based lung cancer screening in heavy smokers: a benefit–harm and cost-effectiveness modeling study
Source: BMC Med. 2024 Feb 19;22:73. doi: 10.1186/s12916-024-03292-4 (PMC10875747; doi:10.1186/s12916-024-03292-4)
Supplement: Supplementary file 1 — Additional file 1: Table S1. Risk-factors considered in the relative risk model for lung cancer. Table S2. Estimated incidence of lung cancer and mortality rates of non-lung cancer by sex and age (1/10^5). Table S3. Details of the evaluated LDCT screening strategies. Table S4. Cost-effectiveness estimates for lung cancer screening scenarios ordered by QALYs (Sensitivity of LDCT: 0.890). Table S5. Cost-effectiveness estimates for lung cancer screening scenarios ordered by QALYs (Sensitivity of LDCT: 1.000). Table S6. Cost-effectiveness estimates for lung cancer screening scenarios ordered by QALYs (Specificity of LDCT: 0.700). Table S7. Cost-effectiveness estimates for lung cancer screening scenarios ordered by QALYs (Specificity of LDCT: 0.930). Table S8. Cost-effectiveness estimates for lung cancer screening scenarios ordered by QALYs (overdiagnosis rate when screening: 0.0155). Table S9. Cost-effectiveness estimates for lung cancer screening scenarios ordered by QALYs (overdiagnosis rate when screening: 0.0465). Table S10. Cost-effectiveness estimates for lung cancer screening scenarios ordered by QALYs (Excess relative risk of LC per screening: 0.0003). Table S11. Cost-effectiveness estimates for lung cancer screening scenarios ordered by QALYs (Excess relative risk of LC per screening: 0.0019). Table S12. Cost-effectiveness estimates for lung cancer screening scenarios ordered by QALYs (Biopsy diagnosis cost: decreased by 50%). Table S13. Cost-effectiveness estimates for lung cancer screening scenarios ordered by QALYs (Biopsy diagnosis cost: increased by 50%). Table S14. Cost-effectiveness estimates for lung cancer screening scenarios ordered by QALYs (LDCT test cost: decreased by 50%). Table S15. Cost-effectiveness estimates for lung cancer screening scenarios ordered by QALYs (LDCT test cost: increased by 50%). Table S16. Cost-effectiveness estimates for lung cancer screening scenarios ordered by QALYs (Background medical treatment costs: decreased by 50%). T [file 12916_2024_3292_MOESM1_ESM.pdf]

SUPPLEMENTAL MATERIAL—ADDITIONAL FILE 1

Risk-based lung cancer screening in heavy smokers: a benefit–harm and cost-effectiveness modelling study

Yin Liu[1], Huifang Xu [1], Lihong Lv[1], Xiaoyang Wang[1], Ruihua Kang [1], Xiaoli Guo[1], Hong Wang [1], Liyang Zheng[1], Hongwei Liu [1], Lanwei Guo [1], Qiong Chen [1], Shuzheng Liu [1], Youlin Qiao [1,2*], Shaokai Zhang [1*]

[1]Department of Cancer Epidemiology, The Affiliated Cancer Hospital of Zhengzhou University & Henan Cancer Hospital, Zhengzhou, 450008, China

[2]Center for Global Health, School of Population Medicine and Public Health, Chinese Academy of Medical Sciences and Peking Union Medical College, Beijing 100005, China

Corresponding Author:

Youlin Qiao, Email: qiaoy@cicams.ac.cn; Fax:(0371)-65587361;Tel: (0371)-65587361

Shaokai Zhang, Email: shaokaizhang@126.com; Fax:(0371)-65587361; Tel: (0371)-65587361

Table of Contents: Page

Supplemental Tables

Table S1 Risk-factors considered in the relative risk model for lung cancer…………5
Table S2 Estimated incidence of lung cancer and mortality rates of non-lung cancer by sex and age (1/10^5) 6
Table S3 Details of the evaluated LDCT screening strategies 7
Table S4 Cost-effectiveness estimates for lung cancer screening scenarios ordered by QALYs (Sensitivity of LDCT: 0.890)..9
Table S5 Cost-effectiveness estimates for lung cancer screening scenarios ordered by QALYs (Sensitivity of LDCT: 1.000)..11
Table S6 Cost-effectiveness estimates for lung cancer screening scenarios ordered by QALYs (Specificity of LDCT: 0.700)..13
Table S7 Cost-effectiveness estimates for lung cancer screening scenarios ordered by QALYs (Specificity of LDCT: 0.930)..15
Table S8 Cost-effectiveness estimates for lung cancer screening scenarios ordered by QALYs (overdiagnosis rate when screening: 0.0155) 17
Table S9 Cost-effectiveness estimates for lung cancer screening scenarios ordered by QALYs (overdiagnosis rate when screening: 0.0465) 19
Table S10 Cost-effectiveness estimates for lung cancer screening scenarios ordered by QALYs (Excess relative risk of LC per screening: 0.0003) 21
Table S11 Cost-effectiveness estimates for lung cancer screening scenarios ordered by QALYs (Excess relative risk of LC per screening: 0.0019) 23
Table S12 Cost-effectiveness estimates for lung cancer screening scenarios ordered by QALYs (Biopsy diagnosis cost: decreased by 50%)...25
Table S13 Cost-effectiveness estimates for lung cancer screening scenarios ordered by QALYs (Biopsy diagnosis cost: increased by 50%) 27
Table S14 Cost-effectiveness estimates for lung cancer screening scenarios ordered by QALYs (LDCT test cost: decreased by 50%) 29
Table S15 Cost-effectiveness estimates for lung cancer screening scenarios ordered by QALYs (LDCT test cost: increased by 50%)...31
Table S16 Cost-effectiveness estimates for lung cancer screening scenarios ordered by QALYs (Background medical treatment costs: decreased by 50%) 33
Table S17 Cost-effectiveness estimates for lung cancer screening scenarios ordered by QALYs (Background medical treatment costs: increased by 50%)............................35
Table S18 Cost-effectiveness estimates for lung cancer screening scenarios ordered by QALYs (Disutility associated with a false positive screen: decreased by 50%)..........37
Table S19 Cost-effectiveness estimates for lung cancer screening scenarios ordered by

QALYs (Disutility associated with a false positive screen: increased by 50%)..........39
Table S20 Cost-effectiveness estimates for lung cancer screening scenarios ordered by QALYs (Adherence: decreased by 50%)..41
Table S21 Cost-effectiveness estimates for lung cancer screening scenarios ordered by QALYs (No discount)...43
Table S22 Cost-effectiveness estimates for lung cancer screening scenarios ordered by QALYs (Discount: 8%)...45
Table S23 Cost-effectiveness estimates for lung cancer screening scenarios ordered by QALYs (Quit smoking with being screened)..47

Supplemental Figures and Figure Legends

Fig. S1. Cost-effectiveness acceptability curves of H1-MH3-LMnone-Lnone *vs* No screening. QALY, quality-adjusted life year; GDP, gross domestic product. 49
Fig. S2. Cost-effectiveness acceptability curves of H1-MH2-LMnone-Lnone *vs* H1-MH3-LMnone-Lnone. QALY, quality-adjusted life year; GDP, gross domestic product. 50
Fig. S3. Cost-effectiveness acceptability curves of H1-MH3-LM3-L3 *vs* H1-MH2-LMnone-Lnone. QALY, quality-adjusted life year; GDP, gross domestic product. 50
Fig. S4. Cost-effectiveness acceptability curves of H1-MH2-LM3-L3 *vs* H1-MH3-LM3-L3. QALY, quality-adjusted life year; GDP, gross domestic product. 51
Fig. S5. Cost-effectiveness acceptability curves of H1-MH2-LM2-L2 *vs* H1-MH2-LM3-L3. QALY, quality-adjusted life year; GDP, gross domestic product. 51
Fig. S6. Cost-effectiveness acceptability curves of H1-MH1-LM2-L2 *vs* H1-MH2-LM2-L2. QALY, quality-adjusted life year; GDP, gross domestic product. 52
Fig. S7. Cost-effectiveness acceptability curves of H1-MHone-off-LMone-off-Lone-off *vs* No screening if individuals quit smoking with being screened. QALY, quality-adjusted life year; GDP, gross domestic product. 52
Fig. S8. Cost-effectiveness acceptability curves of H1-MH3-LMone-off-Lone-off *vs* H1-MHone-off-LMone-off-Lone-off if individuals quit smoking with being screened. QALY, quality-adjusted life year; GDP, gross domestic product...............................53
Fig. S9. Cost-effectiveness acceptability curves of H1-MH3-LM3-L3 *vs* H1-MH3-LMone-off-Lone-off if individuals quit smoking with being screened. QALY, quality-adjusted life year; GDP, gross domestic product. 54
Fig. S10. Cost-effectiveness acceptability curves of H1-MH2-LM3-L3 *vs* H1-MH3-LM3-L3 if individuals quit smoking with being screened. QALY, quality-adjusted life year; GDP, gross domestic product. 54
Fig. S11. Cost-effectiveness acceptability curves of H1-MH1-LM3-L3 *vs* H1-MH2-LM3-L3 if individuals quit smoking with being screened. QALY, quality-adjusted life year; GDP, gross domestic product. 55

Fig. S12. Cost-effectiveness acceptability curves of H1-MH2-LM2-L2 *vs* H1-MH1-LM3-L3 if individuals quit smoking with being screened. QALY, quality-adjusted life year; GDP, gross domestic product. 55
Fig. S13. Cost-effectiveness acceptability curves of H1-MH1-LM2-L2 *vs* H1-MH2-LM2-L2 if individuals quit smoking with being screened. QALY, quality-adjusted life year; GDP, gross domestic product. 56

Table S1 Risk-factors considered in the relative risk model for lung cancer

Risk factors	Hazard Ratio
**Age (years)**	
40-44	1.00 (Reference)
45-49	0.98 (0.42-2.26)
50-54	2.40 (1.15–5.00)
55-59	4.87 (2.41–9.84)
60-64	5.51 (2.75–11.04)
65-69	7.57 (3.79–15.16)
70-74	11.94 (5.71–24.96)
**Sex**	
Female	1.00 (Reference)
Male	1.72 (1.28–2.31)
**Smoking intensity (packyears)**	
0	1.00 (Reference)
1-29	1.38 (0.99–1.91)
30-49	1.64 (1.10–2.45)
≥50	1.95 (1.22–3.13)
**Self-reported history of tuberculosis**	
No	1.00 (Reference)
Yes	1.88 (1.03–3.41)
**Self-reported history of emphysema**	
No	1.00 (Reference)
Yes	2.09 (1.21–3.61)

Table S2 Estimated incidence of lung cancer and mortality rates of non-lung cancer by sex and age (1/10^5)

Age group	Male		Female	
	Incidence of lung cancer	Mortality of non-lung cancer	Incidence of lung cancer	Mortality of non-lung cancer
50-54	84.34	494.64	50.87	240.18
55-59	121.85	751.76	56.99	366.35
60-64	237.82	1,189.10	104.22	618.29
65-69	329.68	1,809.90	137.74	1,060.81
70-74	418.52	3,072.21	178.38	2,026.14

Table S3 Details of the evaluated LDCT screening strategies

Strategies	Name	Screening Interval			
		High-risk (H)	Medium-high-risk (MH)	Low-medium-risk (LM)	Low-risk (L)
S1	No screening	None	None	None	None
S2	Universal screening (H1-MH1-LM1-L1 (status quo strategy))	Annual	Annual	Annual	Annual
S3	H1-MH1-LM1-L2	Annual	Annual	Annual	Biennial
S4	H1-MH1-LM1-L3	Annual	Annual	Annual	Triennial
S5	H1-MH1-LM1-Lone-off	Annual	Annual	Annual	One-off
S6	H1-MH1-LM1-Lnone	Annual	Annual	Annual	None
S7	H1-MH1-LM2-L2	Annual	Annual	Biennial	Biennial
S8	H1-MH1-LM2-L3	Annual	Annual	Biennial	Triennial
S9	H1-MH1-LM2-Lone-off	Annual	Annual	Biennial	One-off
S10	H1-MH1-LM2-Lnone	Annual	Annual	Biennial	None
S11	H1-MH1-LM3-L3	Annual	Annual	Triennial	Triennial
S12	H1-MH1-LM3-Lone-off	Annual	Annual	Triennial	One-off
S13	H1-MH1-LM3-Lnone	Annual	Annual	Triennial	None
S14	H1-MH1-LMone-off-Lone-off	Annual	Annual	One-off	One-off
S15	H1-MH1-LMone-off-Lnone	Annual	Annual	One-off	None
S16	H1-MH1-LMnone-Lnone	Annual	Annual	None	None
S17	H1-MH2-LM2-L2	Annual	Biennial	Biennial	Biennial
S18	H1-MH2-LM2-L3	Annual	Biennial	Biennial	Triennial
S19	H1-MH2-LM2-Lone-off	Annual	Biennial	Biennial	One-off
S20	H1-MH2-LM2-Lnone	Annual	Biennial	Biennial	None

S21	H1-MH2-LM3-L3	Annual	Biennial	Triennial	Triennial
S22	H1-MH2-LM3-Lone-off	Annual	Biennial	Triennial	One-off
S23	H1-MH2-LM3-Lnone	Annual	Biennial	Triennial	None
S24	H1-MH2-LMone-off-Lone-off	Annual	Biennial	One-off	One-off
S25	H1-MH2-LMone-off-Lnone	Annual	Biennial	One-off	None
S26	H1-MH2-LMnone-Lnone	Annual	Biennial	None	None
S27	H1-MH3-LM3-L3	Annual	Triennial	Triennial	Triennial
S28	H1-MH3-LM3-Lone-off	Annual	Triennial	Triennial	One-off
S29	H1-MH3-LM3-Lnone	Annual	Triennial	Triennial	None
S30	H1-MH3-LMone-off-Lone-off	Annual	Triennial	One-off	One-off
S31	H1-MH3-LMone-off-Lnone	Annual	Triennial	One-off	None
S32	H1-MH3-LMnone-Lnone	Annual	Triennial	None	None
S33	H1-MHone-off-LMone-off-Lone-off	Annual	One-off	One-off	One-off
S34	H1-MHone-off-LMone-off-Lnone	Annual	One-off	One-off	None
S35	H1-MHone-off-LMnone-Lnone	Annual	One-off	None	None
S36	H1-MHnone-LMnone-Lnone	Annual	None	None	None

High-risk (H): individuals with a 5-year risk threshold of 1.70% or greater

Medium-high-risk (MH): individuals with a 5-year risk threshold of 1.03%~1.69%

Low-medium-risk (LM): individuals with a 5-year risk threshold of 0.49%~1.02%

Low-risk (L): individuals with a 5-year risk threshold of <0.49%

One-way sensitivity analyses

Results of one-way sensitivity analyses were shown as TableS4~TableS23.

Table S4 Cost-effectiveness estimates for lung cancer screening scenarios ordered by QALYs (Sensitivity of LDCT: 0.890)

Strategy	Costs per 100,000 people (CNY, thousand)	Incremental cost per 100,000 people (CNY, thousand)		QALYs per 100,000 people	Incremental QALYs per 100,000 people		ICER (CNY/QALY)		iNMB
		Vs the status quo strategy	Vs the strategy preceding it on the efficiency frontier		Vs the status quo strategy	Vs the strategy preceding it on the efficiency frontier	Vs the status quo strategy	Vs the strategy preceding it on the efficiency frontier	
No screening	842,816	-432,378	NA	1,150,319	-3,215	NA	Dominated	NA	-3,485
H1-MHnone-LMnone-Lnone	921,534	-353,660	78,718	1,151,094	-2,440	775	Dominated	101,565	-2,390
H1-MHone-off-LMone-off-Lnone	944,442	-330,752	101,626	1,151,141	-2,392	822	Dominated	123,611	-2,504
H1-MHone-off-LMone-off-Lone-off	955,508	-319,685	112,693	1,151,142	-2,392	822	Dominated	137,049	-2,615
H1-MHone-off-LMnone-Lnone	934,843	-340,351	92,027	1,151,189	-2,345	870	Dominated	105,818	-2,293
H1-MH3-LMone-off-Lnone	995,091	-280,103	152,275	1,151,996	-1,537	1,677	Dominated	90,795	-934
H1-MH3-LMone-off-Lone-off	1,006,158	-269,036	163,342	1,151,997	-1,537	1,677	Dominated	97,386	-1,044
H1-MH3-LMnone-Lnone‡	985,492	-289,702	142,676	1,152,044	-1,490	1,725	Dominated	82,727	-723
H1-MH2-LM3-Lnone	1,037,993	-237,201	52,501	1,152,146	-1,388	102	Dominated	512,567	-999
H1-MH2-LMone-off-Lnone	1,014,103	-261,091	28,611	1,152,177	-1,357	133	Dominated	215,604	-686
H1-MH2-LMone-off-Lone-off	1,025,169	-250,025	39,677	1,152,177	-1,357	133	Dominated	298,707	-797

H1-MH2-LMnone-Lnone‡	1,004,503	-270,690	19,011	1,152,224	-1,310	180	Dominated	105,486	-475
H1-MH1-LM1-Lnone	1,120,220	-154,974	115,716	1,152,227	-1,307	183	Dominated	630,857	-1,624
H1-MH1-LMone-off-Lnone	1,043,663	-231,531	39,159	1,152,294	-1,240	66	Dominated	590,271	-697
H1-MH1-LMone-off-Lone-off	1,054,729	-220,465	50,226	1,152,294	-1,240	66	Dominated	755,604	-808
H1-MH1-LM2-Lnone	1,087,264	-187,930	82,761	1,152,297	-1,237	70	Dominated	1,187,207	-1,125
H1-MH3-LM3-Lnone	1,034,945	-240,248	30,442	1,152,313	-1,220	86	Dominated	353,688	-562
H1-MH3-LM3-Lone-off	1,046,012	-229,182	41,509	1,152,314	-1,220	86	Dominated	481,537	-673
H1-MH1-LMnone-Lnone	1,034,063	-241,130	29,560	1,152,341	-1,193	114	Dominated	259,600	-486
H1-MH2-LM3-Lone-off	1,065,023	-210,170	60,520	1,152,494	-1,040	266	Dominated	227,155	-425
H1-MH2-LM2-Lnone	1,073,668	-201,526	69,164	1,152,527	-1,007	300	Dominated	230,595	-430
H1-MH2-LM2-Lone-off	1,084,734	-190,460	80,231	1,152,527	-1,006	300	Dominated	267,375	-540
H1-MH1-LM1-Lone-off	1,147,250	-127,944	142,746	1,152,575	-959	347	Dominated	410,869	-1,051
H1-MH1-LM3-Lnone	1,083,517	-191,677	79,013	1,152,611	-923	383	Dominated	206,105	-326
H1-MH1-LM3-Lone-off	1,094,583	-180,610	90,080	1,152,611	-923	383	Dominated	234,892	-436
H1-MH1-LM2-Lone-off	1,114,294	-160,900	109,791	1,152,645	-889	417	Dominated	263,201	-552
H1-MH3-LM3-L3‡	1,108,713	-166,480	104,210	1,153,121	-413	894	Dominant	116,631	661
H1-MH2-LM3-L3‡	1,127,725	-147,469	19,011	1,153,301	-233	180	Dominant	105,486	909
H1-MH2-LM2-L3	1,147,436	-127,758	19,711	1,153,335	-199	34	Dominant	585,909	794
H1-MH1-LM1-L3	1,209,951	-65,242	82,226	1,153,382	-152	81	Dominant	1,015,155	284
H1-MH1-LM3-L3	1,157,285	-117,909	29,560	1,153,418	-116	117	Dominant	252,503	898
H1-MH1-LM2-L3	1,176,996	-98,198	49,271	1,153,452	-82	151	Dominant	326,926	783
H1-MH2-LM2-L2‡	1,172,303	-102,891	44,578	1,153,476	-58	175	Dominant	254,433	889
H1-MH1-LM1-L2	1,234,819	-40,375	62,516	1,153,524	-10	47	Dominant	1,320,081	379
H1-MH1-LM1-L1 (status quo strategy)	1,275,194	NA	102,891	1,153,534	NA	58	NA	1,786,084	NA
H1-MH1-LM2-L2‡	1,201,863	-73,331	29,560	1,153,593	59	117	Cost-saving	252,503	878

‡ These strategies comprised the cost-effectiveness efficiency frontier. QALY, quality-adjusted life year; ICER, the incremental cost-effectiveness ratio; iNMB, incremental net monetary benefit

Table S5 Cost-effectiveness estimates for lung cancer screening scenarios ordered by QALYs (Sensitivity of LDCT: 1.000)

Strategy	Costs per 100,000 people (CNY, thousand)	Incremental cost per 100,000 people (CNY, thousand)		QALYs per 100,000 people	Incremental QALYs per 100,000 people		ICER (CNY/QALY)		iNMB
		Vs the status quo strategy	Vs the strategy preceding it on the efficiency frontier		Vs the status quo strategy	Vs the strategy preceding it on the efficiency frontier	Vs the status quo strategy	Vs the strategy preceding it on the efficiency frontier	
No screening	848,292	-436,200	NA	1,150,428	-3,258	NA	Dominated	NA	-3,553
H1-MHnone-LMnone-Lnone	928,016	-356,477	79,723	1,151,217	-2,469	789	Dominated	101,037	-2,434
H1-MHone-off-LMone-off-Lnone	951,423	-333,069	103,130	1,151,278	-2,408	850	Dominated	121,360	-2,520
H1-MHone-off-LMone-off-Lone-off	962,674	-321,818	114,382	1,151,283	-2,403	855	Dominated	133,757	-2,620
H1-MHone-off-LMnone-Lnone	941,745	-342,747	93,452	1,151,323	-2,363	895	Dominated	104,402	-2,313
H1-MH3-LMone-off-Lnone	1,004,365	-280,128	156,072	1,152,192	-1,494	1,764	Dominated	88,480	-829
H1-MH3-LMone-off-Lone-off	1,015,616	-268,876	167,323	1,152,197	-1,489	1,769	Dominated	94,572	-928
H1-MH3-LMnone-Lnone‡	994,686	-289,806	146,394	1,152,237	-1,449	1,809	Dominated	80,914	-622
H1-MH2-LM3-Lnone	1,046,936	-237,556	52,249	1,152,336	-1,350	99	Dominated	527,630	-904
H1-MH1-LM1-Lnone	1,127,097	-157,395	132,411	1,152,341	-1,345	104	Dominated	1,271,061	-1,693
H1-MH2-LMone-off-Lnone	1,023,317	-261,175	28,631	1,152,365	-1,321	128	Dominated	224,032	-598
H1-MH2-LMone-off-Lone-off	1,034,568	-249,924	39,882	1,152,370	-1,316	133	Dominated	299,507	-697
H1-MH2-LMnone-Lnone‡	1,013,639	-270,853	18,952	1,152,410	-1,276	173	Dominated	109,473	-391
H1-MH1-LMone-off-Lnone	1,051,452	-233,040	37,814	1,152,432	-1,254	22	Dominated	1,698,535	-715
H1-MH1-LM2-Lnone	1,094,829	-189,663	81,190	1,152,436	-1,251	25	Dominated	3,201,234	-1,141
H1-MH1-LMone-off-Lone-off	1,062,704	-221,789	49,065	1,152,438	-1,248	28	Dominated	1,776,159	-814

H1-MH1-LMnone-Lnone	1,041,774	-242,718	28,135	1,152,478	-1,208	68	Dominated	416,264	-508
H1-MH3-LM3-Lnone	1,045,372	-239,120	31,733	1,152,539	-1,147	129	Dominated	245,651	-394
H1-MH3-LM3-Lone-off	1,056,623	-227,869	42,985	1,152,545	-1,141	135	Dominated	319,488	-494
H1-MH2-LM3-Lone-off	1,075,576	-208,916	61,937	1,152,718	-968	308	Dominated	201,312	-263
H1-MH1-LM1-Lone-off	1,155,738	-128,755	142,099	1,152,723	-963	313	Dominated	454,260	-1,052
H1-MH2-LM2-Lnone	1,084,082	-200,410	70,444	1,152,744	-942	334	Dominated	210,798	-284
H1-MH2-LM2-Lone-off	1,095,334	-189,159	81,695	1,152,750	-936	340	Dominated	240,607	-383
H1-MH1-LM3-Lnone	1,092,460	-192,032	78,821	1,152,780	-906	370	Dominated	213,090	-281
H1-MH1-LM3-Lone-off	1,103,711	-180,781	90,073	1,152,785	-901	375	Dominated	240,029	-380
H1-MH1-LM2-Lone-off	1,123,469	-161,023	109,830	1,152,817	-869	407	Dominated	269,769	-500
H1-MH3-LM3-L3‡	1,121,636	-162,857	107,997	1,153,414	-272	1,004	Dominant	107,614	967
H1-MH2-LM3-L3‡	1,140,588	-143,904	18,952	1,153,587	-99	173	Dominant	109,473	1,198
H1-MH1-LM1-L3	1,220,750	-63,742	80,162	1,153,592	-94	5	Dominant	15,574,336	409
H1-MH2-LM2-L3	1,160,346	-124,146	19,758	1,153,619	-67	32	Dominant	619,956	1,078
H1-MH1-LM3-L3	1,168,723	-115,769	28,135	1,153,655	-32	68	Dominant	416,264	1,081
H1-MH1-LM1-L1 (status quo strategy)	1,284,492	NA	143,904	1,153,686	NA	99	NA	1,450,524	NA
H1-MH1-LM2-L3	1,188,481	-96,011	47,893	1,153,686	0	99	Cost-saving	481,532	961
H1-MH1-LM1-L2	1,245,498	-38,994	104,910	1,153,725	39	138	Cost-saving	761,590	484
H1-MH2-LM2-L2‡	1,185,094	-99,398	44,506	1,153,751	65	164	Cost-saving	270,595	1,153
H1-MH1-LM2-L2‡	1,213,229	-71,263	28,135	1,153,819	133	68	Cost-saving	416,264	1,035

‡ These strategies comprised the cost-effectiveness efficiency frontier. QALY, quality-adjusted life year; ICER, the incremental cost-effectiveness ratio; iNMB, incremental net monetary benefit

Table S6 Cost-effectiveness estimates for lung cancer screening scenarios ordered by QALYs (Specificity of LDCT: 0.700)

Strategy	Costs per 100,000 people (CNY, thousand)	Incremental cost per 100,000 people (CNY, thousand)		QALYs per 100,000 people	Incremental QALYs per 100,000 people		ICER (CNY/QALY)		iNMB
		Vs the status quo strategy	Vs the strategy preceding it on the efficiency frontier		Vs the status quo strategy	Vs the strategy preceding it on the efficiency frontier	Vs the status quo strategy	Vs the strategy preceding it on the efficiency frontier	
No screening	845,168	-437,290	NA	1,150,366	-3,021	NA	Dominated	NA	-2,966
H1-MHnone-LMnone-Lnone	924,535	-357,923	79,368	1,151,127	-2,260	761	Dominated	104,255	-1,910
H1-MHone-off-LMone-off-Lone-off	961,287	-321,171	116,119	1,151,150	-2,237	784	Dominated	148,062	-2,222
H1-MHone-off-LMone-off-Lnone	949,285	-333,173	104,117	1,151,160	-2,227	794	Dominated	131,074	-2,077
H1-MHone-off-LMnone-Lnone	938,808	-343,650	93,641	1,151,225	-2,162	859	Dominated	108,970	-1,814
H1-MH3-LMone-off-Lone-off	1,014,279	-268,179	169,111	1,152,041	-1,346	1,675	Dominated	100,953	-587
H1-MH3-LMone-off-Lnone	1,002,276	-280,182	157,109	1,152,051	-1,336	1,685	Dominated	93,227	-443
H1-MH3-LMnone-Lnone‡	991,800	-290,658	146,632	1,152,116	-1,271	1,750	Dominated	83,780	-180
H1-MH1-LM1-Lnone	1,125,246	-157,212	133,446	1,152,140	-1,247	24	Dominated	5,595,568	-1,457
H1-MH2-LM3-Lnone	1,047,392	-235,066	55,592	1,152,158	-1,229	42	Dominated	1,329,142	-634
H1-MH2-LMone-off-Lone-off	1,033,535	-248,923	41,735	1,152,205	-1,182	89	Dominated	470,865	-382
H1-MH2-LMone-off-Lnone	1,021,533	-260,925	29,733	1,152,215	-1,172	99	Dominated	301,213	-238
H1-MH1-LM2-Lnone	1,094,560	-187,899	102,760	1,152,244	-1,142	128	Dominated	799,843	-896
H1-MH2-LMnone-Lnone‡	1,011,056	-271,402	19,256	1,152,280	-1,107	164	Dominated	117,636	25
H1-MH1-LMone-off-Lone-off	1,059,964	-222,494	48,907	1,152,287	-1,099	8	Dominated	6,280,833	-445
H1-MH1-LMone-off-Lnone	1,047,961	-234,497	36,905	1,152,298	-1,089	18	Dominated	2,066,134	-301

H1-MH3-LM3-Lone-off	1,056,714	-225,744	45,658	1,152,344	-1,043	64	Dominated	712,155	-276
H1-MH3-LM3-Lnone	1,044,712	-237,746	33,656	1,152,354	-1,033	74	Dominated	453,656	-131
H1-MH1-LMnone-Lnone	1,037,485	-244,973	26,429	1,152,363	-1,024	83	Dominated	319,006	-38
H1-MH1-LM1-Lone-off	1,153,825	-128,634	142,768	1,152,489	-897	210	Dominated	680,399	-893
H1-MH2-LM3-Lone-off	1,075,971	-206,487	64,915	1,152,507	-879	228	Dominated	284,954	-71
H1-MH2-LM2-Lone-off	1,096,710	-185,749	85,653	1,152,511	-875	232	Dominated	369,817	-269
H1-MH2-LM2-Lnone	1,084,707	-197,751	73,651	1,152,521	-865	242	Dominated	304,739	-124
H1-MH1-LM3-Lone-off	1,102,399	-180,059	91,343	1,152,590	-796	311	Dominated	294,035	-134
H1-MH1-LM2-Lone-off	1,123,138	-159,320	112,082	1,152,594	-792	314	Dominated	356,430	-332
H1-MH1-LM3-Lnone	1,090,397	-192,061	79,341	1,152,600	-786	321	Dominant	247,376	11
H1-MH3-LM3-L3‡	1,122,854	-159,604	111,797	1,153,163	-223	884	Dominant	126,538	1,053
H1-MH1-LM1-L3	1,219,964	-62,494	97,110	1,153,309	-78	146	Dominant	666,427	436
H1-MH2-LM3-L3‡	1,142,110	-140,348	19,256	1,153,327	-60	164	Dominant	117,636	1,258
H1-MH2-LM2-L3	1,162,849	-119,609	20,739	1,153,331	-56	4	Dominant	5,453,838	1,060
H1-MH1-LM1-L1 (status quo strategy)	1,282,458	NA	140,348	1,153,387	NA	60	NA	2,349,321	NA
H1-MH1-LM3-L3	1,168,539	-113,919	26,429	1,153,410	23	83	Cost-saving	319,006	1,195
H1-MH1-LM2-L3	1,189,277	-93,181	47,167	1,153,414	27	87	Cost-saving	544,348	997
H1-MH1-LM1-L2	1,245,611	-36,847	103,501	1,153,421	35	95	Cost-saving	1,095,233	453
H1-MH2-LM2-L2‡	1,188,496	-93,962	46,386	1,153,443	57	116	Cost-saving	398,915	1,077
H1-MH1-LM2-L2‡	1,214,925	-67,533	26,429	1,153,526	139	83	Cost-saving	319,006	1,014

‡ These strategies comprised the cost-effectiveness efficiency frontier. QALY, quality-adjusted life year; ICER, the incremental cost-effectiveness ratio; iNMB, incremental net monetary benefit

Table S7 Cost-effectiveness estimates for lung cancer screening scenarios ordered by QALYs (Specificity of LDCT: 0.930)

Strategy	Costs per 100,000 people (CNY, thousand)	Incremental cost per 100,000 people (CNY, thousand)		QALYs per 100,000 people	Incremental QALYs per 100,000 people		ICER (CNY/QALY)		iNMB
		Vs the status quo strategy	Vs the strategy preceding it on the efficiency frontier		Vs the status quo strategy	Vs the strategy preceding it on the efficiency frontier	Vs the status quo strategy	Vs the strategy preceding it on the efficiency frontier	
No screening	845,168	-424,530	NA	1,150,366	-3,879	NA	Dominated	NA	-5,179
H1-MHnone-LMnone-Lnone	923,751	-345,946	78,584	1,151,207	-3,039	841	Dominated	93,456	-3,922
H1-MHone-off-LMnone-Lnone	935,245	-334,452	90,078	1,151,310	-2,935	944	Dominated	95,408	-3,786
H1-MHone-off-LMone-off-Lnone	942,735	-326,963	97,567	1,151,310	-2,935	944	Dominated	103,320	-3,861
H1-MHone-off-LMone-off-Lone-off	951,702	-317,996	106,534	1,151,344	-2,901	978	Dominated	108,895	-3,868
H1-MH3-LMnone-Lnone‡	983,451	-286,247	138,283	1,152,164	-2,081	1,798	Dominated	76,902	-2,194
H1-MH3-LMone-off-Lnone	990,940	-278,757	7,489	1,152,164	-2,081	0	Dominated	39,820,573	-2,268
H1-MH3-LMone-off-Lone-off	999,907	-269,790	16,457	1,152,198	-2,047	34	Dominated	481,393	-2,275
H1-MH2-LMnone-Lnone‡	1,001,788	-267,909	18,338	1,152,376	-1,869	212	Dominated	86,305	-1,861
H1-MH2-LMone-off-Lnone	1,009,278	-260,420	7,489	1,152,377	-1,869	1	Dominated	39,820,573	-1,935
H1-MH2-LMone-off-Lone-off	1,018,245	-251,453	16,457	1,152,411	-1,835	34	Dominated	481,393	-1,942
H1-MH2-LM3-Lnone	1,027,759	-241,939	25,971	1,152,417	-1,829	40	Dominated	647,337	-2,023
H1-MH1-LMnone-Lnone	1,037,266	-232,432	35,477	1,152,513	-1,732	137	Dominated	259,084	-1,883
H1-MH1-LMone-off-Lnone	1,044,755	-224,943	42,967	1,152,514	-1,732	137	Dominated	313,347	-1,957
H1-MH1-LMone-off-Lone-off	1,053,722	-215,975	51,934	1,152,548	-1,698	171	Dominated	303,496	-1,964
H1-MH3-LM3-Lnone	1,025,998	-243,700	24,209	1,152,564	-1,681	187	Dominated	129,188	-1,648

H1-MH3-LM3-Lone-off	1,034,965	-234,733	33,176	1,152,598	-1,647	221	Dominated	149,854	-1,655
H1-MH1-LM2-Lnone	1,080,426	-189,271	78,638	1,152,661	-1,585	284	Dominated	276,612	-1,956
H1-MH1-LM1-Lnone	1,117,199	-152,498	115,411	1,152,690	-1,555	314	Dominated	367,896	-2,253
H1-MH2-LM3-Lone-off	1,053,302	-216,395	51,514	1,152,810	-1,435	434	Dominated	118,732	-1,322
H1-MH2-LM2-Lnone	1,061,525	-208,172	59,737	1,152,884	-1,362	507	Dominated	117,799	-1,226
H1-MH1-LM3-Lnone	1,079,812	-189,885	78,024	1,152,913	-1,332	537	Dominated	145,349	-1,337
H1-MH2-LM2-Lone-off	1,070,492	-199,205	68,704	1,152,918	-1,328	541	Dominated	126,970	-1,233
H1-MH1-LM3-Lone-off	1,088,780	-180,918	86,991	1,152,947	-1,298	571	Dominated	152,402	-1,344
H1-MH1-LM2-Lone-off	1,105,970	-163,728	104,181	1,153,054	-1,191	678	Dominated	153,651	-1,255
H1-MH1-LM1-Lone-off	1,142,742	-126,955	140,954	1,153,084	-1,161	707	Dominated	199,241	-1,552
H1-MH3-LM3-L3‡	1,092,468	-177,229	90,680	1,153,469	-777	1,092	Dominated	83,030	-114
H1-MH2-LM3-L3‡	1,110,806	-158,891	18,338	1,153,681	-564	212	Dominant	86,305	218
H1-MH2-LM2-L3	1,127,996	-141,702	17,190	1,153,788	-457	107	Dominant	160,299	307
H1-MH1-LM3-L3	1,146,283	-123,414	35,477	1,153,818	-427	137	Dominant	259,084	196
H1-MH1-LM2-L3	1,163,473	-106,224	52,667	1,153,925	-320	244	Dominant	215,699	285
H1-MH1-LM1-L3	1,200,246	-69,451	89,440	1,153,955	-291	274	Dominant	326,918	-11
H1-MH2-LM2-L2‡	1,150,738	-118,960	39,932	1,153,991	-254	310	Dominant	128,823	572
H1-MH1-LM2-L2‡	1,186,215	-83,483	35,477	1,154,128	-117	137	Dominant	259,084	550
H1-MH1-LM1-L2	1,222,988	-46,710	72,250	1,154,157	-88	29	Dominant	2,456,154	254
H1-MH1-LM1-L1‡ (status quo strategy)	1,269,698	NA	118,960	1,154,245	NA	117	NA	1,014,312	NA

‡ These strategies comprised the cost-effectiveness efficiency frontier. QALY, quality-adjusted life year; ICER, the incremental cost-effectiveness ratio; iNMB, incremental net monetary benefit

Table S8 Cost-effectiveness estimates for lung cancer screening scenarios ordered by QALYs (overdiagnosis rate when screening: 0.0155)

Strategy	Costs per 100,000 people (CNY, thousand)	Incremental cost per 100,000 people (CNY, thousand)		QALYs per 100,000 people	Incremental QALYs per 100,000 people		ICER (CNY/QALY)		iNMB
		Vs the status quo strategy	Vs the strategy preceding it on the efficiency frontier		Vs the status quo strategy	Vs the strategy preceding it on the efficiency frontier	Vs the status quo strategy	Vs the strategy preceding it on the efficiency frontier	
No screening	846,762	-423,554	NA	1,150,101	-3,336	NA	Dominated	NA	-3,870
H1-MHnone-LMnone-Lnone	923,687	-346,629	76,925	1,150,904	-2,534	802	Dominated	95,875	-2,690
H1-MHone-off-LMone-off-Lnone	946,391	-323,925	99,629	1,150,961	-2,477	859	Dominated	115,954	-2,779
H1-MHone-off-LMone-off-Lone-off	957,368	-312,947	110,607	1,150,965	-2,473	863	Dominated	128,136	-2,879
H1-MHone-off-LMnone-Lnone	936,826	-333,489	90,065	1,151,006	-2,431	905	Dominated	99,505	-2,571
H1-MH3-LMone-off-Lnone	997,056	-273,260	150,294	1,151,859	-1,579	1,758	Dominated	85,504	-1,102
H1-MH3-LMone-off-Lone-off	1,008,034	-262,282	161,272	1,151,863	-1,575	1,762	Dominated	91,542	-1,203
H1-MH3-LMnone-Lnone‡	987,491	-282,824	140,730	1,151,905	-1,533	1,804	Dominated	78,025	-895
H1-MH2-LM3-Lnone	1,038,431	-231,885	50,939	1,152,016	-1,422	111	Dominated	459,000	-1,135
H1-MH2-LMone-off-Lnone	1,015,417	-254,898	27,926	1,152,042	-1,396	137	Dominated	204,528	-843
H1-MH2-LMone-off-Lone-off	1,026,395	-243,920	38,904	1,152,046	-1,392	141	Dominated	276,849	-943
H1-MH1-LM1-Lnone	1,117,258	-153,058	129,766	1,152,077	-1,361	172	Dominated	755,683	-1,776
H1-MH2-LMnone-Lnone‡	1,005,853	-264,463	18,362	1,152,087	-1,350	182	Dominated	100,639	-636
H1-MH1-LMone-off-Lnone	1,042,971	-227,345	37,118	1,152,143	-1,295	55	Dominated	669,579	-872
H1-MH1-LMone-off-Lone-off	1,053,949	-216,367	48,096	1,152,147	-1,291	59	Dominated	809,422	-972
H1-MH1-LM2-Lnone	1,085,346	-184,970	79,493	1,152,153	-1,284	66	Dominated	1,203,867	-1,270

H1-MH1-LMnone-Lnone	1,033,407	-236,909	27,553	1,152,189	-1,249	101	Dominated	271,870	-665
H1-MH3-LM3-Lnone	1,036,907	-233,408	31,054	1,152,199	-1,239	112	Dominated	278,445	-675
H1-MH3-LM3-Lone-off	1,047,885	-222,431	42,032	1,152,203	-1,235	116	Dominated	363,875	-775
H1-MH2-LM3-Lone-off	1,066,247	-204,069	60,394	1,152,385	-1,052	298	Dominated	202,688	-516
H1-MH2-LM2-Lnone	1,074,631	-195,685	68,777	1,152,418	-1,020	330	Dominated	208,331	-521
H1-MH2-LM2-Lone-off	1,085,608	-184,707	79,755	1,152,422	-1,016	334	Dominated	238,702	-622
H1-MH1-LM1-Lone-off	1,145,074	-125,242	139,221	1,152,446	-992	359	Dominated	388,119	-1,156
H1-MH1-LM3-Lnone	1,082,822	-187,493	76,969	1,152,483	-955	395	Dominated	194,698	-445
H1-MH1-LM3-Lone-off	1,093,800	-176,515	87,947	1,152,487	-951	399	Dominated	220,247	-545
H1-MH1-LM2-Lone-off	1,113,162	-157,154	107,309	1,152,523	-915	435	Dominated	246,422	-651
H1-MH3-LM3-L3‡	1,110,528	-159,788	104,675	1,153,056	-382	969	Dominant	108,051	671
H1-MH2-LM3-L3‡	1,128,890	-141,426	18,362	1,153,239	-199	182	Dominant	100,639	931
H1-MH2-LM2-L3	1,148,251	-122,064	19,362	1,153,275	-163	36	Dominant	535,500	825
H1-MH1-LM1-L3	1,207,717	-62,599	78,827	1,153,299	-138	61	Dominant	1,297,747	290
H1-MH1-LM3-L3	1,156,443	-113,872	27,553	1,153,340	-98	101	Dominant	271,870	901
H1-MH1-LM2-L3	1,175,805	-94,511	46,915	1,153,376	-62	138	Dominant	341,191	795
H1-MH2-LM2-L2‡	1,172,487	-97,828	43,598	1,153,418	-20	180	Dominant	242,849	931
H1-MH1-LM1-L1 (status quo strategy)	1,270,316	NA	97,828	1,153,438	NA	20	NA	4,999,857	NA
H1-MH1-LM1-L2	1,231,953	-38,363	59,465	1,153,443	5	25	Cost-saving	2,418,750	396
H1-MH1-LM2-L2‡	1,200,041	-70,275	27,553	1,153,520	82	101	Cost-saving	271,870	901

‡ These strategies comprised the cost-effectiveness efficiency frontier. QALY, quality-adjusted life year; ICER, the incremental cost-effectiveness ratio; iNMB, incremental net monetary benefit

Table S9 Cost-effectiveness estimates for lung cancer screening scenarios ordered by QALYs (overdiagnosis rate when screening: 0.0465)

Strategy	Costs per 100,000 people (CNY, thousand)	Incremental cost per 100,000 people (CNY, thousand)		QALYs per 100,000 people	Incremental QALYs per 100,000 people		ICER (CNY/QALY)		iNMB
		Vs the status quo strategy	Vs the strategy preceding it on the efficiency frontier		Vs the status quo strategy	Vs the strategy preceding it on the efficiency frontier	Vs the status quo strategy	Vs the strategy preceding it on the efficiency frontier	
No screening	843,572	-444,627	NA	1,150,630	-3,133	NA	Dominated	NA	-3,165
H1-MHnone-LMnone-Lnone	924,982	-363,217	81,410	1,151,391	-2,373	761	Dominated	107,047	-2,132
H1-MHone-off-LMone-off-Lnone	948,521	-339,678	104,949	1,151,440	-2,324	809	Dominated	129,648	-2,248
H1-MHone-off-LMone-off-Lone-off	959,835	-328,364	116,262	1,151,441	-2,323	810	Dominated	143,492	-2,359
H1-MHone-off-LMnone-Lnone	938,820	-349,380	95,247	1,151,487	-2,276	857	Dominated	111,173	-2,036
H1-MH3-LMone-off-Lnone	1,001,135	-287,065	157,562	1,152,302	-1,461	1,672	Dominated	94,243	-679
H1-MH3-LMone-off-Lone-off	1,012,448	-275,751	168,876	1,152,303	-1,461	1,673	Dominated	100,964	-791
H1-MH3-LMnone-Lnone‡	991,433	-296,767	147,860	1,152,350	-1,414	1,719	Dominated	86,008	-467
H1-MH2-LM3-Lnone	1,045,303	-242,896	53,870	1,152,441	-1,322	92	Dominated	587,549	-783
H1-MH2-LMone-off-Lnone	1,020,765	-267,435	29,332	1,152,475	-1,289	125	Dominated	234,243	-457
H1-MH2-LMone-off-Lone-off	1,032,078	-256,121	40,645	1,152,475	-1,288	126	Dominated	322,669	-568
H1-MH1-LM1-Lnone	1,129,190	-159,009	137,757	1,152,478	-1,286	128	Dominated	1,072,754	-1,533
H1-MH2-LMnone-Lnone‡	1,011,063	-277,137	19,630	1,152,522	-1,242	172	Dominated	113,811	-245
H1-MH1-LM2-Lnone	1,095,772	-192,427	84,710	1,152,562	-1,202	40	Dominated	2,138,427	-996
H1-MH1-LMone-off-Lnone	1,051,124	-237,075	40,061	1,152,565	-1,198	43	Dominated	926,076	-540
H1-MH1-LMone-off-Lone-off	1,062,437	-225,762	51,375	1,152,566	-1,198	44	Dominated	1,167,469	-652

H1-MH1-LMnone-Lnone	1,041,422	-246,777	30,360	1,152,613	-1,151	91	Dominated	335,394	-328
H1-MH3-LM3-Lnone	1,041,987	-246,212	30,924	1,152,623	-1,141	101	Dominated	306,866	-309
H1-MH3-LM3-Lone-off	1,053,300	-234,899	42,238	1,152,624	-1,140	102	Dominated	416,050	-420
H1-MH2-LM3-Lone-off	1,072,930	-215,269	61,868	1,152,796	-968	274	Dominated	225,794	-198
H1-MH2-LM2-Lnone	1,081,727	-206,473	70,664	1,152,825	-938	303	Dominated	233,106	-215
H1-MH2-LM2-Lone-off	1,093,040	-195,159	81,977	1,152,826	-938	304	Dominated	269,763	-326
H1-MH1-LM1-Lone-off	1,156,817	-131,382	145,755	1,152,833	-931	311	Dominated	469,075	-947
H1-MH1-LM3-Lnone	1,091,976	-196,223	80,914	1,152,886	-878	364	Dominated	222,430	-170
H1-MH1-LM3-Lone-off	1,103,290	-184,909	92,227	1,152,887	-877	365	Dominated	253,011	-281
H1-MH1-LM2-Lone-off	1,123,400	-164,800	112,337	1,152,916	-847	394	Dominated	284,826	-410
H1-MH3-LM3-L3‡	1,118,056	-170,143	106,994	1,153,438	-325	916	Dominant	116,798	911
H1-MH2-LM3-L3‡	1,137,686	-150,513	19,630	1,153,611	-153	172	Dominant	113,811	1,133
H1-MH2-LM2-L3	1,157,796	-130,403	20,110	1,153,640	-123	30	Dominant	672,866	1,005
H1-MH1-LM1-L3	1,221,573	-66,626	83,887	1,153,647	-116	37	Dominant	2,284,010	384
H1-MH1-LM3-L3	1,168,046	-120,153	30,360	1,153,701	-63	91	Dominant	335,394	1,050
H1-MH1-LM2-L3	1,188,155	-100,044	50,469	1,153,731	-33	120	Dominant	419,160	921
H1-MH1-LM1-L1 (status quo strategy)	1,288,199	NA	150,513	1,153,764	NA	153	NA	983,614	NA
H1-MH2-LM2-L2‡	1,183,210	-104,989	45,524	1,153,773	9	163	Cost-saving	280,130	1,073
H1-MH1-LM1-L2	1,246,988	-41,212	63,777	1,153,780	16	7	Cost-saving	9,322,304	452
H1-MH1-LM2-L2‡	1,213,570	-74,629	30,360	1,153,864	100	91	Cost-saving	335,394	989

‡ These strategies comprised the cost-effectiveness efficiency frontier. QALY, quality-adjusted life year; ICER, the incremental cost-effectiveness ratio; iNMB, incremental net monetary benefit

Table S10 Cost-effectiveness estimates for lung cancer screening scenarios ordered by QALYs (Excess relative risk of LC per screening: 0.0003)

Strategy	Costs per 100,000 people (CNY, thousand)	Incremental cost per 100,000 people (CNY, thousand)		QALYs per 100,000 people	Incremental QALYs per 100,000 people		ICER (CNY/QALY)		iNMB
		Vs the status quo strategy	Vs the strategy preceding it on the efficiency frontier		Vs the status quo strategy	Vs the strategy preceding it on the efficiency frontier	Vs the status quo strategy	Vs the strategy preceding it on the efficiency frontier	
No screening	845,956	-433,488	NA	1,150,235	-3,343	NA	Dominated	NA	-3,785
H1-MHnone-LMnone-Lnone	924,995	-354,449	79,039	1,151,039	-2,539	804	Dominated	98,354	-2,623
H1-MHone-off-LMone-off-Lnone	948,096	-331,348	102,139	1,151,096	-2,482	861	Dominated	118,628	-2,715
H1-MHone-off-LMone-off-Lone-off	959,234	-320,210	113,277	1,151,100	-2,477	865	Dominated	130,916	-2,816
H1-MHone-off-LMnone-Lnone	938,466	-340,978	92,509	1,151,142	-2,436	907	Dominated	102,015	-2,507
H1-MH3-LMone-off-Lnone	999,772	-279,672	153,815	1,151,992	-1,586	1,757	Dominated	87,557	-1,056
H1-MH3-LMone-off-Lone-off	1,010,910	-268,534	164,954	1,151,996	-1,581	1,761	Dominated	93,669	-1,157
H1-MH3-LMnone-Lnone‡	990,142	-289,302	144,186	1,152,038	-1,540	1,803	Dominated	79,989	-848
H1-MH2-LM3-Lnone	1,042,383	-237,061	52,241	1,152,151	-1,427	113	Dominated	461,929	-1,096
H1-MH2-LMone-off-Lnone	1,018,720	-260,724	28,578	1,152,175	-1,403	137	Dominated	207,995	-800
H1-MH2-LMone-off-Lone-off	1,029,858	-249,586	39,716	1,152,179	-1,398	142	Dominated	280,353	-901
H1-MH1-LM1-Lnone	1,123,466	-155,978	133,324	1,152,217	-1,360	180	Dominated	741,118	-1,744
H1-MH2-LMnone-Lnone‡	1,009,090	-270,353	18,948	1,152,221	-1,357	183	Dominated	103,419	-592
H1-MH1-LMone-off-Lnone	1,047,515	-231,929	38,424	1,152,280	-1,297	59	Dominated	646,796	-832
H1-MH1-LMone-off-Lone-off	1,058,653	-220,791	49,563	1,152,284	-1,293	64	Dominated	778,375	-933
H1-MH1-LM2-Lnone	1,090,887	-188,556	81,797	1,152,292	-1,285	72	Dominated	1,141,442	-1,236

H1-MH1-LMnone-Lnone	1,037,885	-241,559	28,795	1,152,326	-1,251	105	Dominated	273,640	-625
H1-MH3-LM3-Lnone	1,040,141	-239,303	31,050	1,152,330	-1,247	109	Dominated	284,274	-637
H1-MH3-LM3-Lone-off	1,051,279	-228,165	42,188	1,152,334	-1,243	113	Dominated	371,725	-738
H1-MH2-LM3-Lone-off	1,070,227	-209,217	61,137	1,152,517	-1,060	297	Dominated	206,047	-483
H1-MH2-LM2-Lnone	1,078,799	-200,645	69,708	1,152,550	-1,028	329	Dominated	211,876	-490
H1-MH2-LM2-Lone-off	1,089,937	-189,507	80,846	1,152,554	-1,023	333	Dominated	242,584	-591
H1-MH1-LM1-Lone-off	1,151,309	-128,134	142,219	1,152,584	-993	364	Dominated	391,234	-1,131
H1-MH1-LM3-Lnone	1,087,884	-191,560	78,793	1,152,618	-959	398	Dominated	198,136	-414
H1-MH1-LM3-Lone-off	1,099,022	-180,422	89,932	1,152,623	-955	402	Dominated	223,743	-515
H1-MH1-LM2-Lone-off	1,118,731	-160,713	109,641	1,152,659	-918	438	Dominated	250,037	-623
H1-MH3-LM3-L3‡	1,115,010	-164,433	105,920	1,153,185	-393	964	Dominant	109,880	690
H1-MH2-LM3-L3‡	1,133,959	-145,485	18,948	1,153,368	-210	183	Dominant	103,419	946
H1-MH2-LM2-L3	1,153,668	-125,776	19,709	1,153,404	-173	37	Dominant	539,123	838
H1-MH1-LM1-L3	1,215,041	-64,403	81,082	1,153,435	-143	67	Dominant	1,213,782	297
H1-MH1-LM3-L3	1,162,753	-116,690	28,795	1,153,473	-104	105	Dominant	273,640	913
H1-MH1-LM2-L3	1,182,463	-96,981	48,504	1,153,510	-68	142	Dominant	342,093	805
H1-MH2-LM2-L2‡	1,178,448	-100,995	44,490	1,153,548	-29	180	Dominant	246,775	939
H1-MH1-LM1-L1 (status quo strategy)	1,279,444	NA	100,995	1,153,577	NA	29	NA	3,451,772	NA
H1-MH1-LM1-L2	1,239,821	-39,623	61,373	1,153,578	1	30	Cost-saving	2,029,325	399
H1-MH1-LM2-L2‡	1,207,243	-72,201	28,795	1,153,653	76	105	Cost-saving	273,640	907

‡ These strategies comprised the cost-effectiveness efficiency frontier. QALY, quality-adjusted life year; ICER, the incremental cost-effectiveness ratio; iNMB, incremental net monetary benefit

Table S11 Cost-effectiveness estimates for lung cancer screening scenarios ordered by QALYs (Excess relative risk of LC per screening: 0.0019)

Strategy	Costs per 100,000 people (CNY, thousand)	Incremental cost per 100,000 people (CNY, thousand)		QALYs per 100,000 people	Incremental QALYs per 100,000 people		ICER (CNY/QALY)		iNMB
		Vs the status quo strategy	Vs the strategy preceding it on the efficiency frontier		Vs the status quo strategy	Vs the strategy preceding it on the efficiency frontier	Vs the status quo strategy	Vs the strategy preceding it on the efficiency frontier	
No screening	844,310	-434,745	NA	1,150,508	-3,118	NA	Dominated	NA	-3,227
H1-MHnone-LMnone-Lnone	923,617	-355,438	79,307	1,151,265	-2,360	757	Dominated	104,718	-2,180
H1-MHone-off-LMone-off-Lnone	946,762	-332,293	102,452	1,151,313	-2,312	805	Dominated	127,203	-2,295
H1-MHone-off-LMone-off-Lone-off	957,915	-321,140	113,605	1,151,314	-2,312	806	Dominated	140,999	-2,405
H1-MHone-off-LMnone-Lnone	937,125	-341,930	92,815	1,151,361	-2,265	853	Dominated	108,831	-2,083
H1-MH3-LMone-off-Lnone	998,360	-280,695	154,050	1,152,177	-1,449	1,669	Dominated	92,286	-712
H1-MH3-LMone-off-Lone-off	1,009,514	-269,541	165,203	1,152,178	-1,448	1,670	Dominated	98,950	-823
H1-MH3-LMnone-Lnone‡	988,723	-290,332	144,413	1,152,225	-1,401	1,717	Dominated	84,123	-500
H1-MH2-LM3-Lnone	1,041,306	-237,750	52,582	1,152,313	-1,313	89	Dominated	593,739	-811
H1-MH1-LM1-Lnone	1,122,962	-156,093	134,238	1,152,342	-1,283	118	Dominated	1,140,701	-1,557
H1-MH2-LMone-off-Lnone	1,017,407	-261,648	28,684	1,152,348	-1,277	124	Dominated	231,694	-487
H1-MH2-LMone-off-Lone-off	1,028,561	-250,494	39,838	1,152,349	-1,277	124	Dominated	321,009	-597
H1-MH2-LMnone-Lnone‡	1,007,771	-271,284	19,047	1,152,396	-1,230	171	Dominated	111,245	-275
H1-MH1-LM2-Lnone	1,090,203	-188,853	82,432	1,152,428	-1,198	32	Dominated	2,546,825	-1,021
H1-MH1-LMone-off-Lnone	1,046,540	-232,515	38,769	1,152,434	-1,191	38	Dominated	1,008,922	-569
H1-MH1-LMone-off-Lone-off	1,057,694	-221,361	49,923	1,152,435	-1,191	39	Dominated	1,289,102	-680

H1-MH1-LMnone-Lnone	1,036,903	-242,152	29,133	1,152,482	-1,144	86	Dominated	339,365	-358
H1-MH3-LM3-Lnone	1,038,693	-240,362	30,923	1,152,499	-1,127	103	Dominated	300,785	-334
H1-MH3-LM3-Lone-off	1,049,847	-229,208	42,076	1,152,499	-1,127	103	Dominated	408,084	-445
H1-MH2-LM3-Lone-off	1,068,894	-210,161	61,124	1,152,670	-956	274	Dominated	222,814	-220
H1-MH2-LM2-Lnone	1,077,505	-201,550	69,734	1,152,699	-927	303	Dominated	229,989	-236
H1-MH1-LM1-Lone-off	1,150,550	-128,505	142,780	1,152,699	-926	303	Dominated	470,528	-966
H1-MH2-LM2-Lone-off	1,088,659	-190,397	80,888	1,152,699	-926	304	Dominated	266,511	-347
H1-MH1-LM3-Lnone	1,086,873	-192,182	79,103	1,152,756	-870	360	Dominated	219,809	-192
H1-MH1-LM3-Lone-off	1,098,027	-181,028	90,257	1,152,756	-870	360	Dominated	250,593	-303
H1-MH1-LM2-Lone-off	1,117,791	-161,264	110,021	1,152,785	-841	389	Dominated	282,574	-429
H1-MH3-LM3-L3‡	1,113,511	-165,544	105,740	1,153,315	-311	919	Dominant	115,060	900
H1-MH2-LM3-L3‡	1,132,558	-146,497	19,047	1,153,486	-140	171	Dominant	111,245	1,126
H1-MH1-LM1-L3	1,214,214	-64,841	81,656	1,153,515	-111	29	Dominant	2,804,187	380
H1-MH2-LM2-L3	1,152,322	-126,733	19,764	1,153,515	-111	29	Dominant	677,321	999
H1-MH1-LM3-L3	1,161,691	-117,364	29,133	1,153,572	-54	86	Dominant	339,365	1,043
H1-MH1-LM2-L3	1,181,455	-97,600	48,897	1,153,601	-25	115	Dominant	425,099	916
H1-MH1-LM1-L1 (status quo strategy)	1,279,055	NA	146,497	1,153,626	NA	140	NA	1,048,698	NA
H1-MH1-LM1-L2	1,239,088	-39,967	106,530	1,153,647	21	161	Cost-saving	662,114	451
H1-MH2-LM2-L2‡	1,177,197	-101,859	44,638	1,153,647	21	161	Cost-saving	277,335	1,070
H1-MH1-LM2-L2‡	1,206,329	-72,726	29,133	1,153,733	107	86	Cost-saving	339,365	987

‡ These strategies comprised the cost-effectiveness efficiency frontier. QALY, quality-adjusted life year; ICER, the incremental cost-effectiveness ratio; iNMB, incremental net monetary benefit

Table S12 Cost-effectiveness estimates for lung cancer screening scenarios ordered by QALYs (Biopsy diagnosis cost: decreased by 50%)

Strategy	Costs per 100,000 people (CNY, thousand)	Incremental cost per 100,000 people (CNY, thousand)		QALYs per 100,000 people	Incremental QALYs per 100,000 people		ICER (CNY/QALY)		iNMB
		Vs the status quo strategy	Vs the strategy preceding it on the efficiency frontier		Vs the status quo strategy	Vs the strategy preceding it on the efficiency frontier	Vs the status quo strategy	Vs the strategy preceding it on the efficiency frontier	
No screening	844,685	-329,468	NA	1,150,366	-3,235	NA	Dominated	NA	-4,564
H1-MHnone-LMnone-Lnone	906,508	-267,644	61,824	1,151,147	-2,453	781	Dominated	79,115	-3,284
H1-MHone-off-LMone-off-Lnone	923,115	-251,037	78,431	1,151,200	-2,400	834	Dominated	93,999	-3,321
H1-MHone-off-LMone-off-Lone-off	930,915	-243,237	86,231	1,151,202	-2,398	837	Dominated	103,056	-3,393
H1-MHone-off-LMnone-Lnone	916,676	-257,477	71,991	1,151,247	-2,354	881	Dominated	81,719	-3,144
H1-MH3-LMone-off-Lnone	966,834	-207,319	122,149	1,152,081	-1,520	1,715	Dominated	71,230	-1,619
H1-MH3-LMone-off-Lone-off	974,634	-199,519	129,949	1,152,083	-1,518	1,717	Dominated	75,675	-1,692
H1-MH3-LMnone-Lnone‡	960,394	-213,758	115,709	1,152,127	-1,473	1,761	Dominated	65,691	-1,442
H1-MH2-LM3-Lnone	996,093	-178,060	35,699	1,152,229	-1,372	101	Dominated	352,264	-1,553
H1-MH2-LMone-off-Lnone	981,425	-192,727	21,031	1,152,258	-1,343	131	Dominated	160,687	-1,334
H1-MH2-LMone-off-Lone-off	989,226	-184,927	28,832	1,152,260	-1,340	133	Dominated	216,371	-1,406
H1-MH1-LM1-Lnone	1,052,764	-121,389	92,370	1,152,277	-1,323	150	Dominated	615,434	-2,001
H1-MH2-LMnone-Lnone‡	974,986	-199,167	14,592	1,152,305	-1,296	177	Dominated	82,222	-1,157
H1-MH1-LMone-off-Lnone	1,001,876	-172,276	26,890	1,152,354	-1,247	49	Dominated	544,828	-1,306
H1-MH1-LMone-off-Lone-off	1,009,677	-164,476	34,691	1,152,356	-1,244	52	Dominated	670,709	-1,378
H1-MH1-LM2-Lnone	1,030,707	-143,445	55,721	1,152,357	-1,243	53	Dominated	1,054,618	-1,585
H1-MH1-LMnone-Lnone	995,437	-178,716	20,451	1,152,401	-1,200	96	Dominated	213,159	-1,128

H1-MH3-LM3-Lnone	997,956	-176,196	22,970	1,152,411	-1,190	106	Dominated	216,395	-1,128
H1-MH3-LM3-Lone-off	1,005,757	-168,396	30,771	1,152,413	-1,187	109	Dominated	283,556	-1,201
H1-MH2-LM3-Lone-off	1,020,349	-153,804	45,363	1,152,591	-1,010	286	Dominated	158,617	-915
H1-MH2-LM2-Lnone	1,026,712	-147,441	51,726	1,152,621	-979	317	Dominated	163,356	-905
H1-MH2-LM2-Lone-off	1,034,512	-139,640	59,526	1,152,624	-977	319	Dominated	186,596	-977
H1-MH1-LM1-Lone-off	1,077,020	-97,133	102,034	1,152,639	-961	335	Dominated	304,819	-1,364
H1-MH1-LM3-Lnone	1,032,999	-141,153	58,013	1,152,684	-916	380	Dominated	152,842	-815
H1-MH1-LM3-Lone-off	1,040,800	-133,353	65,814	1,152,687	-914	382	Dominated	172,318	-887
H1-MH1-LM2-Lone-off	1,054,963	-119,189	79,977	1,152,720	-881	415	Dominated	192,738	-948
H1-MH3-LM3-L3‡	1,057,498	-116,654	82,512	1,153,247	-354	942	Dominant	87,553	308
H1-MH2-LM3-L3‡	1,072,090	-102,063	14,592	1,153,425	-176	177	Dominant	82,222	593
H1-MH2-LM2-L3	1,086,254	-87,899	14,164	1,153,458	-143	33	Dominant	428,896	531
H1-MH1-LM1-L3	1,128,761	-45,391	56,671	1,153,473	-127	49	Dominant	1,162,532	145
H1-MH1-LM3-L3	1,092,541	-81,612	20,451	1,153,520	-80	96	Dominant	213,159	621
H1-MH1-LM2-L3	1,106,704	-67,448	34,615	1,153,554	-47	129	Dominant	268,402	560
H1-MH2-LM2-L2‡	1,104,407	-69,745	32,317	1,153,596	-5	171	Dominant	188,965	685
H1-MH1-LM1-L1 (status quo strategy)	1,174,153	NA	69,745	1,153,601	NA	5	NA	13,798,067	NA
H1-MH1-LM1-L2	1,146,915	-27,238	42,508	1,153,611	11	5	Cost-saving	8,409,517	298
H1-MH1-LM2-L2‡	1,124,858	-49,294	20,451	1,153,692	91	5	Cost-saving	4,045,924	714

‡ These strategies comprised the cost-effectiveness efficiency frontier. QALY, quality-adjusted life year; ICER, the incremental cost-effectiveness ratio; iNMB, incremental net monetary benefit

Table S13 Cost-effectiveness estimates for lung cancer screening scenarios ordered by QALYs (Biopsy diagnosis cost: increased by 50%)

Strategy	Costs per 100,000 people (CNY, thousand)	Incremental cost per 100,000 people (CNY, thousand)		QALYs per 100,000 people	Incremental QALYs per 100,000 people		ICER (CNY/QALY)		iNMB
		Vs the status quo strategy	Vs the strategy preceding it on the efficiency frontier		Vs the status quo strategy	Vs the strategy preceding it on the efficiency frontier	Vs the status quo strategy	Vs the strategy preceding it on the efficiency frontier	
No screening	845,651	-538,712	NA	1,150,366	-3,235	NA	Dominated	NA	-2,471
H1-MHnone-LMnone-Lnone	942,161	-442,201	96,511	1,151,147	-2,453	781	Dominated	123,503	-1,538
H1-MHone-off-LMone-off-Lnone	971,798	-412,565	126,147	1,151,200	-2,400	834	Dominated	151,188	-1,706
H1-MHone-off-LMone-off-Lone-off	986,289	-398,074	140,638	1,151,202	-2,398	837	Dominated	168,079	-1,845
H1-MHone-off-LMnone-Lnone	958,971	-425,391	113,320	1,151,247	-2,354	881	Dominated	128,633	-1,464
H1-MH3-LMone-off-Lnone‡	1,031,357	-353,005	185,706	1,152,081	-1,520	1,715	Dominated	108,293	-163
H1-MH3-LMone-off-Lone-off	1,045,848	-338,515	200,197	1,152,083	-1,518	1,717	Dominated	116,583	-302
H1-MH3-LMnone-Lnone	1,018,531	-365,832	172,880	1,152,127	-1,473	1,761	Dominated	98,147	79
H1-MH2-LM3-Lnone	1,087,641	-296,721	69,111	1,152,229	-1,372	101	Dominated	681,961	-366
H1-MH2-LMone-off-Lnone	1,054,757	-329,606	36,226	1,152,258	-1,343	131	Dominated	276,781	35
H1-MH2-LMone-off-Lone-off	1,069,248	-315,115	50,717	1,152,260	-1,340	133	Dominated	380,614	-105
H1-MH1-LM1-Lnone	1,193,684	-190,678	175,154	1,152,277	-1,323	150	Dominated	1,166,999	-1,308
H1-MH2-LMnone-Lnone‡	1,041,930	-342,432	23,400	1,152,305	-1,296	177	Dominated	131,851	276
H1-MH1-LMone-off-Lnone	1,092,219	-292,143	50,289	1,152,354	-1,247	49	Dominated	1,018,909	-107
H1-MH1-LMone-off-Lone-off	1,106,710	-277,653	64,780	1,152,356	-1,244	52	Dominated	1,252,452	-246
H1-MH1-LM2-Lnone	1,150,411	-233,951	108,481	1,152,357	-1,243	53	Dominated	2,053,182	-680

H1-MH1-LMnone-Lnone	1,079,393	-304,970	37,462	1,152,401	-1,200	96	Dominated	390,469	135
H1-MH3-LM3-Lnone	1,080,938	-303,425	39,008	1,152,411	-1,190	106	Dominated	367,477	144
H1-MH3-LM3-Lone-off	1,095,429	-288,934	53,499	1,152,413	-1,187	109	Dominated	492,998	5
H1-MH2-LM3-Lone-off	1,118,829	-265,534	76,898	1,152,591	-1,010	286	Dominated	268,887	202
H1-MH2-LM2-Lnone	1,129,646	-254,717	87,715	1,152,621	-979	317	Dominated	277,015	168
H1-MH2-LM2-Lone-off	1,144,136	-240,226	102,206	1,152,624	-977	319	Dominated	320,384	29
H1-MH1-LM1-Lone-off	1,224,872	-159,491	182,942	1,152,639	-961	335	Dominated	546,525	-740
H1-MH1-LM3-Lnone	1,141,800	-242,562	99,870	1,152,684	-916	380	Dominated	263,118	199
H1-MH1-LM3-Lone-off	1,156,291	-228,072	114,361	1,152,687	-914	382	Dominated	299,429	60
H1-MH1-LM2-Lone-off	1,181,599	-202,764	139,669	1,152,720	-881	415	Dominated	336,589	-113
H1-MH3-LM3-L3‡	1,171,086	-213,277	129,156	1,153,247	-354	942	Dominant	137,047	1,274
H1-MH2-LM3-L3‡	1,194,486	-189,877	23,400	1,153,425	-176	177	Dominant	131,851	1,471
H1-MH2-LM2-L3	1,219,793	-164,569	25,308	1,153,458	-143	33	Dominant	766,359	1,298
H1-MH1-LM1-L3	1,300,529	-83,834	106,043	1,153,473	-127	49	Dominant	2,175,331	529
H1-MH1-LM3-L3	1,231,948	-152,415	37,462	1,153,520	-80	96	Dominant	390,469	1,329
H1-MH1-LM2-L3	1,257,256	-127,107	62,770	1,153,554	-47	129	Dominant	486,721	1,157
H1-MH2-LM2-L2‡	1,251,290	-133,073	56,804	1,153,596	-5	171	Dominant	332,142	1,318
H1-MH1-LM1-L1 (status quo strategy)	1,384,363	NA	133,073	1,153,601	NA	5	NA	26,326,551	NA
H1-MH1-LM1-L2	1,332,025	-52,337	80,735	1,153,611	11	5	Cost-saving	15,972,344	549
H1-MH1-LM2-L2‡	1,288,752	-95,610	37,462	1,153,692	91	5	Cost-saving	7,411,394	1,177

‡ These strategies comprised the cost-effectiveness efficiency frontier. QALY, quality-adjusted life year; ICER, the incremental cost-effectiveness ratio; iNMB, incremental net monetary benefit

Table S14 Cost-effectiveness estimates for lung cancer screening scenarios ordered by QALYs (LDCT test cost: decreased by 50%)

Strategy	Costs per 100,000 people (CNY, thousand)	Incremental cost per 100,000 people (CNY, thousand)		QALYs per 100,000 people	Incremental QALYs per 100,000 people		ICER (CNY/QALY)		iNMB
		Vs the status quo strategy	Vs the strategy preceding it on the efficiency frontier		Vs the status quo strategy	Vs the strategy preceding it on the efficiency frontier	Vs the status quo strategy	Vs the strategy preceding it on the efficiency frontier	
No screening	845,065	-341,431	NA	1,150,366	-3,235	NA	Dominated	NA	-4,444
H1-MHnone-LMnone-Lnone	908,908	-277,588	63,843	1,151,147	-2,453	781	Dominated	81,699	-3,184
H1-MHone-off-LMone-off-Lnone	926,241	-260,254	81,177	1,151,200	-2,400	834	Dominated	97,290	-3,229
H1-MHone-off-LMone-off-Lone-off	934,412	-252,084	89,347	1,151,202	-2,398	837	Dominated	106,780	-3,305
H1-MHone-off-LMnone-Lnone	919,452	-267,044	74,387	1,151,247	-2,354	881	Dominated	84,438	-3,048
H1-MH3-LMone-off-Lnone‡	970,952	-215,543	125,888	1,152,081	-1,520	1,715	Dominated	73,411	-1,537
H1-MH3-LMone-off-Lone-off	979,123	-207,373	134,058	1,152,083	-1,518	1,717	Dominated	78,067	-1,613
H1-MH3-LMnone-Lnone	964,163	-222,333	119,098	1,152,127	-1,473	1,761	Dominated	67,614	-1,356
H1-MH2-LM3-Lnone	1,001,686	-184,810	37,523	1,152,229	-1,372	101	Dominated	370,267	-1,485
H1-MH2-LMone-off-Lnone	986,047	-200,449	21,884	1,152,258	-1,343	131	Dominated	167,200	-1,257
H1-MH2-LMone-off-Lone-off	994,217	-192,279	30,054	1,152,260	-1,340	133	Dominated	225,545	-1,333
H1-MH1-LM1-Lnone	1,061,089	-125,407	96,926	1,152,277	-1,323	150	Dominated	645,791	-1,961
H1-MH2-LMnone-Lnone‡	979,257	-207,239	15,094	1,152,305	-1,296	177	Dominated	85,051	-1,076
H1-MH1-LMone-off-Lnone	1,007,440	-179,056	28,183	1,152,354	-1,247	49	Dominated	571,027	-1,238
H1-MH1-LMone-off-Lone-off	1,015,610	-170,885	36,354	1,152,356	-1,244	52	Dominated	702,861	-1,314
H1-MH1-LM2-Lnone	1,037,865	-148,631	58,609	1,152,357	-1,243	53	Dominated	1,109,262	-1,534

H1-MH1-LMnone-Lnone	1,000,650	-185,846	21,394	1,152,401	-1,200	96	Dominated	222,985	-1,057
H1-MH3-LM3-Lnone	1,003,142	-183,354	23,886	1,152,411	-1,190	106	Dominated	225,015	-1,057
H1-MH3-LM3-Lone-off	1,011,312	-175,183	32,056	1,152,413	-1,187	109	Dominated	295,398	-1,133
H1-MH2-LM3-Lone-off	1,026,406	-160,089	47,150	1,152,591	-1,010	286	Dominated	164,866	-853
H1-MH2-LM2-Lnone	1,033,022	-153,474	53,765	1,152,621	-979	317	Dominated	169,797	-844
H1-MH2-LM2-Lone-off	1,041,192	-145,304	61,936	1,152,624	-977	319	Dominated	194,148	-920
H1-MH1-LM1-Lone-off	1,085,809	-100,687	106,553	1,152,639	-961	335	Dominated	318,318	-1,328
H1-MH1-LM3-Lnone	1,039,630	-146,866	60,373	1,152,684	-916	380	Dominated	159,060	-758
H1-MH1-LM3-Lone-off	1,047,800	-138,696	68,543	1,152,687	-914	382	Dominated	179,466	-833
H1-MH1-LM2-Lone-off	1,062,586	-123,910	83,329	1,152,720	-881	415	Dominated	200,816	-901
H1-MH3-LM3-L3‡	1,064,480	-122,016	85,223	1,153,247	-354	942	Dominant	90,430	361
H1-MH2-LM3-L3‡	1,079,574	-106,922	15,094	1,153,425	-176	177	Dominant	85,051	641
H1-MH2-LM2-L3	1,094,360	-92,136	14,786	1,153,458	-143	33	Dominant	447,736	574
H1-MH1-LM1-L3	1,138,977	-47,519	59,403	1,153,473	-127	49	Dominant	1,218,570	166
H1-MH1-LM3-L3	1,100,968	-85,528	21,394	1,153,520	-80	96	Dominant	222,985	661
H1-MH1-LM2-L3	1,115,753	-70,742	36,179	1,153,554	-47	129	Dominant	280,536	593
H1-MH2-LM2-L2‡	1,113,261	-73,235	33,687	1,153,596	-5	171	Dominant	196,971	720
H1-MH1-LM1-L1 (status quo strategy)	1,186,496	NA	73,235	1,153,601	NA	5	NA	14,488,529	NA
H1-MH1-LM1-L2	1,157,878	-28,618	44,617	1,153,611	11	5	Cost-saving	8,826,862	312
H1-MH1-LM2-L2‡	1,134,654	-51,842	21,394	1,153,692	91	5	Cost-saving	4,232,425	739

‡ These strategies comprised the cost-effectiveness efficiency frontier. QALY, quality-adjusted life year; ICER, the incremental cost-effectiveness ratio; iNMB, incremental net monetary benefit

Table S15 Cost-effectiveness estimates for lung cancer screening scenarios ordered by QALYs (LDCT test cost: increased by 50%)

Strategy	Costs per 100,000 people (CNY, thousand)	Incremental cost per 100,000 people (CNY, thousand)		QALYs per 100,000 people	Incremental QALYs per 100,000 people		ICER (CNY/QALY)		iNMB
		Vs the status quo strategy	Vs the strategy preceding it on the efficiency frontier		Vs the status quo strategy	Vs the strategy preceding it on the efficiency frontier	Vs the status quo strategy	Vs the strategy preceding it on the efficiency frontier	
No screening	845,271	-526,749	NA	1,150,366	-3,235	NA	Dominated	NA	-2,591
H1-MHnone-LMnone-Lnone	939,762	-432,257	94,491	1,151,147	-2,453	781	Dominated	120,919	-1,637
H1-MHone-off-LMone-off-Lnone	968,672	-403,348	123,401	1,151,200	-2,400	834	Dominated	147,897	-1,798
H1-MHone-off-LMone-off-Lone-off	982,793	-389,227	137,522	1,151,202	-2,398	837	Dominated	164,355	-1,933
H1-MHone-off-LMnone-Lnone	956,195	-415,824	110,925	1,151,247	-2,354	881	Dominated	125,914	-1,560
H1-MH3-LMone-off-Lnone‡	1,027,238	-344,781	181,968	1,152,081	-1,520	1,715	Dominated	106,113	-245
H1-MH3-LMone-off-Lone-off	1,041,359	-330,660	196,089	1,152,083	-1,518	1,717	Dominated	114,190	-380
H1-MH3-LMnone-Lnone	1,014,762	-357,257	169,491	1,152,127	-1,473	1,761	Dominated	96,224	-7
H1-MH2-LM3-Lnone	1,082,048	-289,971	67,286	1,152,229	-1,372	101	Dominated	663,959	-433
H1-MH2-LMone-off-Lnone	1,050,136	-321,883	35,374	1,152,258	-1,343	131	Dominated	270,268	-43
H1-MH2-LMone-off-Lone-off	1,064,257	-307,763	49,495	1,152,260	-1,340	133	Dominated	371,440	-178
H1-MH1-LM1-Lnone	1,185,360	-186,660	170,598	1,152,277	-1,323	150	Dominated	1,136,643	-1,348
H1-MH2-LMnone-Lnone‡	1,037,660	-334,360	22,898	1,152,305	-1,296	177	Dominated	129,022	195
H1-MH1-LMone-off-Lnone	1,086,656	-285,364	48,996	1,152,354	-1,247	49	Dominated	992,710	-175
H1-MH1-LMone-off-Lone-off	1,100,776	-271,243	63,117	1,152,356	-1,244	52	Dominated	1,220,300	-310
H1-MH1-LM2-Lnone	1,143,253	-228,766	105,594	1,152,357	-1,243	53	Dominated	1,998,537	-732
H1-MH1-LMnone-Lnone	1,074,179	-297,840	36,520	1,152,401	-1,200	96	Dominated	380,643	63

H1-MH3-LM3-Lnone	1,075,752	-296,267	38,093	1,152,411	-1,190	106	Dominated	358,856	72
H1-MH3-LM3-Lone-off	1,089,873	-282,146	52,214	1,152,413	-1,187	109	Dominated	481,156	-63
H1-MH2-LM3-Lone-off	1,112,771	-259,248	75,111	1,152,591	-1,010	286	Dominated	262,638	139
H1-MH2-LM2-Lnone	1,123,335	-248,684	85,676	1,152,621	-979	317	Dominated	270,574	108
H1-MH2-LM2-Lone-off	1,137,456	-234,563	99,797	1,152,624	-977	319	Dominated	312,832	-28
H1-MH1-LM1-Lone-off	1,216,082	-155,937	178,423	1,152,639	-961	335	Dominated	533,025	-776
H1-MH1-LM3-Lnone	1,135,170	-236,850	97,510	1,152,684	-916	380	Dominated	256,901	142
H1-MH1-LM3-Lone-off	1,149,291	-222,729	111,631	1,152,687	-914	382	Dominated	292,281	7
H1-MH1-LM2-Lone-off	1,173,976	-198,043	136,317	1,152,720	-881	415	Dominated	328,511	-160
H1-MH3-LM3-L3‡	1,164,104	-207,915	126,444	1,153,247	-354	942	Dominant	134,170	1,220
H1-MH2-LM3-L3‡	1,187,002	-185,018	22,898	1,153,425	-176	177	Dominant	129,022	1,422
H1-MH2-LM2-L3	1,211,687	-160,332	24,686	1,153,458	-143	33	Dominant	747,519	1,256
H1-MH1-LM1-L3	1,290,313	-81,706	103,311	1,153,473	-127	49	Dominant	2,119,294	508
H1-MH1-LM3-L3	1,223,521	-148,498	36,520	1,153,520	-80	96	Dominant	380,643	1,290
H1-MH1-LM2-L3	1,248,207	-123,813	61,205	1,153,554	-47	129	Dominant	474,587	1,124
H1-MH2-LM2-L2‡	1,242,437	-129,583	55,435	1,153,596	-5	171	Dominant	324,137	1,284
H1-MH1-LM1-L1 (status quo strategy)	1,372,019	NA	129,583	1,153,601	NA	5	NA	25,636,089	NA
H1-MH1-LM1-L2	1,321,062	-50,957	78,626	1,153,611	11	5	Cost-saving	15,554,999	535
H1-MH1-LM2-L2‡	1,278,956	-93,063	36,520	1,153,692	91	5	Cost-saving	7,224,893	1,151

‡ These strategies comprised the cost-effectiveness efficiency frontier. QALY, quality-adjusted life year; ICER, the incremental cost-effectiveness ratio; iNMB, incremental net monetary benefit

Table S16 Cost-effectiveness estimates for lung cancer screening scenarios ordered by QALYs (Background medical treatment costs: decreased by 50%)

Strategy	Costs per 100,000 people (CNY, thousand)	Incremental cost per 100,000 people (CNY, thousand)		QALYs per 100,000 people	Incremental QALYs per 100,000 people		ICER (CNY/QALY)		iNMB
		Vs the status quo strategy	Vs the strategy preceding it on the efficiency frontier		Vs the status quo strategy	Vs the strategy preceding it on the efficiency frontier	Vs the status quo strategy	Vs the strategy preceding it on the efficiency frontier	
No screening	824,963	-448,332	NA	1,150,366	-3,235	NA	Dominated	NA	-3,375
H1-MHnone-LMnone-Lnone	907,298	-365,997	82,335	1,151,147	-2,453	781	Dominated	105,362	-2,300
H1-MHone-off-LMone-off-Lnone	930,867	-342,428	105,904	1,151,200	-2,400	834	Dominated	126,926	-2,407
H1-MHone-off-LMone-off-Lone-off	942,164	-331,131	117,201	1,151,202	-2,398	837	Dominated	140,068	-2,514
H1-MHone-off-LMnone-Lnone	921,167	-352,129	96,203	1,151,247	-2,354	881	Dominated	109,203	-2,197
H1-MH3-LMone-off-Lnone‡	985,198	-288,097	160,234	1,152,081	-1,520	1,715	Dominated	93,440	-812
H1-MH3-LMone-off-Lone-off	996,495	-276,800	171,532	1,152,083	-1,518	1,717	Dominated	99,890	-919
H1-MH3-LMnone-Lnone	975,497	-297,798	150,534	1,152,127	-1,473	1,761	Dominated	85,461	-601
H1-MH2-LM3-Lnone	1,028,835	-244,460	53,338	1,152,229	-1,372	101	Dominated	526,318	-889
H1-MH2-LMone-off-Lnone	1,004,867	-268,428	29,370	1,152,258	-1,343	131	Dominated	224,394	-577
H1-MH2-LMone-off-Lone-off	1,016,164	-257,131	40,667	1,152,260	-1,340	133	Dominated	305,188	-684
H1-MH1-LM1-Lnone	1,111,909	-161,386	136,412	1,152,277	-1,323	150	Dominated	908,872	-1,601
H1-MH2-LMnone-Lnone‡	995,166	-278,129	19,669	1,152,305	-1,296	177	Dominated	110,829	-367
H1-MH1-LMone-off-Lnone	1,034,623	-238,672	39,457	1,152,354	-1,247	49	Dominated	799,443	-642
H1-MH1-LMone-off-Lone-off	1,045,920	-227,375	50,754	1,152,356	-1,244	52	Dominated	981,279	-749
H1-MH1-LM2-Lnone	1,078,753	-194,542	83,587	1,152,357	-1,243	53	Dominated	1,582,016	-1,074

H1-MH1-LMnone-Lnone	1,024,923	-248,372	29,756	1,152,401	-1,200	96	Dominated	310,150	-431
H1-MH3-LM3-Lnone	1,026,878	-246,417	31,712	1,152,411	-1,190	106	Dominated	298,746	-426
H1-MH3-LM3-Lone-off	1,038,175	-235,120	43,009	1,152,413	-1,187	109	Dominated	396,334	-533
H1-MH2-LM3-Lone-off	1,057,844	-215,451	62,678	1,152,591	-1,010	286	Dominated	219,163	-299
H1-MH2-LM2-Lnone	1,066,709	-206,586	71,543	1,152,621	-979	317	Dominated	225,940	-313
H1-MH2-LM2-Lone-off	1,078,006	-195,289	82,840	1,152,624	-977	319	Dominated	259,677	-420
H1-MH1-LM1-Lone-off	1,140,918	-132,377	145,752	1,152,639	-961	335	Dominated	435,424	-1,011
H1-MH1-LM3-Lnone	1,076,304	-196,991	81,137	1,152,684	-916	380	Dominated	213,765	-256
H1-MH1-LM3-Lone-off	1,087,601	-185,694	92,435	1,152,687	-914	382	Dominated	242,020	-364
H1-MH1-LM2-Lone-off	1,107,762	-165,533	112,596	1,152,720	-881	415	Dominated	271,347	-485
H1-MH3-LM3-L3‡	1,104,536	-168,759	109,370	1,153,247	-354	942	Dominant	116,052	829
H1-MH2-LM3-L3‡	1,124,205	-149,090	19,669	1,153,425	-176	177	Dominant	110,829	1,063
H1-MH2-LM2-L3	1,144,367	-128,929	20,162	1,153,458	-143	33	Dominant	610,530	942
H1-MH1-LM1-L3	1,207,279	-66,016	83,074	1,153,473	-127	49	Dominant	1,704,153	351
H1-MH1-LM3-L3	1,153,961	-119,334	29,756	1,153,520	-80	96	Dominant	310,150	999
H1-MH1-LM2-L3	1,174,123	-99,172	49,918	1,153,554	-47	129	Dominant	387,066	877
H1-MH2-LM2-L2‡	1,169,846	-103,449	45,642	1,153,596	-5	171	Dominant	266,873	1,022
H1-MH1-LM1-L1 (status quo strategy)	1,273,295	NA	103,449	1,153,601	NA	5	NA	20,465,857	NA
H1-MH1-LM1-L2	1,232,759	-40,536	62,912	1,153,611	11	5	Cost-saving	12,446,320	431
H1-MH1-LM2-L2‡	1,199,603	-73,692	29,756	1,153,692	91	5	Cost-saving	5,886,881	958

‡ These strategies comprised the cost-effectiveness efficiency frontier. QALY, quality-adjusted life year; ICER, the incremental cost-effectiveness ratio; iNMB, incremental net monetary benefit

Table S17 Cost-effectiveness estimates for lung cancer screening scenarios ordered by QALYs (Background medical treatment costs: increased by 50%)

Strategy	Costs per 100,000 people (CNY, thousand)	Incremental cost per 100,000 people (CNY, thousand)		QALYs per 100,000 people	Incremental QALYs per 100,000 people		ICER (CNY/QALY)		iNMB
		Vs the status quo strategy	Vs the strategy preceding it on the efficiency frontier		Vs the status quo strategy	Vs the strategy preceding it on the efficiency frontier	Vs the status quo strategy	Vs the strategy preceding it on the efficiency frontier	
No screening	865,372	-419,848	NA	1,150,366	-3,235	NA	Dominated	NA	-3,660
H1-MHnone-LMnone-Lnone	941,372	-343,848	76,000	1,151,147	-2,453	781	Dominated	97,256	-2,522
H1-MHone-off-LMone-off-Lnone	964,046	-321,174	98,674	1,151,200	-2,400	834	Dominated	118,261	-2,620
H1-MHone-off-LMone-off-Lone-off	975,040	-310,180	109,668	1,151,202	-2,398	837	Dominated	131,066	-2,724
H1-MHone-off-LMnone-Lnone	954,480	-330,740	89,109	1,151,247	-2,354	881	Dominated	101,149	-2,411
H1-MH3-LMone-off-Lnone‡	1,012,993	-272,227	147,621	1,152,081	-1,520	1,715	Dominated	86,084	-970
H1-MH3-LMone-off-Lone-off	1,023,987	-261,233	158,615	1,152,083	-1,518	1,717	Dominated	92,368	-1,074
H1-MH3-LMnone-Lnone	1,003,427	-281,793	138,055	1,152,127	-1,473	1,761	Dominated	78,377	-761
H1-MH2-LM3-Lnone	1,054,899	-230,321	51,472	1,152,229	-1,372	101	Dominated	507,907	-1,030
H1-MH2-LMone-off-Lnone	1,031,315	-253,905	27,888	1,152,258	-1,343	131	Dominated	213,074	-722
H1-MH2-LMone-off-Lone-off	1,042,310	-242,910	38,882	1,152,260	-1,340	133	Dominated	291,797	-827
H1-MH1-LM1-Lnone	1,134,539	-150,681	131,112	1,152,277	-1,323	150	Dominated	873,562	-1,708
H1-MH2-LMnone-Lnone‡	1,021,750	-263,470	18,323	1,152,305	-1,296	177	Dominated	103,243	-514
H1-MH1-LMone-off-Lnone	1,059,472	-225,748	37,722	1,152,354	-1,247	49	Dominated	764,294	-771
H1-MH1-LMone-off-Lone-off	1,070,466	-214,754	48,717	1,152,356	-1,244	52	Dominated	941,883	-875
H1-MH1-LM2-Lnone	1,102,366	-182,854	80,616	1,152,357	-1,243	53	Dominated	1,525,784	-1,191

H1-MH1-LMnone-Lnone	1,049,907	-235,313	28,157	1,152,401	-1,200	96	Dominated	293,478	-562
H1-MH3-LM3-Lnone	1,052,016	-233,204	30,266	1,152,411	-1,190	106	Dominated	285,125	-558
H1-MH3-LM3-Lone-off	1,063,010	-222,210	41,260	1,152,413	-1,187	109	Dominated	380,220	-663
H1-MH2-LM3-Lone-off	1,081,333	-203,887	59,583	1,152,591	-1,010	286	Dominated	208,341	-415
H1-MH2-LM2-Lnone	1,089,648	-195,572	67,898	1,152,621	-979	317	Dominated	214,431	-423
H1-MH2-LM2-Lone-off	1,100,643	-184,577	78,893	1,152,624	-977	319	Dominated	247,303	-528
H1-MH1-LM1-Lone-off	1,160,973	-124,247	139,223	1,152,639	-961	335	Dominated	415,919	-1,093
H1-MH1-LM3-Lnone	1,098,496	-186,724	76,746	1,152,684	-916	380	Dominated	202,195	-359
H1-MH1-LM3-Lone-off	1,109,490	-175,730	87,740	1,152,687	-914	382	Dominated	229,728	-463
H1-MH1-LM2-Lone-off	1,128,799	-156,421	107,050	1,152,720	-881	415	Dominated	257,980	-576
H1-MH3-LM3-L3‡	1,124,048	-161,172	102,298	1,153,247	-354	942	Dominant	108,549	753
H1-MH2-LM3-L3‡	1,142,371	-142,849	18,323	1,153,425	-176	177	Dominant	103,243	1,001
H1-MH2-LM2-L3	1,161,680	-123,540	19,310	1,153,458	-143	33	Dominant	584,725	888
H1-MH1-LM1-L3	1,222,011	-63,209	79,640	1,153,473	-127	49	Dominant	1,633,710	323
H1-MH1-LM3-L3	1,170,528	-114,692	28,157	1,153,520	-80	96	Dominant	293,478	952
H1-MH1-LM2-L3	1,189,837	-95,383	47,466	1,153,554	-47	129	Dominant	368,056	839
H1-MH2-LM2-L2‡	1,185,851	-99,369	43,480	1,153,596	-5	171	Dominant	254,235	981
H1-MH1-LM1-L1 (status quo strategy)	1,285,220	NA	99,369	1,153,601	NA	5	NA	19,658,762	NA
H1-MH1-LM1-L2	1,246,181	-39,039	60,331	1,153,611	11	5	Cost-saving	11,935,541	416
H1-MH1-LM2-L2‡	1,214,008	-71,212	28,157	1,153,692	91	5	Cost-saving	5,570,437	933

‡ These strategies comprised the cost-effectiveness efficiency frontier. QALY, quality-adjusted life year; ICER, the incremental cost-effectiveness ratio; iNMB, incremental net monetary benefit

Table S18 Cost-effectiveness estimates for lung cancer screening scenarios ordered by QALYs (Disutility associated with a false positive screen: decreased by 50%)

Strategy	Costs per 100,000 people (CNY, thousand)	Incremental cost per 100,000 people (CNY, thousand)		QALYs per 100,000 people	Incremental QALYs per 100,000 people		ICER (CNY/QALY)		iNMB
		Vs the status quo strategy	Vs the strategy preceding it on the efficiency frontier		Vs the status quo strategy	Vs the strategy preceding it on the efficiency frontier	Vs the status quo strategy	Vs the strategy preceding it on the efficiency frontier	
No screening	845,168	-434,090	NA	1,150,366	-4,594	NA	Dominated	NA	-6,819
H1-MHnone-LMnone-Lnone	924,335	-354,923	79,167	1,151,372	-3,588	1,006	Dominated	78,694	-5,166
H1-MHone-off-LMone-off-Lnone	947,457	-331,801	102,289	1,151,510	-3,450	1,144	Dominated	89,420	-5,063
H1-MHone-off-LMnone-Lnone	937,823	-341,434	92,656	1,151,514	-3,445	1,149	Dominated	80,659	-4,955
H1-MHone-off-LMone-off-Lone-off	958,602	-320,655	113,434	1,151,556	-3,404	1,190	Dominated	95,325	-5,062
H1-MH3-LMone-off-Lnone	999,095	-280,162	153,928	1,152,491	-2,468	2,126	Dominated	72,417	-3,194
H1-MH3-LMnone-Lnone‡	989,462	-289,795	144,295	1,152,496	-2,463	2,130	Dominated	67,731	-3,086
H1-MH3-LMone-off-Lone-off	1,010,241	-269,017	20,779	1,152,537	-2,422	41	Dominated	503,846	-3,194
H1-MH2-LMone-off-Lnone	1,018,091	-261,166	28,629	1,152,726	-2,233	230	Dominated	124,539	-2,814
H1-MH2-LMnone-Lnone‡	1,008,458	-270,799	18,996	1,152,731	-2,229	235	Dominated	80,937	-2,706
H1-MH2-LMone-off-Lone-off	1,029,237	-250,021	20,779	1,152,772	-2,187	41	Dominated	503,846	-2,814
H1-MH2-LM3-Lnone	1,041,867	-237,391	33,409	1,152,816	-2,143	85	Dominated	391,733	-2,833
H1-MH1-LMone-off-Lnone	1,047,048	-232,210	38,590	1,152,933	-2,026	202	Dominated	190,872	-2,601
H1-MH1-LMnone-Lnone	1,037,415	-241,843	28,957	1,152,938	-2,022	207	Dominated	139,890	-2,493
H1-MH3-LM3-Lnone	1,039,447	-239,810	30,989	1,152,941	-2,018	210	Dominated	147,331	-2,505
H1-MH1-LMone-off-Lone-off	1,058,193	-221,064	49,735	1,152,979	-1,980	248	Dominated	200,355	-2,600

H1-MH3-LM3-Lone-off	1,050,593	-228,665	42,135	1,152,987	-1,972	256	Dominated	164,334	-2,505
H1-MH1-LM2-Lnone	1,090,559	-188,698	82,101	1,153,129	-1,831	398	Dominated	206,302	-2,560
H1-MH1-LM1-Lnone	1,123,224	-156,033	114,766	1,153,187	-1,772	456	Dominated	251,446	-2,745
H1-MH2-LM3-Lone-off	1,069,589	-209,669	61,131	1,153,222	-1,738	491	Dominated	124,478	-2,124
H1-MH2-LM2-Lnone	1,078,179	-201,079	69,721	1,153,282	-1,678	551	Dominated	126,599	-2,065
H1-MH2-LM2-Lone-off	1,089,324	-189,933	80,866	1,153,328	-1,632	597	Dominated	135,504	-2,065
H1-MH1-LM3-Lnone	1,087,400	-191,858	78,942	1,153,383	-1,577	652	Dominated	121,070	-1,911
H1-MH1-LM3-Lone-off	1,098,545	-180,712	90,087	1,153,429	-1,531	698	Dominated	129,048	-1,911
H1-MH1-LM2-Lone-off	1,118,281	-160,977	109,823	1,153,535	-1,425	804	Dominated	136,633	-1,852
H1-MH1-LM1-Lone-off	1,150,946	-128,312	142,488	1,153,593	-1,366	862	Dominated	165,254	-2,036
H1-MH3-LM3-L3‡	1,114,292	-164,966	105,834	1,153,975	-984	1,244	Dominated	85,043	-741
H1-MH2-LM3-L3‡	1,133,288	-145,970	18,996	1,154,210	-749	235	Dominated	80,937	-361
H1-MH2-LM2-L3	1,153,023	-126,234	38,731	1,154,316	-644	340	Dominated	113,787	-302
H1-MH1-LM3-L3	1,162,244	-117,013	47,952	1,154,417	-542	442	Dominated	108,565	-148
H1-MH1-LM2-L3	1,181,980	-97,277	67,688	1,154,523	-437	547	Dominated	123,658	-88
H1-MH2-LM2-L2‡	1,177,849	-101,409	63,557	1,154,541	-419	565	Dominated	112,422	-3
H1-MH1-LM1-L3	1,214,645	-64,613	36,796	1,154,581	-378	40	Dominated	908,598	-273
H1-MH1-LM2-L2‡	1,206,805	-72,452	28,957	1,154,748	-212	207	Dominant	139,890	210
H1-MH1-LM1-L2	1,239,470	-39,787	32,665	1,154,806	-153	58	Dominant	558,769	25
H1-MH1-LM1-L1 (status quo strategy)	1,279,258	NA	72,452	1,154,960	NA	212	NA	342,004	NA

‡ These strategies comprised the cost-effectiveness efficiency frontier. QALY, quality-adjusted life year; ICER, the incremental cost-effectiveness ratio; iNMB, incremental net monetary benefit

Table S19 Cost-effectiveness estimates for lung cancer screening scenarios ordered by QALYs (Disutility associated with a false positive screen: increased by 50%)

Strategy	Costs per 100,000 people (CNY, thousand)	Incremental cost per 100,000 people (CNY, thousand)		QALYs per 100,000 people	Incremental QALYs per 100,000 people		ICER (CNY/QALY)		iNMB
		Vs the status quo strategy	Vs the strategy preceding it on the efficiency frontier		Vs the status quo strategy	Vs the strategy preceding it on the efficiency frontier	Vs the status quo strategy	Vs the strategy preceding it on the efficiency frontier	
No screening	845,168	-434,090	NA	1,150,366	-1,876	NA	Dominated	NA	-216
H1-MHone-off-LMone-off-Lone-off	958,602	-320,655	113,434	1,150,849	-1,392	484	Dominated	234,607	-176
H1-MHone-off-LMone-off-Lnone	947,457	-331,801	102,289	1,150,891	-1,351	525	Dominant	194,897	36
H1-MHnone-LMnone-Lnone	924,335	-354,923	79,167	1,150,923	-1,319	557	Dominant	142,163	345
H1-MHone-off-LMnone-Lnone	937,823	-341,434	92,656	1,150,979	-1,263	613	Dominant	151,105	347
H1-MH1-LM1-Lnone	1,123,224	-156,033	278,056	1,151,367	-874	1,002	Dominated	277,634	-564
H1-MH1-LM2-Lnone	1,090,559	-188,698	245,392	1,151,586	-656	1,220	Dominant	201,072	294
H1-MH3-LMone-off-Lone-off	1,010,241	-269,017	165,073	1,151,629	-613	1,263	Dominant	130,721	1,201
H1-MH2-LM3-Lnone	1,041,867	-237,391	196,699	1,151,641	-601	1,275	Dominant	154,254	914
H1-MH3-LMone-off-Lnone	999,095	-280,162	153,928	1,151,670	-572	1,304	Dominant	118,032	1,412
H1-MH1-LM1-Lone-off	1,150,946	-128,312	305,778	1,151,686	-556	1,320	Dominated	231,660	-68
H1-MH1-LMone-off-Lone-off	1,058,193	-221,064	213,026	1,151,734	-508	1,368	Dominant	155,730	976
H1-MH2-LMone-off-Lone-off	1,029,237	-250,021	184,069	1,151,749	-493	1,383	Dominant	133,091	1,303
H1-MH3-LMnone-Lnone‡	989,462	-289,795	144,295	1,151,758	-484	1,392	Dominant	103,625	1,723
H1-MH1-LMone-off-Lnone	1,047,048	-232,210	57,586	1,151,775	-467	17	Dominant	3,432,088	1,188
H1-MH2-LMone-off-Lnone	1,018,091	-261,166	28,629	1,151,790	-452	32	Dominant	897,729	1,515
H1-MH3-LM3-Lone-off	1,050,593	-228,665	61,131	1,151,839	-403	81	Dominant	755,818	1,309

H1-MH1-LMnone-Lnone	1,037,415	-241,843	47,952	1,151,863	-378	105	Dominant	456,121	1,499
H1-MH2-LMnone-Lnone‡	1,008,458	-270,799	18,996	1,151,878	-363	120	Dominant	157,979	1,825
H1-MH3-LM3-Lnone	1,039,447	-239,810	30,989	1,151,880	-361	2	Dominant	15,779,350	1,520
H1-MH1-LM2-Lone-off	1,118,281	-160,977	109,823	1,151,905	-337	26	Dominant	4,203,035	791
H1-MH2-LM2-Lone-off	1,089,324	-189,933	80,866	1,151,920	-322	41	Dominant	1,960,812	1,117
H1-MH1-LM3-Lone-off	1,098,545	-180,712	90,087	1,151,944	-298	66	Dominant	1,369,768	1,084
H1-MH2-LM3-Lone-off	1,069,589	-209,669	61,131	1,151,959	-282	81	Dominant	755,818	1,411
H1-MH2-LM2-Lnone	1,078,179	-201,079	69,721	1,151,961	-281	83	Dominant	844,404	1,329
H1-MH1-LM3-Lnone	1,087,400	-191,858	78,942	1,151,986	-256	107	Dominant	737,118	1,296
H1-MH1-LM1-L1 (status quo strategy)	1,279,258	NA	270,799	1,152,242	NA	363	NA	745,431	NA
H1-MH1-LM1-L3	1,214,645	-64,613	206,187	1,152,365	124	487	Cost-saving	423,414	947
H1-MH1-LM1-L2	1,239,470	-39,787	231,012	1,152,416	175	538	Cost-saving	429,385	822
H1-MH3-LM3-L3‡	1,114,292	-164,966	105,834	1,152,519	277	640	Cost-saving	165,272	2,323
H1-MH1-LM2-L3	1,181,980	-97,277	67,688	1,152,584	343	65	Cost-saving	1,033,529	1,805
H1-MH2-LM2-L3	1,153,023	-126,234	38,731	1,152,599	358	81	Cost-saving	480,515	2,131
H1-MH1-LM3-L3	1,162,244	-117,013	47,952	1,152,624	382	105	Cost-saving	456,121	2,099
H1-MH1-LM2-L2	1,206,805	-72,452	92,513	1,152,635	394	117	Cost-saving	793,858	1,681
H1-MH2-LM3-L3‡	1,133,288	-145,970	18,996	1,152,639	397	120	Cost-saving	157,979	2,425
H1-MH2-LM2-L2‡	1,177,849	44,561	63,557	1,152,650	409	11	Cost-saving	482,777	2,007

‡ These strategies comprised the cost-effectiveness efficiency frontier. QALY, quality-adjusted life year; ICER, the incremental cost-effectiveness ratio; iNMB, incremental net monetary benefit

Table S20 Cost-effectiveness estimates for lung cancer screening scenarios ordered by QALYs (Adherence: decreased by 50%)

Strategy	Costs per 100,000 people (CNY, thousand)	Incremental cost per 100,000 people (CNY, thousand)		QALYs per 100,000 people	Incremental QALYs per 100,000 people		ICER (CNY/QALY)		iNMB
		Vs the status quo strategy	Vs the strategy preceding it on the efficiency frontier		Vs the status quo strategy	Vs the strategy preceding it on the efficiency frontier	Vs the status quo strategy	Vs the strategy preceding it on the efficiency frontier	
No screening	845,168	-266,274	NA	1,150,366	-2,675	NA	Dominated	NA	-3,835
H1-MHnone-LMnone-Lnone	893,864	-217,578	48,697	1,150,963	-2,077	597	Dominated	81,527	-2,871
H1-MHone-off-LMone-off-Lnone	905,425	-206,017	60,257	1,150,990	-2,051	624	Dominated	96,602	-2,922
H1-MHone-off-LMone-off-Lone-off	910,998	-200,444	65,830	1,150,991	-2,050	625	Dominated	105,336	-2,975
H1-MHone-off-LMnone-Lnone	900,609	-210,833	55,441	1,151,013	-2,028	647	Dominated	85,681	-2,817
H1-MH2-LM3-Lnone	948,200	-163,242	103,032	1,151,446	-1,595	1,080	Dominated	95,415	-2,242
H1-MH3-LMone-off-Lnone	932,268	-179,174	87,100	1,151,462	-1,579	1,096	Dominated	79,479	-2,044
H1-MH3-LMone-off-Lone-off	937,841	-173,601	92,673	1,151,463	-1,578	1,097	Dominated	84,473	-2,097
H1-MH3-LMnone-Lnone‡	927,452	-183,990	82,284	1,151,485	-1,556	1,119	Dominated	73,521	-1,939
H1-MH2-LMone-off-Lnone	944,123	-167,319	16,672	1,151,625	-1,415	140	Dominated	118,927	-1,765
H1-MH2-LMone-off-Lone-off	949,696	-161,746	22,244	1,151,626	-1,414	141	Dominated	157,352	-1,818
H1-MH3-LM3-Lnone	952,921	-158,521	25,469	1,151,642	-1,399	157	Dominated	162,302	-1,812
H1-MH3-LM3-Lone-off	958,494	-152,948	31,042	1,151,643	-1,397	158	Dominated	196,333	-1,865
H1-MH2-LMnone-Lnone‡	939,307	-172,135	11,855	1,151,648	-1,392	163	Dominated	72,518	-1,660
H1-MH1-LM2-Lnone	984,829	-126,613	45,522	1,151,788	-1,253	139	Dominated	326,496	-1,777
H1-MH2-LM3-Lone-off	970,349	-141,093	31,042	1,151,807	-1,234	158	Dominated	196,333	-1,587

H1-MH1-LM1-Lnone	1,009,135	-102,307	69,829	1,151,856	-1,185	207	Dominated	337,148	-1,856
H1-MH2-LM2-Lnone	975,866	-135,576	36,560	1,151,861	-1,179	213	Dominated	171,951	-1,510
H1-MH2-LM2-Lone-off	981,439	-130,003	42,132	1,151,862	-1,178	214	Dominated	197,064	-1,562
H1-MH1-LMone-off-Lnone	969,662	-141,780	30,355	1,151,912	-1,129	263	Dominated	115,302	-1,324
H1-MH1-LMone-off-Lone-off	975,235	-136,207	35,928	1,151,913	-1,128	264	Dominated	135,859	-1,377
H1-MH1-LMnone-Lnone	964,845	-146,597	25,539	1,151,935	-1,106	287	Dominated	89,121	-1,220
H1-MH1-LM3-Lnone	990,315	-121,127	51,008	1,152,092	-949	443	Dominated	115,016	-1,093
H1-MH1-LM3-Lone-off	995,887	-115,555	56,581	1,152,093	-947	445	Dominated	127,242	-1,146
H1-MH3-LM3-L3	991,421	-120,021	52,115	1,152,095	-946	446	Dominated	116,802	-1,098
H1-MH1-LM2-Lone-off	1,006,978	-104,464	67,671	1,152,149	-892	500	Dominated	135,244	-1,122
H1-MH1-LM1-Lone-off	1,031,284	-80,158	91,978	1,152,216	-824	568	Dominated	161,918	-1,200
H1-MH2-LM3-L3	1,003,277	-108,165	63,970	1,152,258	-782	610	Dominated	104,927	-819
H1-MH2-LM2-L3	1,014,367	-97,075	75,060	1,152,314	-727	665	Dominated	112,813	-795
H1-MH2-LM2-L2	1,029,210	-82,232	89,904	1,152,462	-579	813	Dominated	110,538	-584
H1-MH1-LM3-L3‡	1,028,815	-82,627	89,509	1,152,545	-496	896	Dominated	99,873	-378
H1-MH1-LM2-L3	1,039,905	-71,537	11,090	1,152,600	-440	56	Dominated	199,140	-354
H1-MH1-LM1-L3	1,064,212	-47,230	35,397	1,152,668	-372	123	Dominated	286,891	-433
H1-MH1-LM2-L2‡	1,054,749	-56,693	25,934	1,152,748	-292	204	Dominated	127,333	-143
H1-MH1-LM1-L2	1,079,055	-32,387	24,306	1,152,816	-225	68	Dominated	359,088	-222
H1-MH1-LM1-L1‡ (status quo strategy)	1,111,442	NA	56,693	1,153,041	NA	292	NA	194,022	NA

‡ These strategies comprised the cost-effectiveness efficiency frontier. QALY, quality-adjusted life year; ICER, the incremental cost-effectiveness ratio; iNMB, incremental net monetary benefit

Table S21 Cost-effectiveness estimates for lung cancer screening scenarios ordered by QALYs (No discount)

Strategy	Costs per 100,000 people (CNY, thousand)	Incremental cost per 100,000 people (CNY, thousand)		QALYs per 100,000 people	Incremental QALYs per 100,000 people		ICER (CNY/QALY)		iNMB
		Vs the status quo strategy	Vs the strategy preceding it on the efficiency frontier		Vs the status quo strategy	Vs the strategy preceding it on the efficiency frontier	Vs the status quo strategy	Vs the strategy preceding it on the efficiency frontier	
No screening	1,419,876	-696,090	NA	1,847,540	-9,322	NA	Dominated	NA	-15,685
H1-MHnone-LMnone-Lnone	1,526,010	-589,957	106,134	1,849,243	-7,619	1,703	Dominated	62,324	-12,610
H1-MHone-off-LMone-off-Lnone	1,551,727	-564,240	131,850	1,849,437	-7,425	1,897	Dominated	69,504	-12,395
H1-MHone-off-LMnone-Lnone	1,541,542	-574,425	121,666	1,849,461	-7,401	1,921	Dominated	63,340	-12,236
H1-MHone-off-LMone-off-Lone-off	1,564,054	-551,912	144,178	1,849,506	-7,356	1,966	Dominated	73,334	-12,351
H1-MH3-LMone-off-Lnone	1,639,758	-476,209	219,882	1,851,439	-5,423	3,899	Dominated	56,392	-8,412
H1-MH3-LMnone-Lnone‡	1,629,574	-486,393	209,698	1,851,463	-5,399	3,923	Dominated	53,453	-8,252
H1-MH3-LMone-off-Lone-off	1,652,086	-463,881	22,512	1,851,508	-5,354	45	Dominated	498,130	-8,367
H1-MH2-LMone-off-Lnone	1,668,076	-447,891	38,502	1,851,880	-4,982	417	Dominated	92,302	-7,624
H1-MH2-LMnone-Lnone‡	1,657,891	-458,076	28,317	1,851,904	-4,958	441	Dominated	64,217	-7,464
H1-MH2-LM3-Lnone	1,697,650	-418,317	39,759	1,851,921	-4,941	17	Dominated	2,313,357	-7,820
H1-MH2-LMone-off-Lone-off	1,680,403	-435,563	22,512	1,851,949	-4,913	45	Dominated	498,130	-7,579
H1-MH1-LMone-off-Lnone	1,708,747	-407,219	50,856	1,852,266	-4,596	362	Dominated	140,572	-7,094
H1-MH1-LMnone-Lnone	1,698,563	-417,404	40,672	1,852,290	-4,573	386	Dominated	105,472	-6,934
H1-MH1-LMone-off-Lone-off	1,721,075	-394,892	63,184	1,852,335	-4,527	431	Dominated	146,663	-7,049
H1-MH3-LM3-Lnone	1,710,623	-405,344	52,732	1,852,422	-4,440	518	Dominated	101,772	-6,733
H1-MH3-LM3-Lone-off	1,722,951	-393,016	65,060	1,852,491	-4,371	587	Dominated	110,802	-6,688

H1-MH1-LM2-Lnone	1,770,627	-345,340	112,735	1,852,525	-4,337	621	Dominated	181,488	-7,082
H1-MH1-LM1-Lnone	1,821,643	-294,323	163,752	1,852,607	-4,255	703	Dominated	233,017	-7,394
H1-MH2-LM3-Lone-off	1,751,268	-364,698	93,377	1,852,932	-3,930	1,028	Dominated	90,822	-5,900
H1-MH2-LM2-Lnone	1,771,245	-344,722	113,354	1,853,082	-3,781	1,177	Dominated	96,269	-5,737
H1-MH2-LM2-Lone-off	1,783,573	-332,394	125,682	1,853,151	-3,712	1,246	Dominated	100,828	-5,693
H1-MH1-LM3-Lnone	1,779,612	-336,354	121,721	1,853,249	-3,613	1,345	Dominated	90,518	-5,415
H1-MH1-LM3-Lone-off	1,791,940	-324,027	134,049	1,853,318	-3,544	1,414	Dominated	94,818	-5,370
H1-MH1-LM2-Lone-off	1,824,245	-291,722	166,354	1,853,536	-3,326	1,632	Dominated	101,925	-5,163
H1-MH1-LM1-Lone-off	1,875,261	-240,705	217,370	1,853,618	-3,244	1,714	Dominated	126,843	-5,475
H1-MH3-LM3-L3‡	1,852,578	-263,389	194,687	1,854,926	-1,937	3,022	Dominated	64,431	-2,070
H1-MH2-LM3-L3‡	1,880,895	-235,072	28,317	1,855,367	-1,496	441	Dominated	64,217	-1,282
H1-MH2-LM2-L3	1,913,200	-202,767	32,304	1,855,585	-1,277	218	Dominated	147,937	-1,075
H1-MH1-LM3-L3	1,921,567	-194,400	40,672	1,855,752	-1,110	386	Dominated	105,472	-752
H1-MH1-LM2-L3	1,953,872	-162,095	72,976	1,855,971	-892	604	Dominated	120,825	-545
H1-MH1-LM1-L3	2,004,888	-111,079	123,993	1,856,052	-810	686	Dominated	180,864	-857
H1-MH2-LM2-L2‡	1,958,004	-157,963	77,109	1,856,097	-765	731	Dominated	105,554	-279
H1-MH1-LM2-L2‡	1,998,676	-117,291	40,672	1,856,483	-379	386	Dominant	105,472	251
H1-MH1-LM1-L2	2,049,693	-66,274	51,017	1,856,564	-298	82	Dominated	625,389	-61
H1-MH1-LM1-L1‡ (status quo strategy)	2,115,967	NA	117,291	1,856,862	NA	379	NA	309,120	NA

‡ These strategies comprised the cost-effectiveness efficiency frontier. QALY, quality-adjusted life year; ICER, the incremental cost-effectiveness ratio; iNMB, incremental net monetary benefit

Table S22 Cost-effectiveness estimates for lung cancer screening scenarios ordered by QALYs (Discount: 8%)

Strategy	Costs per 100,000 people (CNY, thousand)	Incremental cost per 100,000 people (CNY, thousand)		QALYs per 100,000 people	Incremental QALYs per 100,000 people		ICER (CNY/QALY)		iNMB
		Vs the status quo strategy	Vs the strategy preceding it on the efficiency frontier		Vs the status quo strategy	Vs the strategy preceding it on the efficiency frontier	Vs the status quo strategy	Vs the strategy preceding it on the efficiency frontier	
No screening	665,197	-346,602	NA	925,419	-1,667	NA	Dominated	NA	-582
H1-MHone-off-LMone-off-Lone-off	766,520	-245,279	101,323	925,901	-1,184	482	Dominated	210,122	-424
H1-MHnone-LMnone-Lnone	733,689	-278,110	68,493	925,912	-1,173	493	Dominated	138,820	-69
H1-MHone-off-LMone-off-Lnone	755,810	-255,989	90,613	925,919	-1,166	500	Dominated	181,214	-274
H1-MHone-off-LMnone-Lnone	746,402	-265,397	81,206	925,972	-1,113	553	Dominated	146,796	-51
H1-MH1-LM1-Lnone	896,525	-115,274	231,328	926,446	-640	1,027	Dominated	225,296	-401
H1-MH3-LMone-off-Lone-off	805,699	-206,100	140,503	926,460	-626	1,041	Dominant	135,005	541
H1-MH3-LMone-off-Lnone	794,989	-216,810	-10,710	926,477	-608	1,059	Dominant	-10,118	691
H1-MH3-LMnone-Lnone‡	785,582	-226,217	-20,118	926,530	-555	1,112	Dominant	-18,096	914
H1-MH2-LM3-Lnone	830,470	-181,329	24,770	926,532	-553	2	Dominant	13,610,356	470
H1-MH1-LM2-Lnone	870,319	-141,480	39,849	926,550	-536	19	Dominant	2,081,461	113
H1-MH2-LMone-off-Lone-off	821,220	-190,579	-9,249	926,563	-522	32	Dominant	-285,212	637
H1-MH2-LMone-off-Lnone	810,510	-201,289	-19,959	926,581	-505	50	Dominant	-397,178	787
H1-MH1-LMone-off-Lone-off	845,602	-166,197	15,132	926,587	-498	56	Dominant	267,858	451
H1-MH1-LMone-off-Lnone	834,892	-176,907	4,422	926,605	-481	74	Dominant	59,504	602
H1-MH3-LM3-Lone-off	835,943	-175,856	5,473	926,627	-458	96	Dominant	56,765	645
H1-MH2-LMnone-Lnone‡	801,103	-210,696	-29,367	926,634	-451	103	Dominant	-284,001	1,010

H1-MH1-LM1-Lone-off	917,519	-94,280	87,049	926,644	-442	10	Dominated	8,994,583	-130
H1-MH3-LM3-Lnone	825,233	-186,566	-5,237	926,645	-441	11	Dominant	-483,121	795
H1-MH1-LMnone-Lnone	825,484	-186,315	-4,986	926,658	-427	24	Dominant	-207,189	825
H1-MH2-LM2-Lone-off	866,932	-144,867	36,462	926,724	-362	90	Dominant	406,566	570
H1-MH2-LM3-Lone-off	851,464	-160,335	20,994	926,730	-355	96	Dominant	217,735	741
H1-MH2-LM2-Lnone	856,222	-155,577	25,752	926,741	-344	108	Dominant	239,540	720
H1-MH1-LM2-Lone-off	891,313	-120,486	60,844	926,748	-338	114	Dominant	534,907	385
H1-MH1-LM3-Lone-off	875,845	-135,953	45,376	926,754	-331	120	Dominant	376,611	556
H1-MH1-LM3-Lnone	865,135	-146,664	34,666	926,772	-313	138	Dominant	250,642	706
H1-MH3-LM3-L3‡	880,537	-131,262	50,067	927,080	-5	447	Dominant	112,128	1,301
H1-MH1-LM1-L1 (status quo strategy)	1,011,799	NA	131,262	927,085	NA	5	NA	26,736,048	NA
H1-MH1-LM1-L3	962,113	-49,686	-49,686	927,097	12	17	Cost-saving	-2,982,059	525
H1-MH1-LM1-L2	980,759	-31,040	-31,040	927,152	66	71	Cost-saving	-436,344	471
H1-MH2-LM2-L3	911,525	-100,274	-100,274	927,177	92	97	Cost-saving	-1,037,313	1,226
H1-MH2-LM3-L3‡	896,057	-115,741	-115,741	927,184	98	103	Cost-saving	-1,119,302	1,397
H1-MH1-LM2-L3	935,907	-75,892	-75,892	927,201	116	17	Cost-saving	-4,380,528	1,040
H1-MH1-LM3-L3	920,439	-91,360	-15,468	927,208	123	24	Cost-saving	-642,800	1,211
H1-MH2-LM2-L2‡	930,172	-81,627	9,733	927,232	146	48	Cost-saving	203,879	1,172
H1-MH1-LM2-L2‡	954,553	-57,246	34,114	927,256	170	24	Cost-saving	1,417,688	986

‡ These strategies comprised the cost-effectiveness efficiency frontier. QALY, quality-adjusted life year; ICER, the incremental cost-effectiveness ratio; iNMB, incremental net monetary benefit

Table S23 Cost-effectiveness estimates for lung cancer screening scenarios ordered by QALYs (Quit smoking with being screened)

Strategy	Costs per 100,000 people (CNY, thousand)	Incremental cost per 100,000 people (CNY, thousand)		QALYs per 100,000 people	Incremental QALYs per 100,000 people		ICER (CNY/QALY)		iNMB
		Vs the status quo strategy	Vs the strategy preceding it on the efficiency frontier		Vs the status quo strategy	Vs the strategy preceding it on the efficiency frontier	Vs the status quo strategy	Vs the strategy preceding it on the efficiency frontier	
No screening	845,168	-418,706	NA	1,150,366	-4,018	NA	Dominated	NA	-5,574
H1-MHnone-LMnone-Lnone	921,744	-342,130	76,576	1,151,377	-3,007	1,011	Dominated	75,736	-3,884
H1-MHone-off-LMnone-Lnone	933,584	-330,290	88,416	1,151,705	-2,679	1,339	Dominated	66,015	-3,205
H1-MHone-off-LMone-off-Lnone	940,413	-323,461	95,246	1,152,052	-2,332	1,686	Dominated	56,489	-2,431
H1-MHone-off-LMone-off-Lone-off‡	949,918	-313,956	104,750	1,152,257	-2,126	1,892	Dominated	55,372	-2,026
H1-MH3-LMnone-Lnone	984,448	-279,426	34,530	1,152,550	-1,833	293	Dominated	117,884	-1,660
H1-MH2-LMnone-Lnone	1,003,008	-260,866	53,090	1,152,722	-1,661	465	Dominated	114,175	-1,427
H1-MH1-LMnone-Lnone	1,031,903	-231,971	81,985	1,152,807	-1,577	550	Dominated	149,196	-1,511
H1-MH3-LMone-off-Lnone	991,278	-272,596	41,360	1,152,897	-1,487	640	Dominated	64,658	-886
H1-MH1-LM1-Lnone	1,111,770	-152,104	161,852	1,152,945	-1,439	687	Dominated	235,506	-1,975
H1-MH2-LM3-Lnone	1,031,556	-232,318	81,638	1,152,965	-1,419	707	Dominated	115,451	-1,125
H1-MH1-LM2-Lnone	1,079,740	-184,134	129,823	1,153,054	-1,330	797	Dominated	162,959	-1,389
H1-MH2-LMone-off-Lnone	1,009,837	-254,037	59,920	1,153,069	-1,315	812	Dominated	73,816	-653
H1-MH3-LMone-off-Lone-off‡	1,000,782	-263,092	50,864	1,153,103	-1,281	845	Dominated	60,171	-481
H1-MH3-LM3-Lnone	1,029,572	-234,302	28,790	1,153,152	-1,232	49	Dominated	581,842	-649

H1-MH1-LMone-off-Lnone	1,038,733	-225,141	37,951	1,153,154	-1,230	51	Dominated	744,895	-737
H1-MH2-LMone-off-Lone-off	1,019,341	-244,532	18,559	1,153,275	-1,109	172	Dominated	107,862	-249
H1-MH2-LM2-Lnone	1,067,421	-196,453	66,639	1,153,329	-1,054	227	Dominated	294,137	-597
H1-MH3-LM3-Lone-off	1,039,076	-224,797	38,294	1,153,358	-1,026	255	Dominated	150,099	-244
H1-MH1-LMone-off-Lone-off	1,048,237	-215,637	47,455	1,153,359	-1,024	257	Dominated	184,941	-332
H1-MH1-LM3-Lnone	1,077,027	-186,847	76,245	1,153,409	-975	306	Dominated	249,105	-500
H1-MH1-LM1-Lone-off	1,137,850	-126,024	137,068	1,153,510	-874	407	Dominated	336,507	-862
H1-MH2-LM3-Lone-off	1,057,636	-206,238	56,854	1,153,530	-854	427	Dominated	133,087	-12
H1-MH2-LM2-Lone-off	1,076,925	-186,949	76,143	1,153,535	-849	432	Dominated	176,174	-193
H1-MH1-LM3-Lone-off	1,086,531	-177,343	85,749	1,153,615	-769	512	Dominated	167,570	-96
H1-MH1-LM2-Lone-off	1,105,821	-158,053	105,039	1,153,620	-764	517	Dominated	203,275	-276
H1-MH3-LM3-L3‡	1,101,052	-162,822	100,270	1,154,135	-249	1,032	Dominant	97,131	1,024
H1-MH1-LM1-L3	1,199,826	-64,048	98,774	1,154,287	-97	152	Dominant	648,980	406
H1-MH2-LM3-L3‡	1,119,611	-144,262	18,559	1,154,307	-77	172	Dominant	107,862	1,256
H1-MH2-LM2-L3	1,138,901	-124,973	19,289	1,154,312	-72	5	Dominant	3,850,162	1,076
H1-MH1-LM1-L1 (status quo strategy)	1,263,874	NA	144,262	1,154,384	NA	77	NA	1,881,574	NA
H1-MH1-LM3-L3‡	1,148,507	-115,367	28,895	1,154,392	8	85	Cost-saving	341,847	1,173
H1-MH1-LM2-L3	1,167,796	-96,078	19,289	1,154,397	13	5	Cost-saving	3,850,162	992
H1-MH1-LM1-L2	1,224,283	-39,591	75,776	1,154,411	27	19	Cost-saving	3,947,698	462
H1-MH2-LM2-L2‡	1,163,358	-100,516	14,851	1,154,436	52	44	Cost-saving	336,964	1,131
H1-MH1-LM2-L2‡	1,192,253	-71,620	28,895	1,154,520	136	85	Cost-saving	341,847	1,048

‡ These strategies comprised the cost-effectiveness efficiency frontier. QALY, quality-adjusted life year; ICER, the incremental cost-effectiveness ratio; iNMB, incremental net monetary benefit

Probabilistic sensitivity analyses

Results of probabilistic sensitivity analyses if individuals' smoking behaviors were not changed were shown as Fig 1~Fig 6. Resluts of probabilistic sensitivity analyses if individuals quit smoking with being screened were shown as Fig 7~Fig 13.

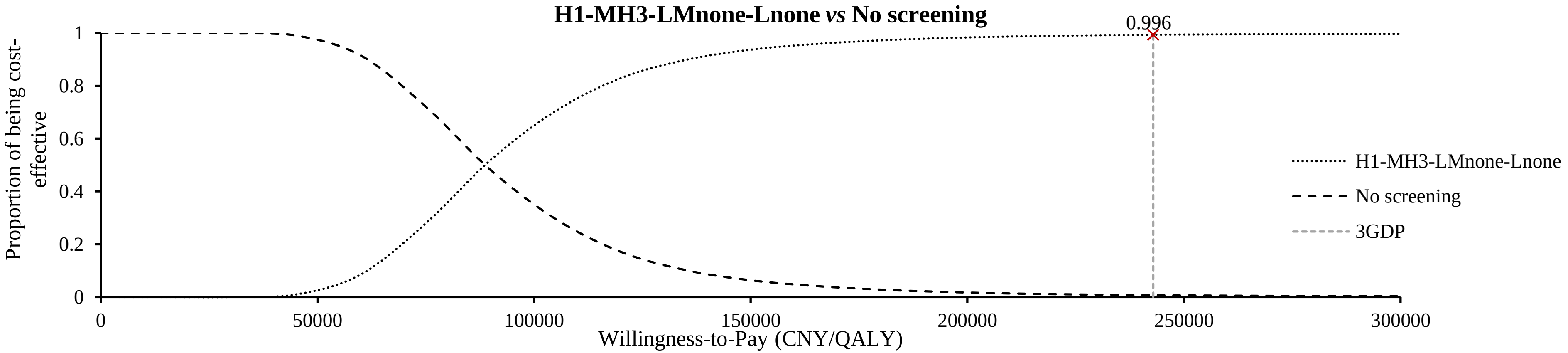

Fig. S1 Cost-effectiveness acceptability curves of H1-MH3-LMnone-Lnone *vs* No screening. QALY, quality-adjusted life year; GDP, gross domestic product.

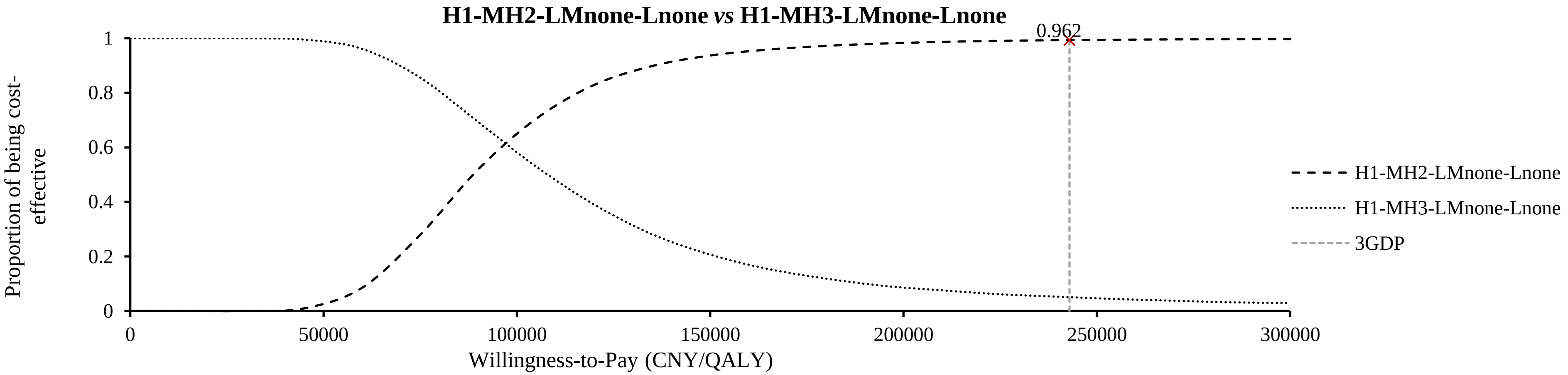

Fig. S2 Cost-effectiveness acceptability curves of H1-MH2-LMnone-Lnone *vs* H1-MH3-LMnone-Lnone. QALY, quality-adjusted life year; GDP, gross domestic product.

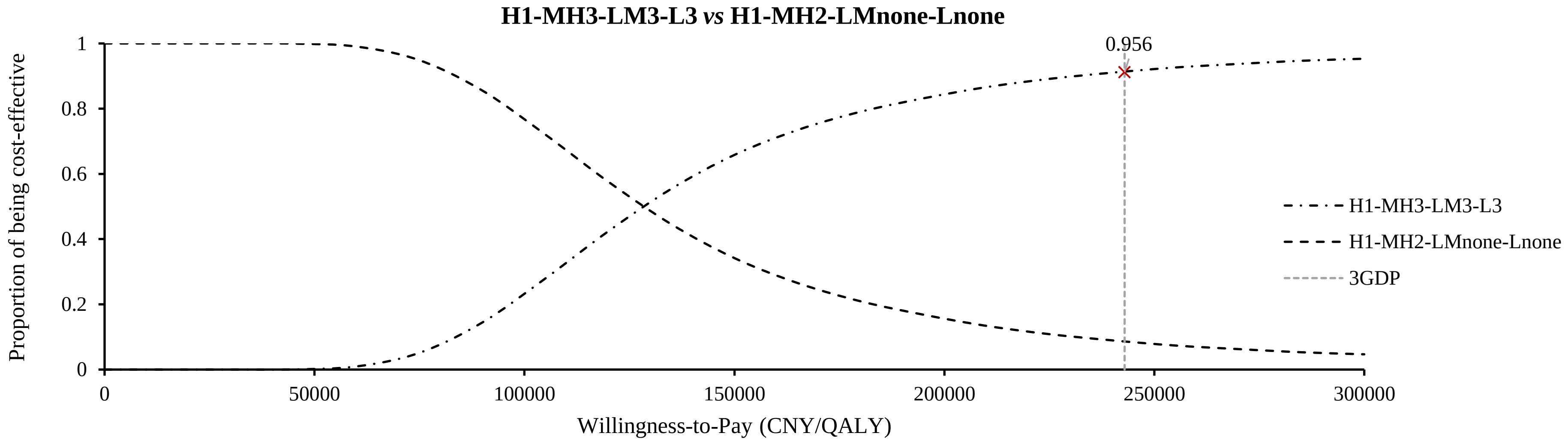

Fig. S3 Cost-effectiveness acceptability curves of H1-MH3-LM3-L3 *vs* H1-MH2-LMnone-Lnone. QALY, quality-adjusted life year; GDP, gross domestic product.

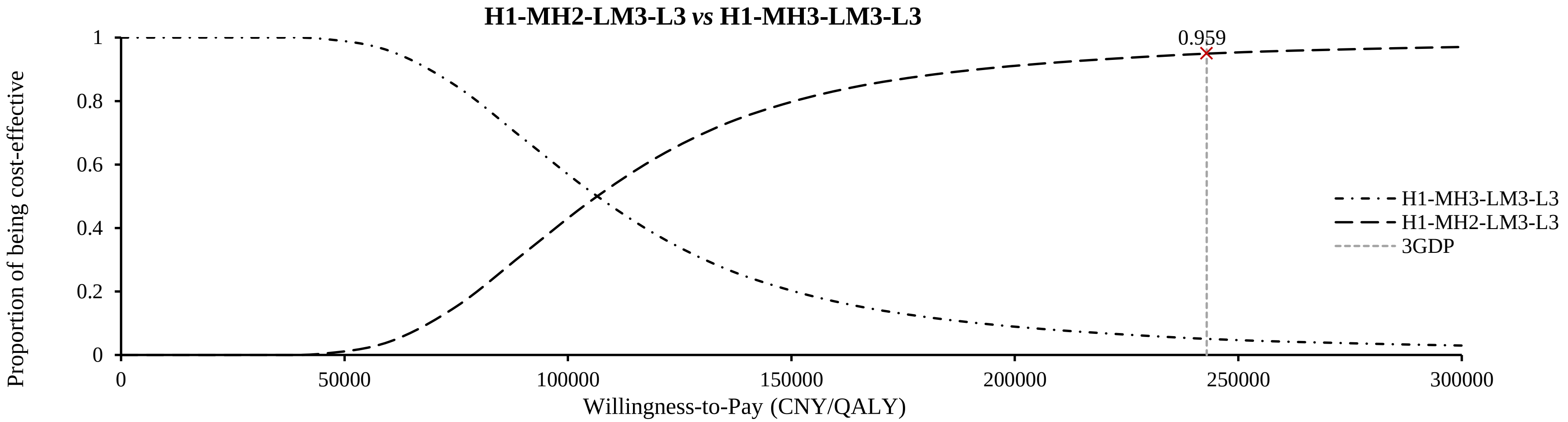

Fig. S4 Cost-effectiveness acceptability curves of H1-MH2-LM3-L3 *vs* H1-MH3-LM3-L3. QALY, quality-adjusted life year; GDP, gross domestic product.

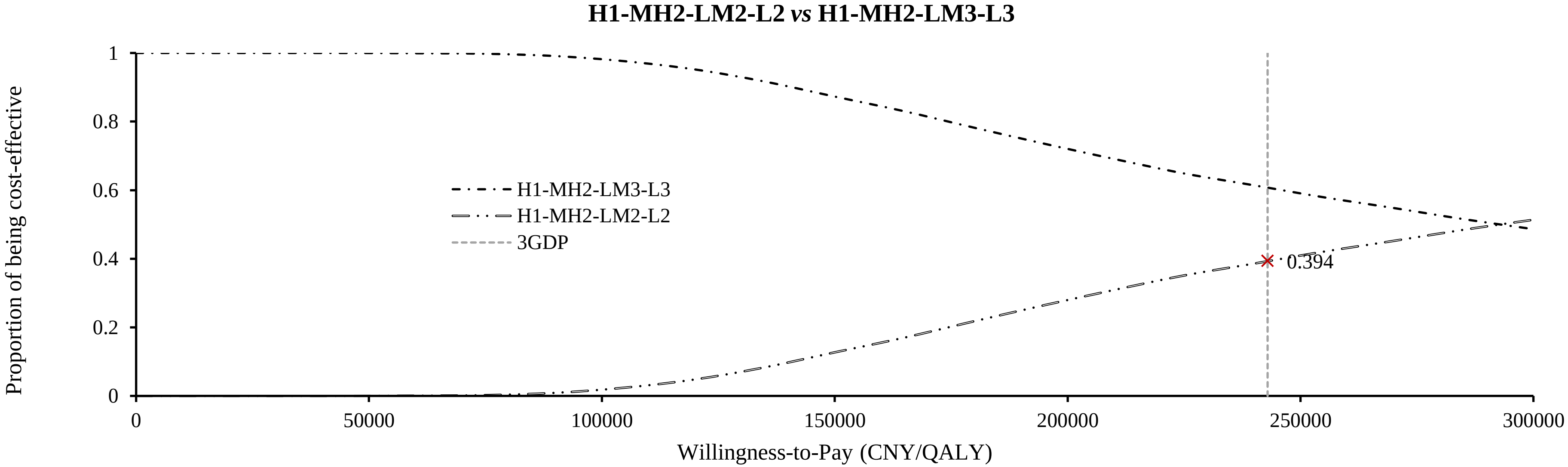

Fig. S5 Cost-effectiveness acceptability curves of H1-MH2-LM2-L2 *vs* H1-MH2-LM3-L3. QALY, quality-adjusted life year; GDP, gross domestic product.

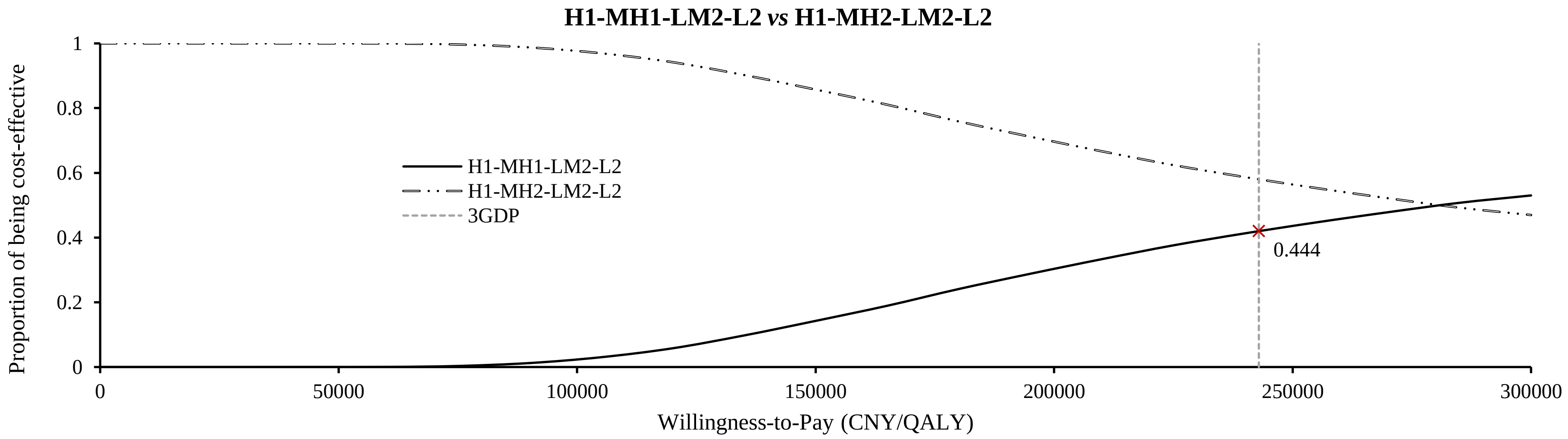

Fig. S6 Cost-effectiveness acceptability curves of H1-MH1-LM2-L2 *vs* H1-MH2-LM2-L2. QALY, quality-adjusted life year; GDP, gross domestic product.

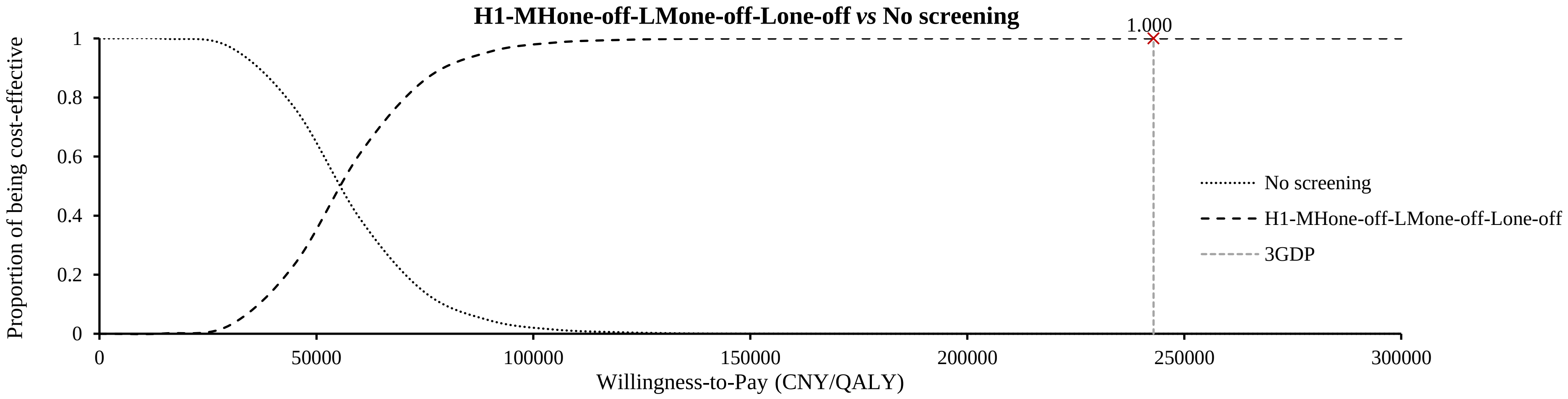

Fig. S7 Cost-effectiveness acceptability curves of H1-MHone-off-LMone-off-Lone-off *vs* No screening if individuals quit smoking with being screened. QALY, quality-adjusted life year; GDP, gross domestic product.

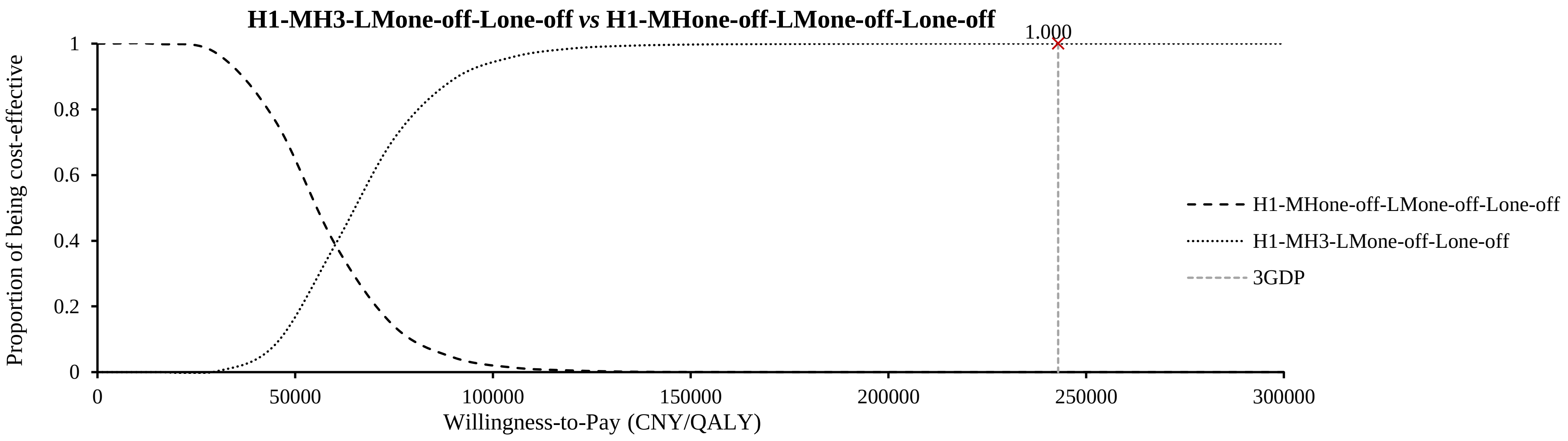

Fig. S8 Cost-effectiveness acceptability curves of H1-MH3-LMone-off-Lone-off *vs* H1-MHone-off-LMone-off-Lone-off if individuals quit smoking with being screened. QALY, quality-adjusted life year; GDP, gross domestic product.

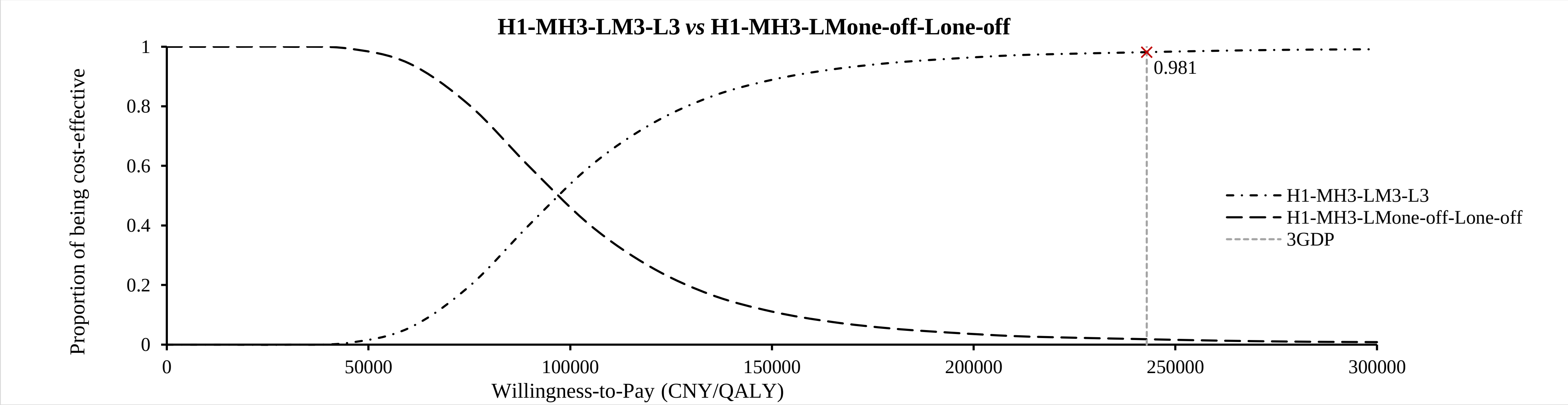

Fig. S9 Cost-effectiveness acceptability curves of H1-MH3-LM3-L3 *vs* H1-MH3-LMone-off-Lone-off if individuals quit smoking with being screened. QALY, quality-adjusted life year; GDP, gross domestic product.

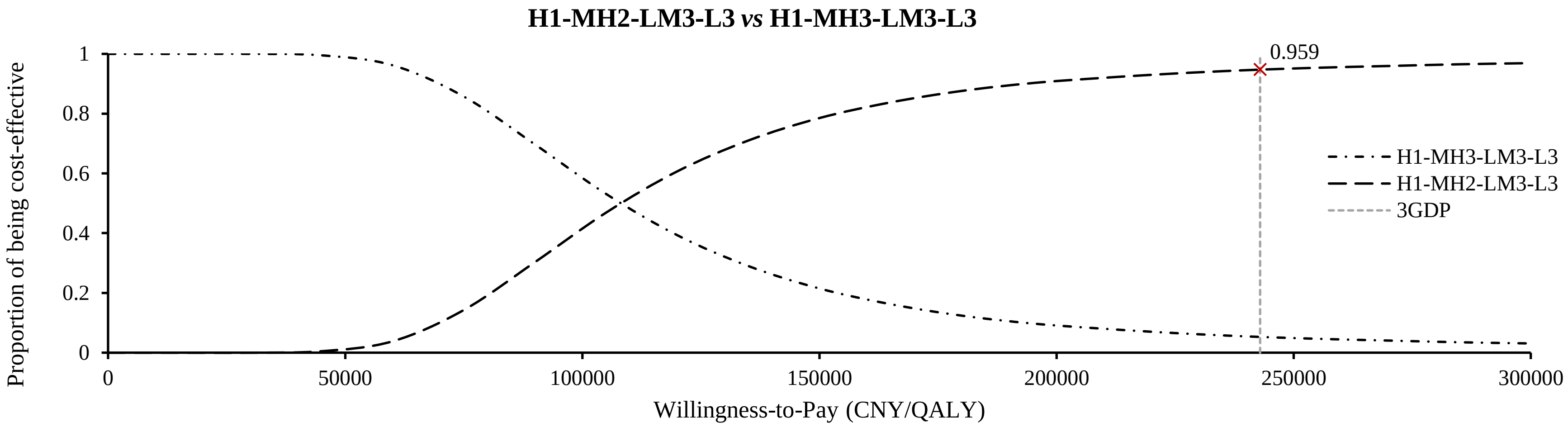

Fig. S10 Cost-effectiveness acceptability curves of H1-MH2-LM3-L3 *vs* H1-MH3-LM3-L3 if individuals quit smoking with being screened. QALY, quality-adjusted life year; GDP, gross domestic product.

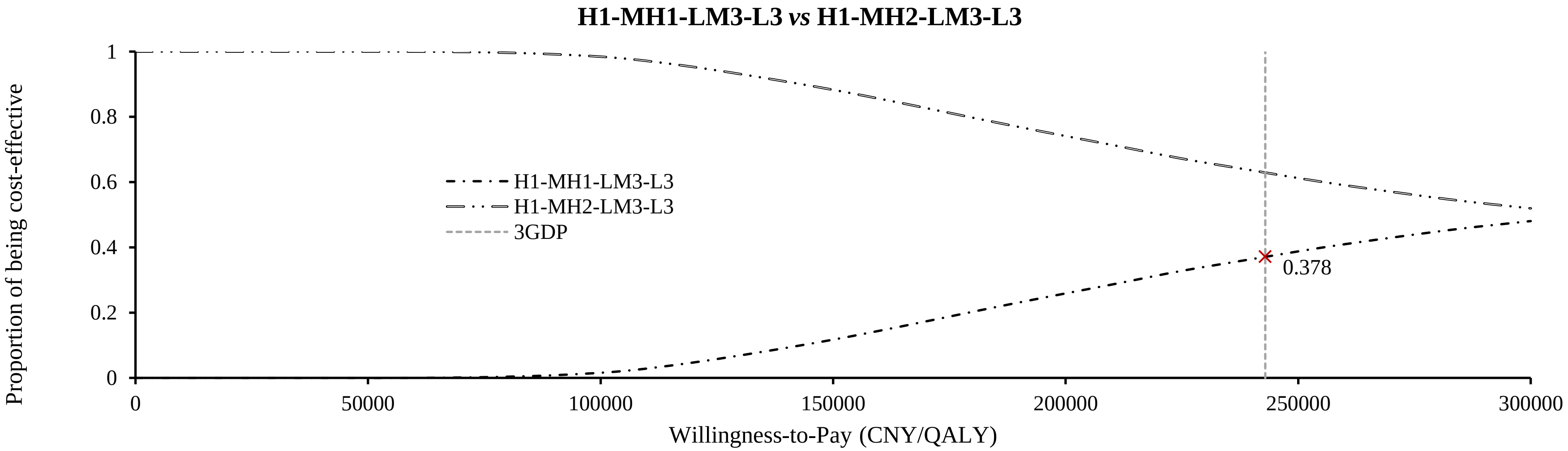

Fig. S11 Cost-effectiveness acceptability curves of H1-MH1-LM3-L3 *vs* H1-MH2-LM3-L3 if individuals quit smoking with being screened. QALY, quality-adjusted life year; GDP, gross domestic product.

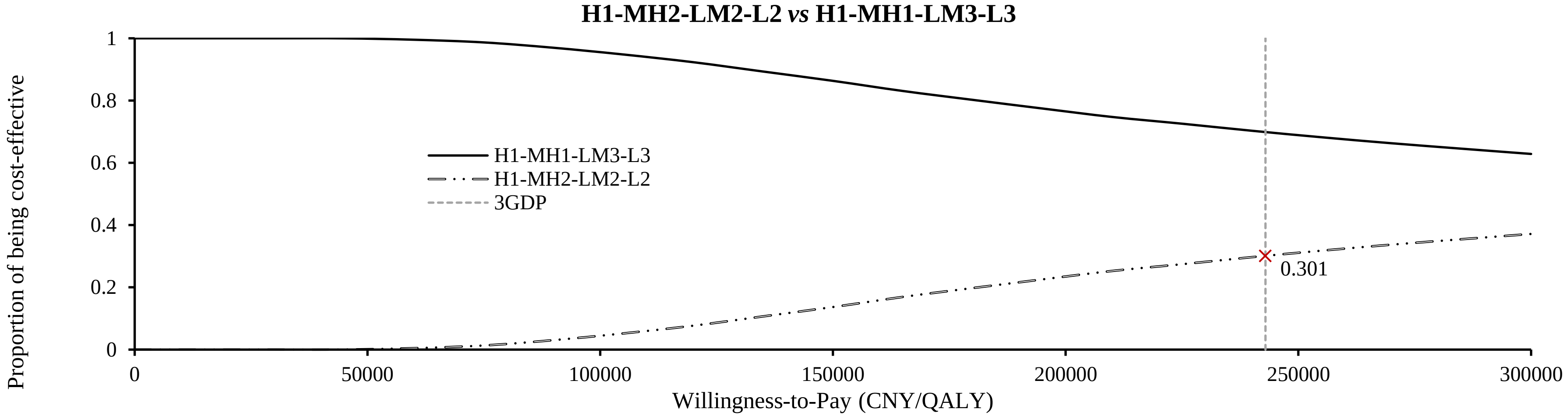

Fig. S12 Cost-effectiveness acceptability curves of H1-MH2-LM2-L2 *vs* H1-MH1-LM3-L3 if individuals quit smoking with being screened. QALY, quality-adjusted life year; GDP, gross domestic product.

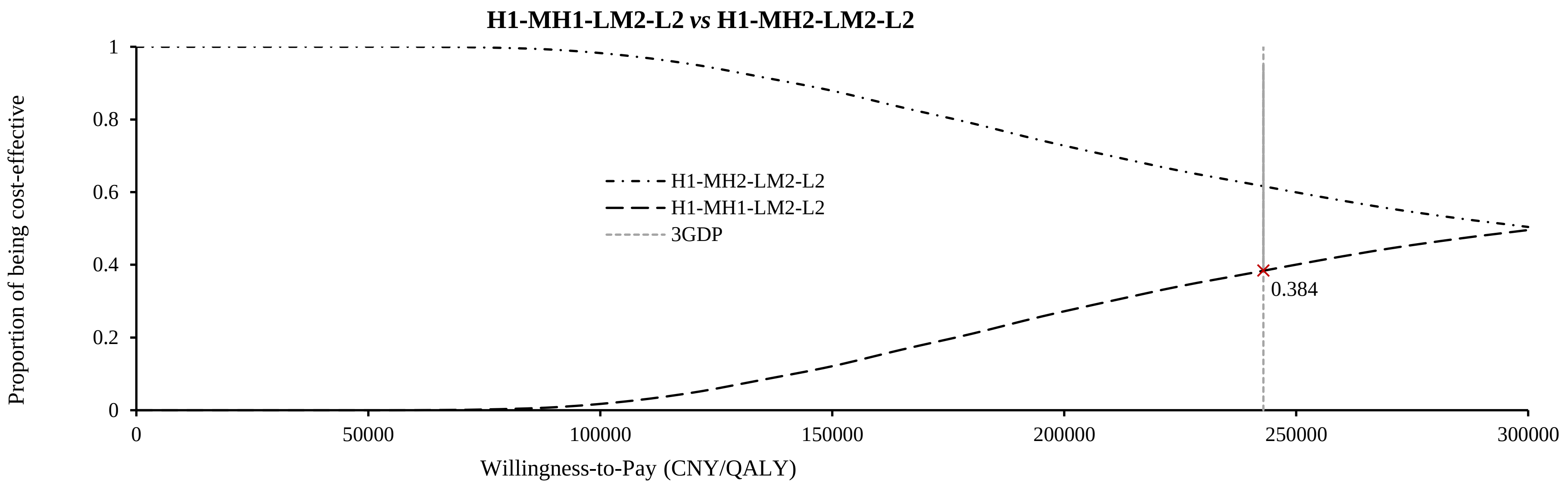

Fig. S13 Cost-effectiveness acceptability curves of H1-MH1-LM2-L2 *vs* H1-MH2-LM2-L2 if individuals quit smoking with being screened. QALY, quality-adjusted life year; GDP, gross domestic product.
