# Supplementary material for: Risk-based lung cancer screening in heavy smokers: a benefit–harm and cost-effectiveness modeling study
Source: BMC Med. 2024 Feb 19;22:73. doi: 10.1186/s12916-024-03292-4 (PMC10875747; doi:10.1186/s12916-024-03292-4)
Supplement: Supplementary file 2 — Additional file 2. Overview of the sample survey on lung cancer care costs. [file 12916_2024_3292_MOESM2_ESM.pdf]

SUPPLEMENTAL MATERIAL—ADDITIONAL FILE 2

Risk-based lung cancer screening in heavy smokers: a benefit–harm and cost-effectiveness modelling study

Yin Liu[1], Huifang Xu [1], Lihong Lv[1], Xiaoyang Wang[1], Ruihua Kang [1], Xiaoli Guo[1], Hong Wang [1], Liyang Zheng[1], Hongwei Liu [1], Lanwei Guo [1], Qiong Chen [1], Shuzheng Liu [1], Youlin Qiao [1,2*], Shaokai Zhang [1*]

[1]Department of Cancer Epidemiology, The Affiliated Cancer Hospital of Zhengzhou University & Henan Cancer Hospital, Zhengzhou, 450008, China

[2]Center for Global Health, School of Population Medicine and Public Health, Chinese Academy of Medical Sciences and Peking Union Medical College, Beijing 100005, China

Corresponding Author:

Youlin Qiao, Email: qiaoy@cicams.ac.cn; Fax:(0371)-65587361;Tel: (0371)-65587361

Shaokai Zhang, Email: shaokaizhang@126.com; Fax:(0371)-65587361; Tel: (0371)-65587361

Overview of the sample survey on lung cancer care costs

Data sources

A survey was conducted at Henan cancer hospital from June 2022 to June 2023 to estimate the medical expenditure incurred for lung cancer care. Henan cancer hospital is a public, specialized, educational hospital that provides care for patients with all types of cancers. The survey collected clinical and annual medical expense information for both inpatient and outpatient visits of lung cancer patients at the hospital. Only medical expenses incurred before medical insurance reimbursement were included in the data.

Medical expenditure data and inclusion/exclusion criteria

The inpatients simultaneously fulfilling the following conditions were included: (1) diagnosed with lung cancer as the primary tumor; (2) main treatment and expenses occurred in Henan cancer hospital; (3) last discharge date was between January 1, 2016 and June 30, 2023; and (4) patients' basic information, expenditure information, and clinical information (clinical diagnosis, treatment programs, and pathological information) were available and intact.

Individuals were excluded if they: (1) had been diagnosed with lung cancer for less than 1 year; (2) had two or more primary cancers; (3) only received a diagnosis or postoperative follow-up in the investigated hospital.

A total of 350 eligible lung cancer patients were included in the study. Among them, 116 (33.1%), 72 (20.6%), 60 (17.1%), and 102 (29.1%) were classified as stage I, stage II, stage III, and stage IV patients, respectively.

Data analysis

All costs were converted to 2022 Chinese yuan (CNY) using the year-specific medical component of the consumer price index. Descriptive statistical techniques were employed to analyze the stage-specific expenses incurred by patients in the first year, second year and beyond after diagnosis of lung cancer. All data were analyzed using R software V 4.3.
